# Supplementary material for: Human papillomavirus integration perspective in small cell cervical carcinoma
Source: Nat Commun. 2022 Oct 10;13:5968. doi: 10.1038/s41467-022-33359-w (PMC9550834; doi:10.1038/s41467-022-33359-w)
Supplement: Supplementary file 1 — Supplementary Information file [file 41467_2022_33359_MOESM1_ESM.pdf]

SUPPLEMENTARY INFORMATION

Human papillomavirus integration perspective in small cell cervical carcinoma

Supplementary Notes

Supplementary Note 1 | SCCC sample preparation

Supplementary Note 2 | DNA and RNA extractions

Supplementary Note 3 | HPV genotyping

Supplementary Note 4 | Sequence Data Generation

Supplementary Note 5 | Analysis on VCS data

Supplementary Note 6 | HPV variant assignment from WGS analysis

Supplementary Note 7 | HPV integration profiles from WGS analysis

Supplementary Note 8 | The verification of HPV integration positions

Supplementary Note 9 | PCR and Sanger sequencing verification

Supplementary Note 10 | Local haplotype construction from WGS analysis

Supplementary Note 11 | Haplotype information of HPV-integrated SCCC samples

Supplementary Note 12 | Preprocess and alignment of 10x long-range sequencing data

Supplementary Note 13 | Linkage among the breakages in local haplotypes

Supplementary Note 14 | The verification of virus-human fusion genes

Supplementary Note 15 | The verification of ASEs

Supplementary Note 16 | Linkage of ASEs and local haplotypes

Supplementary Note 17 | Deciphering mutational signatures operative in SCCC

Supplementary Note 18 | OncoScan CNV FFPE assay

Supplementary Note 19 | Signaling pathways and survival analyses

Supplementary Note 20 | Immunohistochemical staining

Supplementary Figures

Supplementary Figure 1 | Genomic analysis in SCCC

Supplementary Figure 2 | Panorama of HPV analysis in SCCC WGS samples and HPV viral gene expression

Supplementary Figure 3 | Constructed individual HPV genomes in SCCC WGS samples

Supplementary Figure 4 |Panorama of HPV analysis in SCCC VCS samples

Supplementary Figure 5 | HPV genome depth comparison from VCS analysis between 150 SCCC FFPE samples

Supplementary Figure 6 | HPV integration hotspots

Supplementary Figure 7 | Presentative local haplotype of HPV integration sites of T011

Supplementary Figure 8 | Presentative local haplotype of HPV integration sites of T019

Supplementary Figure 9 | Presentative local haplotype of HPV integration sites of T003

Supplementary Figure 10 | Presentative local haplotype of HPV integration sites of T010

Supplementary Figure 11 | Presentative local haplotype of HPV integration sites of T004

Supplementary Figure 12 | Presentative local haplotype of HPV integration sites of T014

Supplementary Figure 13 | Presentative local haplotype of HPV integration sites of T017

Supplementary Figure 14 | Presentative local haplotype of HPV integration sites of T018

Supplementary Figure 15 | Presentative local haplotype of HPV integration sites of T001

Supplementary Figure 16 | Presentative local haplotype of HPV integration sites of T002

Supplementary Figure 17 | Presentative local haplotype of HPV integration sites of T005

Supplementary Figure 18 | Presentative local haplotype of HPV integration sites of T006

Supplementary Figure 19 | Presentative local haplotype of HPV integration sites of T009

Supplementary Figure 20 | Features of HPV16 integrated local haplotypes

Supplementary Figure 21 | Optimized local haplotypes of HPV integration sites

Supplementary Figure 22 | Distribution of 10x linked-reads barcode spanning size in T011

Supplementary Figure 23 | Distribution of 10x linked-reads barcode spanning size in T014

Supplementary Figure 24 | Distribution of 10x linked-reads barcode spanning size in T017

Supplementary Figure 25 | Distribution of 10x linked-reads barcode spanning size in T018

Supplementary Figure 26 | Correlation of 10x linked-reads and local haplotypes of four samples

Supplementary Figure 27 | Mutation and mutation signatures from WGS and WES analysis in SCCC samples

Supplementary Figure 28 | CNA landscape and SV signatures from WGS analysis in SCCC samples

Supplementary Figure 29 | Survival analyses stratified by MYC amplification in SCCC samples

Supplementary Figure 30 | Fusion events cartoons with validated sequences in SCCC samples

Supplementary Figure 31 | STRING analyses of DEGs in SCCC samples

Supplementary Figure 32 | GSEA analyses of DEGs in SCCC samples

Supplementary Figure 33 | Structural variants from WGS analysis in SCCC samples

Supplementary Figure 34 | Immunohistochemical staining in SCCC FFPE samples

Supplementary Tables

Supplementary Table 1| Clinical statistics in SCCC patients

Supplementary Table 2| VCS data summary

Supplementary Table 3| RNA-seq data summary

Supplementary Table 4| 10x long-range sequencing data summary

Supplementary Table 5| HPV18gene expression in RNA-seq analysis

Supplementary Table 6| RNA and protein expressions of MYC, INSM1 and ASCL1

Supplementary Table 7| The 5-year OS rates of SCCC patients whose HPV integration sites were located on genes or gene families than those without HPV integration.

Supplementary Table 8| The 5-year DFS rates of SCCC patients whose HPV integration sites were located on genes or gene families than those without HPV integration.

Supplementary Table 9| The immunohistochemical staining of gene families with HPV integration.

Supplementary Notes

Supplementary Note 1| SCCC sample preparation

We collected fresh frozen or formalin fixed paraffin embedded (FFPE) samples of 214 SCCC patients, which were provided by fifteen collaborating Chinese hospitals in China from 2007 to 2015 as fresh-frozen tissue specimen, genomic DNA extracted from fresh-frozen materials or as FFPE sections (Supplementary Figure1a and Supplementary Data 1). The protocol was approved by the Ethics Committee of Tongji Hospital, Tongji Medical College, Huazhong University of Science and Technology, P. R. China. All patients provided written informed consent. All samples obtained by surgical resection (n=208) or biopsy (n=6) were histomorphological diagnosed as pure or mixed SCCC (n=180 or n=34) by H&E staining and immunohistochemistry positivity of at least one neuroendocrine marker for Chromogranin A (CgA), synaptophysin (SYN) and neuron specific enolase (NSE), and CD56, which used as a selection criterion for clinical SCCC diagnosis[1-3]. These SCCC samples were reviewed by at least two independent pathologists. The fresh-frozen SCCC samples obtained surgically resection and biopsies were primary tumors, snap-frozen in liquid nitrogen after tissue sampling and stored at -150C°. All tumor samples were preceded by micro-dissection and pathologically assessed to have a purity of at least 70% and no extensive signs of necrosis. Matching normal material was provided in the form of EDTA-anticoagulated blood, as well as adjacent non-tumorigenic or normal cervical tissues (Supplementary Figure1a). The matched normal tissue was also confirmed to be free of tumor contaminants by pathological assessment. Furthermore, tumor and matching normal materials were confirmed to be acquired from the same patient by short tandem repeat (STR) analysis or confirmed by sequencing analyses.

Whole-genome sequencing (WGS) on 16 pairs of SCCC fresh-frozen tumor-control paired samples was performed. We further studied the copy-number alterations of a total of 132 FFPE tumor samples by OncoScan microarrays. Additionally, RNA sequencing was applied by using of fresh-frozen 19 tumor samples and 18 non-tumor cervix tissues as controls, while whole-exome sequencing for 10 pairs of SCCC FFPE samples (adjacent non-tumor tissues as controls). Furthermore, primary findings on HPV integration sites, fusion genes and protein expression of potential driver genes were further studied in the extended cohort consisting of 214 SCCC cases, respectively. A total of 208 tumor samples were available for HPV genotyping, and 214

tumor FFPE slices (matched FFPE adjacent non-tumor tissues as controls) were available for immunohistochemistry staining (Supplementary Figure 1a and Supplementary Data 1).

The reports described the details of the clinical data sources and the methods used for this analysis. Briefly, the demographics and clinical data were collected from patient records by trained gynecological oncology staff using standardized data collection and quality control procedures. At the time of enrollment and during follow-up examinations the following tests were performed to determine patient status before and after treatment: gynecological examination, liquid-based thin-layer cytology and human papilloma virus tests, ultrasonographic evidence or computer tomography/magnetic resonance imaging, and so on[4,5]. The median follow-uptime for SCCC patients was 20.0 months, and 64.0% of the patients were alive at the time of the last follow-up (Supplementary Table 1).

Supplementary Note 2| DNA and RNA extractions

Nucleic acids were extracted from fresh-frozen tissue specimens which were processed to 6-10 sections each of 40μm thickness at a cryostat microtome maintaining a temperature of -25C° (Leica). In the case of FFPE samples, 5-8 sections of 10μm thickness were prepared. DNA was extracted from fresh-frozen tissues and EDTA-anticoagulated blood (QIAamp DNA Mini Kit, QIAGEN) or FFPE samples (GeneRead DNA FFPE Kit, QIAGEN) according to the instructions of the manufacturer. Genomic DNA from fresh-frozen samples with evident signs of degradation were excluded from further sequencing studies. RNA was extracted from fresh frozen samples preserved in RNA later (QIAGEN) by using Qiagen RNAeasy Mini Kit following the protocol of the manufacturer.

Supplementary Note 3| HPV genotyping

A PCR-Based Mass Spectrometry System for high-Risk (HR) HPV was used for HPV detection and genotyping[6].Fourteen HR-HPVs were diagnosed, including HPV types 16, 18, 31, 33, 35, 39, 45, 51, 52, 56, 58, 59,66, and 68, based on the amplification of a fragment of the L1 gene region using GP5+/GP6+ consensus primers and the subsequent use of a type-specific extension primer inside the GP5+/GP6+ to distinguish the 14 HPVs simultaneously in one reaction. The Mass-ARRAY (Sequenom, San Diego, CA) technique based on the matrix-

assisted laser desorption/ionization time-of-flight mass spectrometry (MALDI-TOF-MS) platform was used to analyze the extension products.

Supplementary Note 4| Sequence Data Generation

Supplementary Note 4A. Whole Genome Sequencing Library Construction and sequencing

A total amount of 0.5 μg DNA per sample was used for the DNA library preparations. Sequencing library was generated using Truseq Nano DNA HT Sample Prep Kit following manufacturer's recommendations and index codes were added to each sample (Illumina Inc. USA). Briefly, genomic DNA sample was fragmented by sonication to a size of 350 bp. Then DNA fragments were end polished, A-tailed, and ligated with the full-length adapter for Illumina sequencing, followed by further PCR amplification. After PCR products were purified (AMPure XP system), libraries were analyzed for size distribution by Agilent 2100 Bioanalyzer and quantified by real-time PCR (3nM).

The clustering of the index-coded samples was performed on a cBot Cluster Generation System using HiSeq X PE Cluster Kit V2.5 according to the manufacturer's instructions(Illumina Inc. USA). After cluster generation, the DNA libraries were sequenced on Illumina HiSeq platform and 150 bp paired-end reads were generated.

The original fluorescence image files obtained from HiSeq platform are transformed to short reads (Raw data) by base calling and these short reads are recorded in FASTQ format, which contains sequence information and corresponding sequencing quality information. Sequence artifacts, including reads containing adapter contamination, low-quality nucleotides and unrecognizable nucleotide (N), undoubtedly set the barrier for the subsequent reliable bioinformatics analysis. The steps of data processing were as follows: 1) Discard a paired reads if either one read contains adapter contamination (>10 nucleotides aligned to the adapter, allowing ≤ 10% mismatches); 2) Discard a paired reads if more than 10% of bases are uncertain in either one read; 3) Discard a paired reads if the proportion of low quality (Phred quality <5) bases is over 50% in either one read. All the downstream bioinformatics analyses were based on the high quality clean data, which were retained after these steps.

Supplementary Note 4B. cDNA Library Construction

The mRNA was isolated from total RNA by using oligo (dT) containing beads, then purified and fragmented in fragmentation buffer. The fragmented sequences were used as templates and the first-strand cDNAs were synthesized via random hexamer-primers. The buffer, dNTP, RNase H and DNA polymerase I were used to synthesized the second-strand. The double-stranded cDNA segments were further purified by QIAquick PCR extraction kit (vendor) and eluted with EB buffer for end repair and addition of A base. Then, Illumine sequencing adaptors were ligated to these short fragments. The cDNA fragments of selected size were gel-purified and amplified by PCR. The constructed libraries were sequenced on Illumina HiSeq platform.

Supplementary Note 4C. Whole Exome Sequencing Library Construction

Quantified genomic DNA were extracted from tumor and matched blood samples and fragmented by Covar is technology to generate DNA fragments of 200~300 bp. Adapters were ligated to two ends of DNA fragments and amplified via ligation-mediated PCR, and hybridize to Agilent 51Mb human exome capture chip (v4.0). The Agilent 51Mb represents ~18,560 genes (93% of known, non-repetitive protein coding genes) and spanning ~1% of the genome (32.7Mb). Libraries were sequenced using 90bp paired-end reads. The mean coverage achieved in target regions was 89x. Exome sequencing was performed using a hybrid capture of 193,094 exon targets from 18,862 coding genes.

Supplementary Note 4D. Virus captured sequencing

HPV fragments enrichment and sequencing were detected with the Viral Cap HPV kit (including 18 HPV types, such as HPV16, HPV18, HPV58, HPV31, HPV33, HPV35, HPV45, HPV52 etc, Mygeno Inc. China)[7]. The whole project process is divided into experimental stage and bioinformatics analysis. The experimental stage mainly includes the preparation of DNA whole genome library, capturing and sequencing of target gene region, and the next generation gene sequencing (Next500). The bioinformatics analysis mainly includes the basic bioinformatics analysis and disease gene information analysis.

Supplementary Note 4E. 10x long-range DNA library construction and sequencing

DNA was isolated using RecoverEase Genome DNA isolation kit (Agilent PN 720203) with slightly modification. After the molecular weight of isolated, DNA was assayed by pulsed field electrophoresis. For chromium library preparation, 1ng of isolated HMW DNA from each

sample was quantitated and denatured according to the manufacturer recommendations. Then the denatured DNA was spiked into the reaction master mix, and then mixed with gel bead and emulsification oil to generate droplets within a Chromium Genome chip. The rest part of library preparation was done following the manufacturer protocol (Chromium Genome v1, PN-120229). The barcoded libraries were sequenced on Illumina HiSeq Xten system. The BCL files were demultiplexed and converted to fastq files via bcl processor (v2.0.0).

Supplementary Note 4F. Sequencing data processing

As previously described[8], we followed the standard protocol of the Broad Institute to generate initial reads and qualities directly from the sequencer. Samples on the flowcells were sequenced with Illumina HiSeq 2500 using the V3 Sequencing Kits and the Illumina 1.3.4 pipeline. Reads were aligned to human genome build GRCh37 using a Burrows-Wheeler aligner[9]. Data from the Illumina HiSeq and X Ten were converted into BAM files for each sample, using Picard (Supplementary Tables 2-4, Supplementary Data 2 and 3).

Supplementary Note 5 | Analysis on VCS data

Totally, 150 FFPE SCCC samples were selected for HPV captured sequencing (VCS).HPV subtypes were found in all samples (Supplementary Table 2, Supplementary Data 4, Supplementary Figures 1 and 4). There are 35 samples (23.33%) reported two HPV subtypes, while others (115, 76.67%) harbor only one HPV subtype. HPV 18 was the most prevalent HPV subtype, which was positive in 125 samples (83.33%) and as the most abundant subtype (called as first subtype) in 113 samples (75.33%). The second most prevalent HPV subtype was HPV16, identified in 58 samples (38.67%) and as the first subtype in 37 samples (24.67%). Besides, two samples were HPV59 and HPV51 positive respectively, while both as the second HPV subtype. The individual HPV genomes were constructed as the process in WGS analysis mentioned below (Supplementary Note 6), note that the version of HPV16 reference was NC001526.4 for VCS data analysis, as NCBI nucleotide database updated recently. Constructed HPV genomes in all 150 SCCC FFPE samples (Supplementary Data 4) were uploaded to figshare (https://doi.org/10.6084/m9.figshare.19608618).

For HPV integration detection, all the first HPV subtypes were selected. While the second HPV subtypes should satisfy two requirements:

1. The average depth of the HPV genome was more than 5x;
2. The coverage of the HPV genome was more than 30%.

Totally, HPV18 was selected for virus integration detection in 123 samples (82%), and 55 samples (36.67%) to HPV16 (Supplementary Data 4). Credible HPV integrations were identified by FuseSV (in-house software) with the following filtration criterions:

1. Supported by at least five non-redundant junction-reads (i.e., split-reads);
2. At least 15bp sequences were covered by the junction-reads at both bilateral ends (i.e. human genome side and HPV genome side);
3. The sequence at the human side covered by the junction-reads should be uniquely mapped on the whole human genome (software: blat, v36x2).

Totally, 2,269 credible HPV integrations were identified in 81 samples (54%), among which 1436 cases (63.29%) were HPV18-related and 833 cases (36.71%) were HPV16-related (Supplementary Data 6). In average, each sample had about 28 HPV integrations. Eight samples had more than 100 HPV integrations. The sample T081 harbors the most credible integrations (count=381), while sixteen samples harbor only one credible integration. No HPV integration was detected in 69 samples, whose average depth of HPV subtypes' genomes was only 16.85x, much less than that (794.16x) of the 81 HPV-integrated samples (Mann-Whitney U Test, $P<0.001$, Supplementary Figure 5). The low-depth covered HPV genomes may be due to the DNA degradation of these FFPE samples, inducing lack of sufficient supporting reads for any virus integration.

As previous report, VCS technology has much higher captured efficiency than WGS[6]. Although lots of virus integrations could be detected from VCS data, the most of them were minor clonal, different from the major clones detected from WGS data. For one given sample, to obtain major clonal integrations, its HPV integration cases were sorted by the amount of their junction-reads numerically in descending order, and each integration was assigned the sorted index number, called JRstNO. According to the average amount of HPV integrations identified from WGS data in sixteen samples (4 cases per sample, Supplementary Data 8), we set the JRstNO as five, by which the major HPV integrations could be confirmed in each sample. Using this criterion, the whole credible HPV integrations (2,269) were divided into major type (326, 14.37%) and minor type (1943, 85.63%, Supplementary Data 6).

To annotate the major type of HPV integrations, we searched genes (Ensembl release 75) located in the flanking 750 kb distance from the integrated positions, as the sizes of chromatin loops range to such length in Hi-C study[10].Genes searched according to the major HPV integrations were found to enrich in five gene families (Supplementary Data7): the MYC family (*MYC*, *MYCL*, *MYCN*), the SOX family (*SOX2*, *SOX4*, *SOX7*, *SOX15*, *SOX17*), NR4A family (*NR4A2*, *NR4A3*), the ANKRD family (*ANKRD7*, *ANKRD13B*, *ANKRD17*, *ANKRD22*, *ANKRD34A*, *ANKRD35*, *ANKRD55*), and the *CEA* family (*CEACAM* gene-cluster at cytoband 19q13.2).The *MYC* family and the *NR4A* family were also detected in HPV integration analysis of WGS data. Besides, two gene sets, Sanger Cancer Census[11] and Cancer Dependence Map[12], were prevalently found around the major HPV integrated genomic loci, related to 72.84% (59/81) and 50.62% (41/81) SCCC FFPE samples, respectively.

As several hotspot genes, such as *MYC*, *MYCL*, *MYCN*, *SOX2*, *SOX9*, and *SOX10*, belong to Sanger Cancer Census or Cancer Dependence Map gene sets, we analyzed whether the other genes from these two gene sets were nearby the HPV integrations. Strict filtering strategy was applied: gene was filtered out from these two gene sets once it shares cytoband with the enriched gene families listed above. The remained genes were called as Cancer Census-others and Dependence Map-others, respectively. Surprisingly, 42 FFPE samples (51.85%) and 36 FFPE samples (44.44%) harbored major HPV integrations around genes of Cancer Census-others and Dependence Map-others, respectively. Moreover, gathering the five gene families and two gene sets (the xx-others), nearly half of SCCC FFPE samples (40/81, 49.38%) had HPV integrations nearby genes from at least two gene groups. Furthermore, counting in the *CEACAM* gene-cluster, the amount of such FFPE samples exceeds half (43/81, 53.09%). This finding means that more than half of SCCC FFPE samples have at least two cancer-related genes have the major type of HPV integrations nearby (Supplementary Data 7), and this finding may help to explain why SCCC is so exceptionally aggressive.

We can divide the FFPE samples into eight subtypes (Fig.1):

1) Integrations enriched in *MYC* gene family, sample percentage is 37.04% (30/81);
2) Integrations enriched in *SOX* gene family, sample percentage is 7.41% (6/81);
3) Integrations enriched in *NR4A* gene family, sample percentage is 6.17% (5/81);
4) Integrations enriched in *ANKRD* gene family, sample percentage is 4.94% (4/81);

5) Integrations enriched in *CEA* gene family (*CEACAM* gene cluster at 19q13.2), sample percentage is 3.70% (3/81);
6) Have integrations at Cancer Census genes, but not in previous gene families, sample percentage is 17.28% (14/81);
7) Have integrations at Cancer Dependence Map genes, but not in Cancer Census gene set and previous gene families, sample percentage is 9.88% (8/81);
8) Other samples that have HPV integrations at dispersed genomic locus, sample percentage is 13.58% (11/81).

The first five subtypes correspond to five gene families respectively, and displayed mutually exclusive distribution, e.g., in first subtype (*MYC* gene family), only 10% (3/30) samples emerged major HPV integrations in other subtypes (two in *SOX* gene family, one in *ANKRD* gene family). Samples in the eighth subtype had major HPV integrations at genomic loci previously reported to be associated with different types of cancers (Supplementary Data 7, Fig.1), and their HPV-integrated cytobands include T015 (2q36.3 (Ref.[13])), T016 (14q32.11 (Ref.[14])), T020 (3p24.1 (Ref.[15])), T090 (5q32 (Ref.[16])), T097 (8p22 (Ref.[17,18])), T118 (3q13.2 (Ref.[19])), T145 (17p11.2 (Ref[20]), 2p24.1 (Ref.[21]), 3p12.3 (Ref.[22]),3p26.2), T166 (3p22.1 (Ref.[23]), 5q12.1, 8p22, 9p21.1 (Ref.[24]), 9q22.1), T169 (13q21.31 (Ref.[25,7])), T172 (1q23.1 (Ref.[26,27])), T202 (5q15).The associations between these integrated genome loci and cancers imply that the major HPV integrations in samples of the eighth subtype may induce dysregulation of oncogenes or tumor-suppressors at the relevant genomic regions during SCCC carcinogenesis.

Supplementary Note 6| HPV variant assignment from WGS analysis

HPV variant was identified from WGS analysis. Reads were firstly aligned against human genome reference (hg19) via alignment tool (BWA-0.7.12). Unmapped reads and soft-clipped reads were extracted from alignment results. Then we aligned these reads to HPV reference database downloaded from NCBI nucleotide database. The database of reference HPV variants includes: HPV1 (NC001356.1), HPV11 (FR872717.1), HPV16 (NC001526.2), HPV18 (NC001357.1), HPV31 (J04353.1), HPV33 (M12732.1), HPV35 (M74117.1), HPV39 (M62849.1), HPV45 (KC470260.1), HPV52 (HQ537751.1), HPV56 (EF177181.1), HPV58 (D90400.1), HPV59 (X77858.1), HPV66 (U31794.1), HPV68a (DQ080079.1), HPV68b

(FR751039.1), HPV69 (AB027020.1), HPV6 (AF092932.1), HPV82 (AB027021.1), and HPV51 (KF436866.1). The HPV variant with most uniquely aligned reads and at least 20% coverage was selected as first HPV variant. The first HPV variant of each sample was consistent with the HPV genotyping result (Supplementary Data 5, sheet a). As the HPV variant of individual sample could have plenty of mutations diffed from HPV variant references downloaded online. We introduced iterative process to construct individual HPV genomes. In this process, we applied the detected mutations (SNV and InDel) to modify corresponding HPV variant genome. Next, we remapped the candidate reads again to this modified new HPV variant reference, and tested whether new mutations could be found out. If there are new mutations, viral genome modification will be done again, and reads will also be remapped to the newest modified HPV variant genome, till there is no new mutation could be identified any more. All mutations used to modify the HPV genomes were gathered in the final mutation list (Supplementary Figure 3; Supplementary Data 5, sheet b).

To compare the MS HPV genotyping and WGS detection, we tried to determine the secondary HPV variants in each sample. However, the signals of the second HPV variants were below the requirements, and then they were all filtered out. Based on the technical details of MS HPV genotyping in this project, we successfully found the existence of basic regions applied in the judgment of MS HPV identification, the GP6 region and the extensive region of HPV genomes, in the modified genomes of each sample (Supplementary Data 5, sheet a). Constructed individual HPV genomes of all samples were uploaded to figshare (https://doi.org/10.6084/m9.figshare.19608618).

Coverage along the HPV genomes shows consensus distribution with the viral integrations and HPV-related local haplotypes (Fig.2, Supplementary Figures 7-19), for example, in sample T002, viral genome region between integration break-points (i.e., +2453 and +3782) lacks reads coverage (Supplementary Figures 3 and 16). From this aspect, we could infer the free virus load level in samples, e.g., several samples carried no free virus (T001, T002, T005, T006, T008, T009, and T010), while the sample T003 might harbor large amount of free virus.

We applied genome walking PCR strategy to verify the individual HPV genomes. Sliding windows (800nt, overlap size = 100nt) of these modified genomes were designed to PCR experiments, the sanger sequences of PCR amplicons were assembled to get contigs. All

samples' individual HPV genomes were successfully verified as the contigs mapped the genomes well enough, covering most of constructed HPV genomes (Supplementary Data 5, sheet c). And, we also used PCR to obtain products spanning viral loop junction sites. One sample should be noted, which is sample T009. It lacks reads coverage in WGS data between its viral integration break-points, while the PCR successfully obtained this region, and continuous sequence were generated after sanger sequencing. This case infers sample T009 may harbor low level free virus or other integrations on its cancer genome, which is lower than the detection power of WGS under average depth about 50x.

Supplementary Note 7| HPV integration profiles from WGS analysis

Combining with constructed individual HPV genomes, we applied tool FuseSV (in-house software) to detected HPV integrations from WGS data of 16 SCCC tumors in a genome-wide perspective. FuseSV seeks two types of supporting reads, span-reads and junction-reads (i.e., split-reads), and generates putative junction library to obtain candidate integrations, similar to SOAPfuse[28]. Total of 55 HPV integrations were detected in 14 of the 16 SCCC samples (87.5%), and they were all successfully validated via sanger sequencing (Supplementary Data 8). FuseSV figures were uploaded to figshare (https://doi.org/10.6084/m9.figshare.19608618). Most integrations located in intergenic regions (74.5%, 41/55), and only 12 breakpoints were located on dysregulated cancer genes, including *INPP4B* (T002), *FGFR3* (T003), *NDUFV2* (T010), *ANKRD12*(T010), *MYC* (T011), and *NR4A2* (T019). In 14 samples with HPV integration, six (42.9%) were situated on or nearby genes in the *MYC* family, including *MYCN* and *MYC*, indicating tumorigenic functions of *MYC* family in cervical cancer development. Notably, integrations at *POU5F1B*, *MYC*, and *MYCN* were also shared by SqCC, adenocarcinoma cervical cancer (AdC) (Ref.[7]). Furthermore, similar to VCS data, HPV18 was the most frequent high-risk HPV subtype (46/55, 83.6%) integrated in SCCC while HPV16 was responsible for the rest integration events (9/55, 16.4%). Analysis of integration sites in HPV18 genome showed that integration hotspots were found to locate at *E1*, *E2* and *E4* genes. The expression analyses of integrated HPV DNA have shown that transcribed and coding regions of genes are frequently co-transcribed with HPV *E6* and *E7* oncogenes in 19 SCCC tumors (Supplementary Figure 2e). Break-points were prone to occur

in *E1* gene while *E6* / *E7* genes were over expressed (Supplementary Figure 2d, e).

Nearly 60% of the HPV integrations (31/55; *P*=0.007, Chi-square Test) found from WGS data harbored micro-homologous bases (MH) or small insertions at the junction (Supplementary Data 8 and 12), supporting an MH-mediated integration mechanism as our previous work suggested[7]. There are 21.8% of integrations fall into chromosome fragile region (common region and rare region), not significant comparing with expected (27.1%). And, we found viral integrated sites resided closer to DNase-I site than that of expected (*P*=0.012, Mann–Whitney U Test, Supplementary Data 12). The viral inserted sites adjacent DNase-I sites suggested that the open chromatin feature of the integrated genomic regions. There are 31 integrated break-points (56.3%) are within two nucleosomes (292nt) from DNase-I sites, but still not significant comparing with random distribution. Moreover, 19 integration sites were found located in repeated elements, including LTR, LINE, and SINE. However, comparing with random distribution, integration sites were not significantly enriched in these three types of repeated elements.

Supplementary Note 8| The verification of HPV integration positions

According to the supporting reads of the identified HPV integrations in SCCC samples, FuseSV automatically generated the bilateral sequences of each HPV integration case, with reads-covered bases in upper letters and extended bases (required length, 300nt) in low letters. Primers were generated by tool Primer3 (Ref.[29]). Primers were arranged in the reads-covered regions, which should support high-confidence. Sanger sequences were aligned by WEB BLAST. Only when the sequence (as query) was accurately mapped to the bilateral sequences (as subject database) in correct mapping orientation, it could be judged as successful validated case. All 55 HPV integration cases found in WGS data, and selected major HPV integrations from VCS data, were successfully validated by PCR and sanger sequencing (Supplementary Data 6 and 8, Supplementary Note 9).

Supplementary Note 9| PCR and Sanger sequencing verification.

Genomic DNA (10 ng) or 0.5ul of cDNA (reverse transcribed in a 20-µL reaction from 2ug total RNA) was amplified using the primer pairs shown in Supplementary Data 5,6,8, and 14. The presence of a single PCR product was verified by electrophoresis on a 1.5% agarose gel,

and 50 ng of PCR product was sequenced from both the 5' and 3' ends by Sanger sequencing using the original PCR primers.

These sequences were then aligned to the UCSC GRCh37/hg19 human reference by WEB BLAST (blast.ncbi.nlm.nih.gov). Colony picking was performed in ASE verification. PCR fragment (50ng) was ligated to pClone 007 Simple Vector and transformed into DH5α competent cells. Single clone was selected for sanger sequencing with the original primers. Sequences were aligned by Geneious (version R 9.1.6).

Supplementary Note 10| local haplotype construction from WGS analysis

Structural variants (SVs) and copy number alterations (CNAs) were detected by tools Meerkat[30] and Patchwork[31] from WGS data (Supplementary Data 20 and 24). We found the genomic regions nearby the HPV integrations showed extremely gain ratio (Supplementary Figure28b). Moreover, the edges of the duplicated genomic regions were very close to the break-points of SVs and HPV integrations. These CNAs and SVs relevant to the extremely duplicated genomic regions inferred the genomic instability adjacent to HPV integrated sites, consistent with previous reports[32,33]. Further analysis of the CNAs determined that the edges of these duplicated genomic regions were exactly the break-points of SVs and HPV integrations (Fig.2a and a-panels of Supplementary Figures 7-19).

Considering the linear structure of cancer genomic region flanking the HPV18 integration sites in the HeLa cell line has been well characterized[34], and the local genomic map of HPV integrations in several other cancer cell lines were also reported in details[35], we proposed the genomic regions around HPV integrations in tumor cells of our SCCC samples could also be solved, as we called it local haplotype.

Firstly, in each sample, the genomic region was divided into several segments (named by upper cases in Fig.2a and a-panels of Supplementary Figures 7-19) based on the break-points of SVs and HPV integrations, and the individual HPV genome was also partitioned into several segments (named by lower cases in Fig.2b and b-panels of Supplementary Figures 7-19) based on the viral break-points corresponding to the HPV integrations.

Secondly, from WGS data alignment results, we applied deepTools to correct the GC-bias in the local genomic region of HPV integrations (i.e., the genomic region in Fig.2a and a-panels

of Supplementary Figures 7-19). In tumor and control samples, the average depth of each segment was calculated from GC-bias corrected alignments using depth command of SamTools[36](v1.3) with minimum base and mapping quality set as 5 and 10, respectively. To convert the average depth from WGS data of tumor sample, we introduced purity and ploidy of tumor-cell in tumor sample calculated by tool Patchwork[31](Supplementary Data 2). Call the purity of tumor-cell in tumor sample as $\boldsymbol{Purity_T}$, the average ploidy of pure tumor-cell in tumor sample as $\boldsymbol{Ploidy_T}$, and the average ploidy of pure normal cell as $\boldsymbol{Ploidy_N}$ (assumed as 2). So the average ratio of the DNA from normal cells ($\boldsymbol{Ratio_N}$) in each genomic region was calculated as below:

$$\boldsymbol{Ratio_N} = 1 - \frac{\boldsymbol{Purity_T} * \boldsymbol{Ploidy_T}}{\boldsymbol{Purity_T} * \boldsymbol{Ploidy_T} + (1 - \boldsymbol{Purity_T}) * \boldsymbol{Ploidy_N}}$$

Then for each segment, we could also know its germline copy ratio from WGS data of control sample ($\boldsymbol{Copy\_ratio_N}$). So the depth of this segment from pure tumor-cell in tumor sample ($\boldsymbol{Detph_T}$) could be obtained from this segment's original average depth ($\boldsymbol{Detph_O}$) and whole genome average depth ($\boldsymbol{Detph_W}$) via formula below:

$$\boldsymbol{Detph_T} = \boldsymbol{Detph_O} - \boldsymbol{Detph_W} * \boldsymbol{Ratio_N} * \boldsymbol{Copy\_ratio_N}$$

Thirdly, from CNA results reported by Patchwork[31], we could know the total and minor copy number of chromosome arms and CNA segments. The most bilateral regions in local haplotype (call 5-prime segment as source, and 3-prime segment as sink in algorithms) were used to calculate the depth of single copy number (Supplementary Data 9). The copy times of HPV integrated or normal (as genome reference) local haplotype were determined manually according to the copy number result from Patchwork[31]. Then, the copy number of segments in local haplotypes could be determined (Fig.2a and a-panels of Supplementary Figures 7-19). Based on the segment connection relationship provided by viral integrations and SVs, we combined Integer Linear Programming with Weighted Oriented Eulerian Path algorithms (in-house software) to construct continuous segments path as single HPV integrated local haplotype with different copy times, which cost minimum changes on copy number of segments (Fig.2c and c-panels of Supplementary Figures 7-19; Supplementary Data 9). Each HPV-integrated local haplotype contains one or more contigs (we call it unit-cycle in our algorithm), which are the basic structure units that could move freely along the resolved local haplotype and even

could insert with each other. In the other words, the se-contigs are not phased with each other, i.e., their orders are not fixed in the resolved local haplotype. Besides, considering that local haplotypes in some samples have high copy number of repeated unit-cycles, such as T004 and T017, we do not rule out the possibility that double minutes might contribute to the duplications of these unit-cycles[37,30].So, the local haplotypes we solved in this project are just one of the possible solutions, and the result we show here is the one with simplest structure (call as Simplest Local Haplotype) which is easy to display (Fig.2c and c-panels of Supplementary Figures 7-19; Supplementary Data 9). Due to the short-reads in NGS data, we could not determine which one is genuine. We showed that long-range DNA sequencing technology may help: with 10x linked-reads, some local haplotypes with specific structures become more reliable than the others (Supplementary Note 13).

Most contigs in the HPV-integrated local haplotypes harbor one HPV inserted segment, which means the pairwise relationship among the HPV integration sites could be determined. We then investigated the feature combination of these pairwise integration sites (Supplementary Data 12). Comparing with the random distribution, the occurrence of pair-wise HPV integrations that either one or both have micro-homologies and small inner insertion was significant larger (77.4%, P=0.038, Chi-square Test). Similarly, the paired integrations enriched in the DNase-I site with less significance (P=0.065, Chi-square Test). Besides, in our data, the repeated elements showed no enrichment.

Supplementary Note 11| Haplotype Information of HPV Integrated Samples in SCCC

According to our knowledge, most of the reserved HPV-integrated local haplotypes of SCCC samples could be classified into three patterns, while several samples still not clearly characterized.

In the first pattern, several oncogenes' genetic regions were entirely duplicated by many times in the HPV-integrated local haplotypes, which may directly up-regulate the expression of these oncogenes. This pattern includes three SCCC samples: T008 (*MYCN*, Fig.2), T011 (*MYC*, Supplementary Figure 7), and T019 (*NR4A2*, Supplementary Figure 8).

In the second pattern, human endogenous gene fusions were formed due to the tandem duplication in the HPV-integrated local haplotypes, which might cause the up-regulated

expression of fusion partner genes. This pattern includes two SCCC samples: T003 (*FGFR3–TACC3*, Supplementary Figure 9), T010 (*ANKRD12–NDUFV2*, Supplementary Figures 10 and 30).

In the third category, the LCR region of HPV18 was inserted at the upstream of oncogene *MYC* (within 500kb), which may be activated by cis-regulation of the epithelium-specific viral enhancer in the LCR regions[38]. Furthermore, the inserted LCR regions were duplicated lots of times with the contigs, which may amplify the cis-regulation effects. Similarly, this cis-regulation of HPV18 inserted LCR was also proposed in HeLa cells[34]. This pattern includes four SCCC samples: T004, T014, T017, and T018 (Supplementary Figures 11-14). Note that *MYC* gene locus was also in broad amplifications in three samples (T004, T014, and T017), and this situation also happened in the HeLa cell line[34].

The patterns of the remained five samples are uncertain, as the influence induced by the HPV integrations are not clear enough. Samples T001, T005, T006, and T009 lack well-characterized genes in their HPV-integrated local haplotypes (Supplementary Figures 15, 17-19). In sample T002, the HPV18 integrated at the last intron of gene *INPP4B* (Supplementary Figure 16a), one tumor suppressor gene[39,40]. The last exon of *INPP4B* has high expression, clearly shown by the HPV-human fusion validated in sample T002 (Supplementary Figure 16c). But the other exons of *INPP4B* were expressed much lower than the last exon, inferring that the gene transcription might already be shut down at the promoter and the HPV-integration induced transcribed RNA of this gene might be truncated. Moreover, immunohistochemical staining in sample T002 showed low expression of protein encoded by *INPP4B* (Supplementary Figure 34c). So, in sample T002, for gene *INPP4B*, so far, we cannot determine whether its loss of function is related with HPV18-integration happened in the last intron.

We found aneuploidy of the chromosome that the HPV-integrated local haplotypes resided in several samples (Supplementary Data 9). In sample T003, the chromosome chr4 is haploid (copy number~1.0), so no reference-allele local haplotype (copy number~0) was constructed in its tumor cells (Supplementary Figure 9a, c). In sample T004, on the chromosome chr8, the upstream and downstream of HPV18-integration locus showed different copy numbers (upstream ~ 3.76, and downstream ~ 7.58). As we constructed the local haplotypes starting from the upstream, so four copies of local haplotypes were resolved (two copy are as reference,

another two copies are HPV18-integrated, Supplementary Figure 11a, c). Sample T005 also displays different copy numbers at the bilateral sides of HPV16 integration sites on the chromosome chr16: the upstream copy number is about three (~4.02), while the downstream is about four (~6.08). Local haplotype of sample T005 has four copies, including two HPV16-integrated and two reference alleles (Supplementary Figure 17a, c). Sample T006 harbors HPV18 integrations close to the centromere of chromosome chr3, whose short and long arms show imbalanced copy numbers: the short arm is about two copies (~2.10), and the long arm is about three copies (~3.38). The resolved local haplotype of sample T006 includes one copy of reference allele and one copy of HPV18-integrated allele (Supplementary Figure 18a, c). In sample T009, the chromosome chr15 is triploid (copy number ~ 3.0), similar to T019. Its local haplotype contains three copies, two of reference alleles and one of HPV18-integrated allele (Supplementary Figure 19a, c). In sample T014, the chromosome chr8 is tetraploid (copy number ~ 4.0), three copies of which were HPV18-integrated (Supplementary Figure 12a, c). In sample T017, the upstream of HPV18 integrated site on chromosome chr8 has copy number about three (~3.42), while the downstream is about four (~3.84, Supplementary Figure 13a, c), similar to sample T004. The resolved local haplotype of sample T017 was determined to have three copies, two of which are HPV18-integrated, and one is as reference. In sample T019, the chromosome chr2 is diploid (copy number ~ 2.0), one copy of which were determined to have HPV16-integrated local haplotypes (Supplementary Figure 8a, c).

HPV LCR region harbors the epithelium-specific viral enhancer, and showed important regulation functions in previous studies[38]. We then investigated the RNA expression of genes included in transcription factors (TFs) that have binding sites in the viral enhancer. These TFs included AP-1 (*FOS*, *JUN*, *JUNB*, and *JUND*), NFI (*NFIA*, *NFIB*, *NFIC*, and *NFIX*), Oct-1 (*POU2F1*), Tef-1 (*TEAD1*), Tef-2 (*KLF3*), and YY1 (*YY1*). We found that these genes were universally expressed in tumor samples (Supplementary Data 13), suggesting possible activation regulation of the viral enhancers. Genes related to TFs on the duplicated human genomic segments in HPV-integrated local haplotypes were also got examined. We found that numerous TF genes were expressed (FPKM > 10, Supplementary Data 13), inferring the duplicated human genomic region might have some regulation functions.

Supplementary Note 12| Preprocess and alignment of 10x long-range sequencing data

10x long-range linked-reads sequencing was applied on DNA of four SCCC samples (T011, T014, T017, and T018). High-quality data were generated by basic quality-control filtrations from raw reads (PE150, Supplementary Table 4, and Supplementary Note 4A). The official software from 10x technology, LongRanger (v2.1.2), was then applied to confirm the barcode sequence of each paired-end reads according to the base-qualities of 23 bases at the 5 primer of first end, which means the available sequence length of one paired-end reads is 127nt of the first end, and 150nt of the second end. Note that some paired-end reads got NA barcode as the low base-quality. As the alignment implemented in the LongRanger analysis pipeline automatically allows hard-clip, but not the soft-clip that commonly utilized in bioinformatics, we extracted the barcode sequence from the BX:Z: tag, and concatenated it with paired-end ID to mark the relationship. Then, we followed the traditional analysis pipeline (see Method) to align the barcoded-reads against human genome (hg19).

Based on barcoded-reads alignment result, we calculated the spanning size of each barcode by counting the mapped positions of its relevant barcoded-reads under the criterion that mapping quality is not zero, which could discard multiple mapped reads. Reads groups were formed from iterative searching for neighboring reads whose distance is less than 5kb on each single aligned chromosome. We required that each reads group must have more than five reads. The spanning size of one barcode is calculated as the distance of most bilateral aligned positions in its best reads groups, which has the most reads count. The barcode spanning size distributions have a cluster range from 10kb to 100kb (peak at ~30kb (T014) or ~60kb (T011, T017, T018)) on each available chromosome in all samples (Supplementary Figures 22-25).

Supplementary Note 13| Linkage among the breakages in local haplotypes

From the barcoded-reads alignment, we constructed the individual HPV genome of four SCCC samples. All HPV integrations and rearrangements in local haplotypes of these four samples were successfully recalled (Supplementary Data 10). Barcodes related to each breakage (SV or HPV integration) in local haplotypes were collected from junction reads. Then, for each pair of breakages, their shared barcodes were counted as the linkage, one definition we introduced for next correlation calculation.

To calculate the correlation between linked-reads sequencing data and the local haplotypes we proposed in these four samples, we introduced two definitions: the first one is the anchors in one given local haplotype; and the second one is the linkage of pair-wise of anchors (called as PWanchor).

One anchor is just one position selected in the local haplotype. Two kinds of anchors were introduced: JUNC-anchor and POS-anchor. JUNC-anchor is the junction site of SVs and HPV integrations on the local haplotype (e.g., junction aE in Fig.2c). POS-anchor is the middle-point of the host segments in the local haplotype (e.g., segments A~K in Fig.2c). The distance between any two anchors (i.e., PWanchor) can represent the structure of relevant local haplotype.

Linkage of one PWanchor is the counts of 10x-barcodes shared by the relevant two *anchors*. In this work, we only considered pair-wise of same type anchors, i.e., JUNC-vs-JUNC or POS-vs-POS. From the 10x long-range sequencing data, we could get the barcode list of each anchor (Supplementary Data 11), then calculate the observed linkages. And, for any given local haplotype resolved from our algorithms, we simulated 10x long-range sequencing with mean-depth as 50x.Then, we could also obtain the proposed linkages of the given local haplotype.

For each sample, we let the algorithms randomly construct valid local haplotypes in 100,000 times. For each resolved local haplotype, the Pearson ratio between observed linkages and proposed linkages was calculated. The local haplotype with highest Pearson ratio was selected as the best one theoretically (called as Random-Best Local Haplotype). The ratio is high enough to tell that the 10x linked-reads sequencing data supports and optimizes the resolved local haplotype of these four SCCC samples (Simplest Local Haplotype is 0.61-0.97, Random-Best Local Haplotype is 0.90-0.99, Supplementary Figure 26, Supplementary Data10 and 11). The Random-Best Local Haplotype of these four SCCC samples were shown in Supplementary Figure 21. This work implies that, comparing with the traditional WGS data, 10x long-range linked-reads sequencing data could help the local haplotype analysis of viral integrations to be more accurate and precise.

Supplementary Note 14| The verification of virus-human fusion genes

HPV-human fusion events were identified from RNA-seq data by tool FuseSV. Totally, we determined 83 HPV-human fusions classified in three categories (Supplementary Data 14). First category includes 32 fusion transcripts whose junction positions are consistent with the HPV integration sites. These fusions depicted the direct expression of virus integrations. Second, 40 fusions were processed by RNA splicing at the canonical splicing site, GU-AG. Moreover, the upstream partners of these spliced fusions were all HPV sequences, suggesting that transcription may initiate from inserted virus genome in the haplotypes, for example, the LCR regions or viral promoters. From these spliced fusions, three 5-primer splicing hotspots (+233, +929, and +1357) were found on HPV18 genome, and conformed to the canonical donor splicing motif (agGUa). In the third category, 11 fusions maybe from HPV integrations missed by WGS but were signal-amplified via the transcription process, confirming that RNA-seq could compensate for the insufficient detection power of WGS when dealing with low-frequency variations. For the third category fusions, validation work needs to operate on both cDNA and DNA level. To generate the primers for PCR experiment, similar work was done on HPV-human fusions as HPV integrations (Supplementary Note 8).

In total, 91.6% of HPV-human fusion cases were successfully validated (76/83, Supplementary Data 14 and Supplementary Note 14). The validated HPV-human fusions supported the expression activity of the HPV integrated genomic regions.

Supplementary Note 15 | The verification of ASEs

To further determine whether the expression activities of the HPV integrated genomic regions are mainly from the HPV-integrated local haplotypes, we identified the allele specific expressions (ASEs) located in the resolved local haplotypes. Totally, 204 ASEs in the local haplotype regions were detected, among which 202 sites were in the dbSNP database (Build-148, Supplementary Data 15). All 204 identified ASEs could be classified into two categories, first is reasoned by loss-of-heterozygosity (LOH, 23.0%, 47/204), and second is heterozygous sites selectively expressed (shorten as Het, 77.0%, 157/204). For one given position, we required that the minimum reads supporting one allele should be four, at least two from forward and reversed mapped respectively. In control samples' WGS data, both alleles should meet this criterion. In tumor samples' WGS data, for Het type, reads supports of both alleles should be

larger than four respectively, while for LOH type, one allele's reads support should be smaller than or equal to four. In tumor samples' cDNA, apparent difference was required between reference and alteration alleles, the difference between two alleles' supporting-reads number must larger than or equal to the 75% of the maximum one. We selected 50 ASEs (7 LOH and 43 Het, Supplementary Data 15) from genetic regions for validation, such as *MYC*, *MYCN*, *NR4A2*, *FGFR3*, and *TACC3* and so on.

Four Het ASE sites (rs57907091, rs4669017, rs11774777, and rs11781774) showed shifted allele frequency only in control sample DNA, but their tumor DNA and tumor cDNA accorded with expected (from NGS data), still supporting the allele specific expression activities. Another two Het ASE sites (rs3891248 and rs4645948) showed biased allele frequency in tumor cDNA, but the frequency shift of the later is acceptable as it still showed apparent specific expressions. All of the other ASEs (7 LOH, and 37 Het) showed clear consistence with expected (from NGS data). So, the successful validation rate of ASEs is 98% (49/50).

According to the alleles' frequency, the overexpressed ASE alleles were all phased in the HPV-integrated local haplotypes. For instance, in WGS data of tumor sample T008, at the rs3755135 locus in gene *MYCN* (Fig.2e), the reference allele (T, 23 reads supported) is much less than alteration allele (C, 710 reads supported). As rs3755135 located in the human segments M region, which is highly amplified in the HPV-integrated local haplotype (copy number is about 36, Allele 2nd in Fig.2c), so the alteration allele (C) must reside on the HPV-integrated local haplotype. Furthermore, in the RNA-seq data of tumor sample T008, at rs3755135, the alteration allele (C) is the overexpressed ASE allele (41 reads supported), while the reference allele (T) has no reads supporting. So, ASE at rs3755135 showed the specific activation of the allele that has HPV18 integrated (Allele 2nd in Fig.2c).

Supplementary Note 16| Linkage of ASEs and local haplotypes

Barcoded-reads were extracted from 10x data alignment result at the positions of ASEs that found from the traditional WGS data and RNA-seq data. Of each read, the 10x-barcode, the alleles it supports, and its mapped-orientation were determined based on the read-id, SAM-flag, and read sequence (Supplementary Data 16 and 17). Comparing with the barcodes list of each breakage in local haplotype (Supplementary Data 11), the number of shared barcodes

between one given ASE and one given breakage was counted. Almost all shared barcodes supported the activated alleles (i.e., the specifically expressed alleles) phasing with the local haplotype, as all the imbalance frequencies were close to 100% (83 available ASEs, average imbalance frequency is 98.87%, Supplementary Data 16).

Supplementary Note 17| Deciphering mutational SV signatures in SCCC

We applied computational framework proposed by Alexandrov[41] to decipher mutational signatures from WGS of 16 SCCCs. DNA mutations in cancer genomes are caused by complicated interplay between DNA damage and defective repair machinery. Exogenous and endogenous mutagenic factors can cause lesions (such as point mutation, insertion and deletion, translocation, inversion and copy number alteration) on tumor genome over the course of cancer formation. Exogenous factors like ultra-violet exposure and smoking can cause C>A/G>T and C>T/G>A at dinucleotide, respectively. Chemotherapeutic agents such as platinum-based drugs can induce DNA cross-linking and bulky adducts. Other chemical agents including benzopyrene and aflatoxin (contained in waste oil) are mutagenic. DNA double-strand break (DSB) caused by ionizing radiation poses great threat to genome stability; this process further creates more single strand DNA molecules, which are vulnerable to endogenous and exogenous attacks. DNA repair of DSB is error-prone in which new types of mutations may be introduced during this process. Endogenous factors include spontaneous deamination in CpG island (mutated base underlined), deamination via APOBEC mediation, oxidative substances produced by metabolism, transcription-coupled damage (TCD) on the untranscribed DNA strand[42]and polymerase error during laborious DNA replication. Mutational signature is defined as process of DNA damage and repair that leads to single-nucleotide substitutions, short insertions and deletions, structural variations and copy number change.

The final somatic mutation dataset is mixture of mutations generated by multiple distinct endogenous and exogenous mutational processes, consisted of two to eight driver gene mutations and a large fraction of passenger mutations. Although passenger mutations do not have direct or indirect consequence upon tumorigenesis, they carried information about environmental factors exposed to cells over the course of cancer development. Some mutational processes are operative earlier in the lifetime whereas the others appear later in a specific time

course.

Single-nucleotide substitutions are conventionally divided into 6 categories by collapsing strand information resulting in C:G>A:C, C:G>G:C, C:G>T:C, T:A>A:T, T:A>C:G and T:A>G:C mutations. When the 5' and 3' bases are taken into account, the number of mutational categories becomes 96 (i.e. 96=4×6×4). Theoretically, we can consider more than one 5' and 3' flanking bases. The total numbers of mutational categories depend upon the number of flanking base. For instance, if we set flanking bases number to 2, the number of categories becomes 1,536 (4×4×6×4×4), which significantly increase the computational time, variabilities and difficulties in interpreting the outputs. Alexandrov and colleagues have demonstrated that less reproducible mutational signatures if considered two flanking bases[41]mutational signature is defined over a finite mutational space **Ω** with n categories. Mathematically, a mutational signature W_1 is modeled as probability distribution $W_1 = [w_1^1, w_1^2, w_1^3, \ldots, w_1^n]^T$, where w_1^i represents the probability of mutational process W_1 that causes mutation at the i-th category in mutational space **Ω**. Given that w_1^i is probability distribution defined in **Ω**, it follows:

$$\sum_{i=1}^{n} w_1^i = 1, whereas\ w_1^i \geq 0\ for\ i = 1, 2, 3, \ldots, n$$

Mutational process W_1 can be operative in many different cancer genomes with varying contribution to each genome. Each mutational process has unique distribution over 96 mutational categories.

Let h_g^i denote mutational exposure in cancer genome g that is caused by K mutational processes defined by $\boldsymbol{W}$ at the i-th mutational category, and a_g^i denote the observed number of somatic mutations in the i-th mutational category of cancer genome g. Mathematically, a_g^i can be formulated as:

$$a_g^i \approx \sum_{j=0}^{K} w_j^i\, h_g^j$$

The above formula can be easily extended to matrix multiplication form for an arbitrary number of cancer genomes:

$$\boldsymbol{A} \approx \boldsymbol{W} \times \boldsymbol{H}$$

The purpose of mutational signature analysis is to depict characteristic mutational patterns representing different mutagenic processes. This issue is analogous to blind source separation.

Suppose in a cocktail party, many people are speaking simultaneously and we have placed multiple microphones at different corners to record their conversations. Each microphone records mixture sounds of different people. The purpose is to extract their conversations and loudness from the audio recordings. The well-established algorithm in this area is nonnegative matrix factorization (NMF). The problem of mutational signature extraction intrinsically fulfills the non-negativity assumption of NMF. The NMF has been successfully applied to extract mutational processes and their exposures from multiple types of human cancer.

The NMF begins with factorizing mutation matrix $\boldsymbol{A}$ into two nonnegative matrices $\boldsymbol{W}$ and $\boldsymbol{H}$ (i.e. $\boldsymbol{A} \approx \boldsymbol{W}*\boldsymbol{H}$); where $\boldsymbol{A}_j^i$ is the number of mutations belonged to mutational category *i* in sample *j*. The mutational processes are represented by matrix $\boldsymbol{W}$. Each column of $\boldsymbol{W}$ represents the characteristic patterns of different mutational processes. Rows of $\boldsymbol{H}$ quantifies the number of somatic mutations generated by every mutational process, known as metagene expression. In practice, firstly (step 1) mutations in matrix $\boldsymbol{A}$ accounting for less than or equal to 1% are removed, resulting in a reduced mutation matrix $\boldsymbol{A}_r$. Secondly (step 2), bootstrap resampling was used to generate a new matrix $\breve{\boldsymbol{A}}_r$ from $\boldsymbol{A}_r$. The probability to sample mutations at the *i*-th category for cancer genome *g* is proportional to $\boldsymbol{A}_r^i / \sum_{i=0}^{m} \boldsymbol{A}_r^i$ (where *m* is the x-dimension of $\boldsymbol{A}_r$), thus the total number of mutations (column sum) for each cancer genome in $\breve{\boldsymbol{A}}_r$ are equal to $\boldsymbol{A}_r$. Thirdly (step 3), for each NMF rank (i.e. mutational signature number) *k=1…g*, $\breve{\boldsymbol{A}}_r$ was factorized into $\breve{\boldsymbol{W}}_r$ and $\breve{\boldsymbol{H}}_r$ by minimizing Frobenius error $||\breve{\boldsymbol{A}}_r - \breve{\boldsymbol{W}}_r \times \breve{\boldsymbol{H}}_r||_F^2$. After finishing step 3, two groups of matrices for each NMF rank *k* were obtained, here denoted as S_W and S_H. Cosine similarity computed from S_W is used as a measurement of reproducibility of mutational signature. The reconstruction error defined as $||\breve{\boldsymbol{A}}_r - \breve{\boldsymbol{W}}_{r\_average} \times \breve{\boldsymbol{H}}_{r\_average}||_F^2$ was also calculated; whereas $\breve{\boldsymbol{W}}_{r\_average}$ and $\breve{\boldsymbol{H}}_{r\_average}$ are average matrices computed from S_W and S_H, respectively. An optimal NMF rank is selected based on high reproducibility and low reconstruction error.

The prevalence of host genetic mutations was shown in Supplementary Figure 27, and Supplementary Data18 and 19, exhibiting an average mutation rate of 1.3 non-silent mutations per million base pairs (Mb). The inactivated mutations of the tumor suppressor genes*TP53* and *RB1* were detected at a frequency of only 4.3% in SCCC samples. Fifteen other genes that are consistently reported in other human cancer types were often detected in SCCC, including

MLL3 (13.0%), which is mutated in several malignancies[43] (*ATRX* (8.7%), which was identified in pancreatic neuroendocrine cancer; *PIK3CA* (8.7%) and *FBXW7* (4.3%), which are mutated in SqCC[44](14% and 15%, respectively); as well as *KRAS* mutations (G12D and G12V, 8.7%) in SCCC[45](Supplementary Figure 27a).

Furthermore, five mutational signatures (Signatures 1–5) were extracted (Figure 4a, Supplementary Figure 27b-o and Supplementary Note 17), each contributed a different proportion of mutations to the regular- and hyper-mutated groups. Signature 1 was featured by a C>A mutation at TpCpW (where W = A or T, with the mutated base underlined) and a T>G mutation at TpTpT, whereas Signature 2 was characterized by a C>T mutation (Figure 4a). The hyper-mutated sample T007 was exclusively over-represented with mutations attributable to Signature 1, which accounted for 98.3% of all mutations. This sample harbored missense mutations of *POLE* located at the DNA polymerase exonuclease domain, suggesting a defect in DNA proofreading (Supplementary Figure 27c). The other hyper-mutated sample T025 was featured by a dominant mutation attributed to Signature 2 (87.3%), which has been reported to be associated with defective DNA mismatch repair (MMR). Signature 3 was also known as the APOBEC signature (i.e., dominated by C>G and C>T mutations at TpCpW) and was widespread among the 14regular-mutated SCCC samples (Fig.4a). However, the etiology implicated in Signatures 4 and 5 were still obscure. *POLE*- and MMR-related mutational Signatures 1 and 2 in the hyper-mutated SCCC samples were dramatically associated with replicative mutational asymmetry (P=0.00015 and P=0.014, respectively; Supplementary Figure 27c, j), which might suggest a carcinogenesis mechanism for hyper-mutated samples without HPV integration. By contrast, the APOBEC mutation signature 3, widespread across human cancers, was ubiquitous among the regular-mutated SCCC samples and associated with transcriptional biased mutational asymmetry (P=0.036; Supplementary Figure 27c, i). When compared with mutation signatures obtained from previous SCLC studies[46-48]downloaded from cBioPortal[49], we observed that SCCC is characterized by distinct mutation signatures except for APOBEC and C to T at CpG signatures (Supplementary Figure 27n, o).

In all 1,792 high-confidence somatic SVs were identified from the 16 SCCC genomes, ranging from 72 to 149 per genome with an average of 112. The frequency of different types of somatic SVs in each sample was shown (Supplementary Figures 28c-e, 30, 33, and Supplementary Data 26 and 27). SV Signatures 1 and 2, featured by alt-EJ, FoSTeS and NHEJ, were extracted. SV Signature

1 has higher contribution from alt-EJ, whereas SV Signature 2 is less represented by alt-EJ but with high proportion of FoSTeS and NHEJ (Supplementary Figure 28c-e).

Supplementary Note 18| OncoScan CNV FFPE assay

A total of 132 FFPE tumor samples were performed using Affymetrix OncoScan® CNV FFPE Assay Kit (Affymetrix, Santa Clara, CA, US), a whole-genome copy number assay, according to the Affymetrix OncoScan™ Assay User Manual (Affymetrix, Santa Clara, CA, US; Fig. 4d, e, Supplementary Figure 28a). Briefly, DNA was extracted from FFPE tissues and normalized to a concentration of 12 ng/ul. The annealed DNA samples were processed through the Pre-PCR and Pos-PCR before Array hybridization. After wash and and Stain in Gene Chip Fluidics Station, array was Scan in Gene Chip Scanner 3000. Raw data of CEL files passed quality control were available for data analysis by Affymetrix OconScan® Console v1.3 (Affymetrix, Santa Clara, CA, US) to generate OSCHP files. The data were further analyzed by Chromosome Analysis Suite (ChAS) software to detect copy number change. The data was also analyzed with Nexus Copy Number Version3 (standard edition, BioDiscovery, Inc. 2014). We used DNA copy to perform copy number segmentation and GISTIC2.0 to identify significantly amplification and deletion peaks as previously described[50].

Supplementary Note 19| Signaling pathways and survival analyses

The comprehensive analysis of the hotspot mutations, CNA, differential expressed genes (DEG), fusion genes and HPV integration was considered to gain insights into signaling pathways. Firstly, two distinct modules were found by performing STRING protein interaction network analysis on the top 100 DEG over 5-fold expression levels in RNA-seq data (Supplementary Figure 31 and Supplementary Data 24). The major module consists of cell cycle regulators converged on *PLK1* (fc=9.52 and *P*=1.11E-13) centralized cell cycle genes, whereas the other encompassed genes closely related to neuroendocrine differentiation (NED), such as *INSM1*and *ASCL1* (fc=45.45 and *P*=7.85E-11 for *INSM1*; fc=14.82 and *P*=9.15E-05 for *ASCL1*, respectively). Consistent with STRING results, gene set enrichment analysis (GSEA) also revealed significantly increased expression of signaling pathways including cell cycle pathway, *MYC* family and HPV 18 viral genes (Supplementary Figure 32, Supplementary

Data 25, and Supplementary Table 5). In cell cycle, the inactivated mutations of tumor suppressor genes *TP53* and *RB* were detected by only 4.3%, which significantly differ to those of SCLC[46]. The specific mutations of *RAS-RAF* key genes at *KRAS* (8.7%), *BRAF* (8.7%), and *HRAS* (4.3%) were also examined (Supplementary Figure 27a). Moreover, the focal amplification harbored *MYC* family genes (*MYC*, *MYCN* and *MYCL1*) and *CCNE1* were found, so was *FHIT* deletion (Fig.4b-e). In addition, *FGFR3-TACC3* fusion was associated with HPV integration in sample T003, resulting in the up-regulated expression of *FGFR3* on both mRNA and protein levels (Supplementary Figures 9a, d, Supplementary Data 27). *MYC* family, as the internal links between cell cycle and neuroendocrine differentiation, were found to be HPV integration hot spots (near *MYC* 35.7%, *MYCN* 7.1%). Moreover, the 5-year OS and DFS rates of SCCC patients with positive MYC, *INSM1* stains were decreased obviously than those in negative ones (P=0.003 and P=0.005 for OS; P=0.004 and P=0.003 for DFS; Log-rank test; Supplementary Figure 34b,n-q).There were no significant differences in the 5-year OS and DFS rates between positive and negative groups for the four canonical neuroendocrine markers (P > 0.05, respectively; Log-rank test; Supplementary Figure 34f-m).

Supplementary Note 20| Immunohistochemical staining

The immunohistochemical staining was detected with 4 μm FFPE sections according to the manual immunohistochemistry staining methods as previously studies[51,52]. We used rabbit anti-MYC (cat#ZA-0555; dilution:1:100; ZSGB-BIO; CHINA), mouse anti-ASCL1 (cat#556604; dilution:1:100, BD, USA), mouse anti-INSM1 (cat#sc-271408; dilution:1:100; Santa Cruz; USA), rabbit anti-CHGA (cat#ab283265; dilution:1:100; Abcam; USA), rabbit anti-NCAM1 (cat#ab220360; dilution:1:100; Abcam; USA), rabbit anti-SYP (cat#17785-1-AP; dilution:1:50; Proteintech; USA), rabbit anti-ENO2 (cat#10149-1-AP; dilution:1:50; Proteintech; USA), rabbit anti-INPP4B (cat#ab81269; dilution:1:50; abcam; USA), rabbit anti-MYCN (cat#10159-2-AP; dilution:1:100; Proteintech; USA), mouse anti-FGFR1 (cat# ab829; dilution:1:100; Abcam; USA), rabbit anti-Notch1 (cat#ab52627; dilution:1:100; Abcam; USA), rabbit anti-IDH2(cat# ab131263; dilution:1:100; Abcam; USA), mouse anti-ERBB 4 (cat# ab219208; dilution:1:100; Abcam; USA), rabbit anti- GATA1 (cat# ab28839; dilution:1:100; Abcam; USA), rabbit anti-NR4A3 (cat#ab188752; dilution: 1: 500, Abcam; USA), rabbit anti-

Jun (cat#ab40766; dilution:1:200, Abcam; USA), rabbit anti-ROS1 (cat#ab189925; dilution:1:300; Abcam; USA), rabbit anti-ERG (cat#ab92513; dilution: 1: 500; Abcam; USA), rabbit anti-SOX17 (cat#ab224637; dilution:1:100; Abcam; USA), mouse anti-MAPK1 (cat#sc-271269; dilution:1:50; Santa Cruz; USA), rabbit anti-SOX4 (cat#bs-11208R; dilution:1:50; BIOSS; CHINA), rat anti-MYCL (cat#bs-24627R; dilution:1:400; BIOSS; CHINA), rabbit anti-STAG2 (cat#19837-1-AP; dilution:1:50; Proteintech; USA), rabbit anti-SOX2 (cat#11064-1-AP; dilution:1:50; Proteintech; USA), rabbit anti-SOX15 (cat#16725-1-AP; dilution:1:50; Proteintech; USA), rabbit anti-Nurr1/NR4A2 (cat#10975-2-AP; dilution:1:100; Proteintech; USA). Diaminobenzidine was used to detect antibody. Images were photographed using cellSens Dimension (version 1.8.1, Olympus). Because of tissue section and antibody deficiency, ANKRD gene family (ANKRD22, ANKRD35, ANKRD55), CEACAM gene family (CEACAM21, CEACAM6, CEACAM5, CEACAM3) and Cancer Dependency Map-others was not detected the immunohistochemical staining.

Diaminobenzidine was used to detect antibody. Images were photographed using cellSens Dimension (version 1.8.1, Olympus). The immunohistochemical score of each sample was measured based on staining intensity and percentage of the cells stained. The staining intensity was sub-classified a rating from 0 to 3: 0 = negative; 1 = weakly positive; 2 = moderately positive; and 3 = strongly positive. The percentage of positive cells was quantitated in three random fields and graded in five categories: 0 = 0–9%; 1 = 10–24%; 2 = 25–49%; 3 = 50–74% and 4 = 75–100% of cells positively stained. The intensity score and percentage score were multiplied to yield the immunoreactivity score for each specimen[51,52]. Each lesion was separately examined and scored by two pathologists. The cases with discrepant scores were discussed to obtain a consensus. Information of the samples subjected to IHC staining is summarized in detail in Supplementary Data 1 and Supplementary Tables 7-9.

Supplementary Note 21| The 5-year OS and DFS rates of SCCC patients whose HPV integration sites were located on genes or gene families than those without HPV integration.

In addition to *MYC* family genes (37.9%), we identified *SOX* family (8.4%), *NR4A* family (6.3%), *ANKRD* family (7.4%), and *CEA* family (3.2%) genes as novel HPV-integrated hotspots in SCCC.

Firstly, we calculated the 5-year OS and DFS rates of SCCC patients whose HPV integration sites were located on genes or gene families, such as *MYC* gene family, *SOX* gene family,*NR4A*gene family, *ANKRD* gene family, *CEA* gene family, Cancer Census genes, Cancer Dependence Map genes and *MYC* gene, comparing with those without HPV integration (*P*=0.4856, *P*=0.1706, *P*=0.1827*, P*=0.1031*, P*=0.5332, *P*=0.4906, *P*=0.2311*, P=0.5453* for OS; *P*= 0.4588, *P*=0.0705*, P=0.0029, P=0.1471, P=0.5608, P=0.2801, P=0.8716, P=0.6132* for DFS). There were no significant differences in the 5-year OS and DFS rates between integration groups and non-integration groups for the *MYC* gene family, *SOX* gene family, *ANKRD* gene family, *CEA* gene family, Cancer Census genes, Cancer Dependence Map genes and *MYC* gene ($P > 0.05$, respectively; Log-rank test), except for*NR4A* gene family (The *P* values of the corresponding 5-year OS and DFS were 0.0093and 0.0029, respectively; Log-rank test; Supplementary Tables 7 and 8). Generally, HPV integration is closely associated with tumorigenesis but not the long-term outcome rates in SCCC.

Moreover, the immunohistochemical staining was detected with 4 μm FFPE sections for protein expressions in genes or gene families with HPV-integrated hotspots in order to research the functional expression with viral integration (Supplementary Note 20). There were no significant differences in the immunoreactivity score between integration groups and non-integration groups for the *MYC* gene family, *SOX* gene family, *NR4A* gene family, Cancer Census genes ($P > 0.05$, respectively; Mann-Whitney U test; Supplementary Table 9).

Supplementary Reference:

1. Atienza-Amores M, Guerini-Rocco E, Soslow RA, Park KJ, Weigelt B. Small cell carcinoma of the gynecologic tract: A multifaceted spectrum of lesions. *GynecolOncol* 134, 410-418 (2014).
2. Viswanathan AN, Deavers MT, Jhingran A, Ramirez PT, Levenback C, Eifel PJ. Small cell neuroendocrine carcinoma of the cervix: outcome and patterns of recurrence. *GynecolOncol* 93, 27-33 (2004).

3. Zivanovic O*, et* al. Small cell neuroendocrine carcinoma of the cervix: Analysis of outcome, recurrence pattern and the impact of platinum-based combination chemotherapy. *GynecolOncol* 112, 590-593 (2009).
4. Shi YY*, et al.* A genome-wide association study identifies two new cervical cancer susceptibility loci at 4q12 and 17q12. *Nat Genet* 45, 918-U262 (2013).
5. Li S*, et al.* Development and validation of a surgical-pathologic staging and scoring system for cervical cancer. *Oncotarget* 7, 21054-21063 (2016).
6. Yi X*, et al.* A New PCR-Based Mass Spectrometry System for High-Risk HPV, Part I Methods. *Am J ClinPathol* 136, 913-919 (2011).
7. Hu, Z. *et al.* Genome-wide profiling of HPV integration in cervical cancer identifies clustered genomic hot spots and a potential microhomology-mediated integration mechanism. *Nat Genet*47, 158-163 (2015).
8. McKenna A*, et al.* The Genome Analysis Toolkit: A MapReduce framework for analyzing next-generation DNA sequencing data. *Genome Res* 20, 1297-1303 (2010).
9. Li H, Durbin R. Fast and accurate long-read alignment with Burrows-Wheeler transform. *Bioinformatics* 26, 589-595 (2010).
10. Rao SSP*, et al.* A 3D Map of the Human Genome at Kilobase Resolution Reveals Principles of Chromatin Looping. *Cell* 159, 1665-1680 (2014).
11. Futreal PA*, et al.* A *census* of human cancer genes. *Nat Rev Cancer* 4, 177-183 (2004).
12. Tsherniak A*, et al.* Defining a Cancer Dependency Map. *Cell* 170, 564-+ (2017).
13. Li J*, et al.* 2q36.3 is *associated* with prognosis for oestrogen receptor-negative breast cancer patients treated with chemotherapy. *Nat Commun* 5, (2014).
14. Pekarsky Y, Hallas C, Isobe M, Russo G, Croce CM. Abnormalities at 14q32.1 in T cell malignancies involve two oncogenes. *P NatlAcadSci USA* 96, 2949-2951 (1999).
15. Drabkin H*, et al.* Localization of human ERBA2 to the 3p22----3p24.1 region of chromosome 3 and variable deletion in small cell lung cancer. *ProcNatlAcadSci U S A* 85, 9258-9262 (1988).
16. Shu X*, et al.* Genetic variants of the Wnt signaling pathway as predictors of aggressivedisease and reclassification in men with early stage prostate cancer on active surveillance. *Carcinogenesis* 37, 965-971 (2016).

17. Matsuyama H*, et al.* Deletions on chromosome 8p22 may predict disease progression as well as pathological staging in prostate cancer. *Clin Cancer Res* 7, 3139-3143 (2001).
18. Tsuneizumi M*, et al.* Association of allelic loss at 8p22 with poor prognosis among breast cancer cases treated with high-dose adjuvant chemotherapy. *Cancer Lett* 180, 75-82 (2002).
19. Dennis TR, Stock AD. A molecular cytogenetic study of chromosome 3 rearrangements in small cell lung cancer: Consistent involvement of chromosome band 3q13.2. *Cancer Genet Cytogen* 113, 134-140 (1999).
20. Both J, Wu T, Bras J, Schaap GR, Baas F, Hulsebos TJM. Identification of Novel Candidate Oncogenes in Chromosome Region 17p11.2-p12 in Human Osteosarcoma. *Plos One* 7, (2012).
21. Stevens KN, Vachon CM, Couch FJ. Genetic Susceptibility to Triple-Negative Breast Cancer. *Cancer Res* 73, 2025-2030 (2013).
22. Cody NAL*, et al.* Characterization of the 3p12.3-pcen Region Associated With Tumor Suppression in a Novel Ovarian Cancer Cell Line Model Genetically Modified by Chromosome 3 Fragment Transfer. *Mol Carcinogen* 48, 1077-1092 (2009).
23. Sai Y*, et al.* 3p22.1 and 10q22.3 deletions detected by fluorescence in situ hybridization (FISH) - A potential new tool for early detection of non-small cell lung cancer (NSCLC). *J ThoracOncol* 3, 979-984 (2008).
24. Sasaki S*, et al.* Molecular processes of chromosome 9p21 deletions in human cancers. *Oncogene* 22, 3792-3798 (2003).
25. Chen CS*, et al.*Defining a common region of deletion at 13q21 in human cancers. *Gene Chromosome Canc* 31, 333-344 (2001).
26. Viapiano MS, Bi WL, Piepmeier J, Hockfield S, Matthews RT. Novel tumor-specific isoforms of BEHAB/Brevican identified in human malignant gliomas. *Cancer Res* 65, 6726-6733 (2005).
27. Lipska BS*, et al.* c.1810C > T Polymorphism of NTRK1 Gene is associated with reduced Survival in Neuroblastoma Patients. *Bmc Cancer* 9, (2009).
28. Jia WL*, et al.*SOAPfuse: an algorithm for identifying fusion transcripts from paired-end RNA-Seq data. *Genome Biol* 14, (2013).

29. Li H*, et al.* The Sequence Alignment/Map format and SAMtools. *Bioinformatics* 25, 2078-2079 (2009).
30. Yang LX, et al. Diverse Mechanisms of Somatic Structural Variations in Human Cancer Genomes. *Cell* 153, 919-929 (2013).
31. Mayrhofer M, DiLorenzo S, Isaksson A. Patchwork: allele-specific copy number analysis of whole-genome sequenced tumor tissue. *Genome Biol* 14, (2013).
32. Untergasser A*, et al.* Primer3-new capabilities and interfaces. *Nucleic Acids Res* 40, (2012).
33. Neuveut C, Wei Y, Buendia MA. Mechanisms of HBV-related hepatocarcinogenesis. *J Hepatol* 52, 594-604 (2010).
34. Adey A*, et al.* The haplotype-resolved genome and epigenome of the aneuploidHeLa cancer cell line. *Nature* 500, 207-+ (2013).
35. Akagi K*, et al.* Genome-wide analysis of HPV integration in human cancers reveals recurrent, focal genomic instability. *Genome Res* 24, 185-199 (2014).
36. Sung WK*, et al.* Genome-wide survey of recurrent HBV integration in hepatocellular carcinoma. *Nat Genet* 44, 765-U188 (2012).
37. Storlazzi CT, *et al.* Gene amplification as double minutes or homogeneously staining regions in solid tumors: Origin and structure. *Genome Res* 20, 1198-1206 (2010).
38. Bouallaga I, Massicard S, Yaniv M, Thierry F. An enhanceosome containing the Jun B/Fra-2 heterodimer and the HMG-I(Y) architectural protein controls HPV18 transcription. *Embo Rep* 1, 422-427 (2000).
39. Perez-Lorenzo R*, et al.* A Tumor Suppressor Function for the Lipid Phosphatase INPP4B in Melanocytic Neoplasms. *J Invest Dermatol* 134, 1359-1368 (2014).
40. Hodgson MC*, et al.* INPP4B suppresses prostate cancer cell invasion. *Cell Commun Signal* 12, (2014).
41. Alexandrov LB, Nik-Zainal S, Wedge DC, Campbell PJ, Stratton MR. Deciphering Signatures of Mutational Processes Operative in Human Cancer. *Cell Rep* 3, 246-259 (2013).
42. Haradhvala NJ*, et al.* Mutational Strand Asymmetries in Cancer Genomes Reveal Mechanisms of DNA Damage and Repair. *Cell* 164, 538-549 (2016).

43. Ford DJ, Dingwall AK. The cancer COMPASS: navigating the functions of MLL complexes in cancer. *Cancer Genet-Ny* 208, 178-191 (2015).

44. Ojesina AI*, et al.* Landscape of genomic alterations in cervical carcinomas. *Nature* 506, 371-375 (2014).

45. Frumovitz M*, et al.*Sequencing of mutational hotspots in cancer-related genes in small cell neuroendocrine cervical cancer. *GynecolOncol* 141, 588-591 (2016).

46. George J*, et al.* Comprehensive genomic profiles of small cell lung cancer. *Nature* 524, 47-53 (2015).

47. Peifer M*, et al.* Integrative genome analyses identify key somatic driver mutations of small-cell lung cancer. *Nat Genet* 44, 1104-+ (2012).

48. Rudin CM*, et al.* Comprehensive genomic analysis identifies SOX2 as a frequently amplified gene in small-cell lung cancer. *Nat Genet* 44, 1111-+ (2012).

49. Gao JJ*, et al.* Integrative Analysis of Complex Cancer Genomics and Clinical Profiles Using the cBioPortal. *Sci Signal* 6, (2013).

50. Mermel CH, Schumacher SE, Hill B, Meyerson ML, Beroukhim R, Getz G. GISTIC2.0 facilitates sensitive and confident localization of the targets of focal somatic copy-number alteration in human cancers. *Genome Biol* 12, (2011).

51. Chen ZL*, et al.* The nuclear protein expression levels of SNAI1 and ZEB1 are involved in the progression and lymph node metastasis of cervical cancer via the epithelial-mesenchymal transition pathway. *Hum Pathol* 44, 2097-2105 (2013).

52. Li BH*, et al.* Reduced miR-100 expression in cervical cancer and precursors and its carcinogenic effect through targeting PLK1 protein. *Eur J Cancer* 47, 2166-2174 (2011).

Supplementary Figures

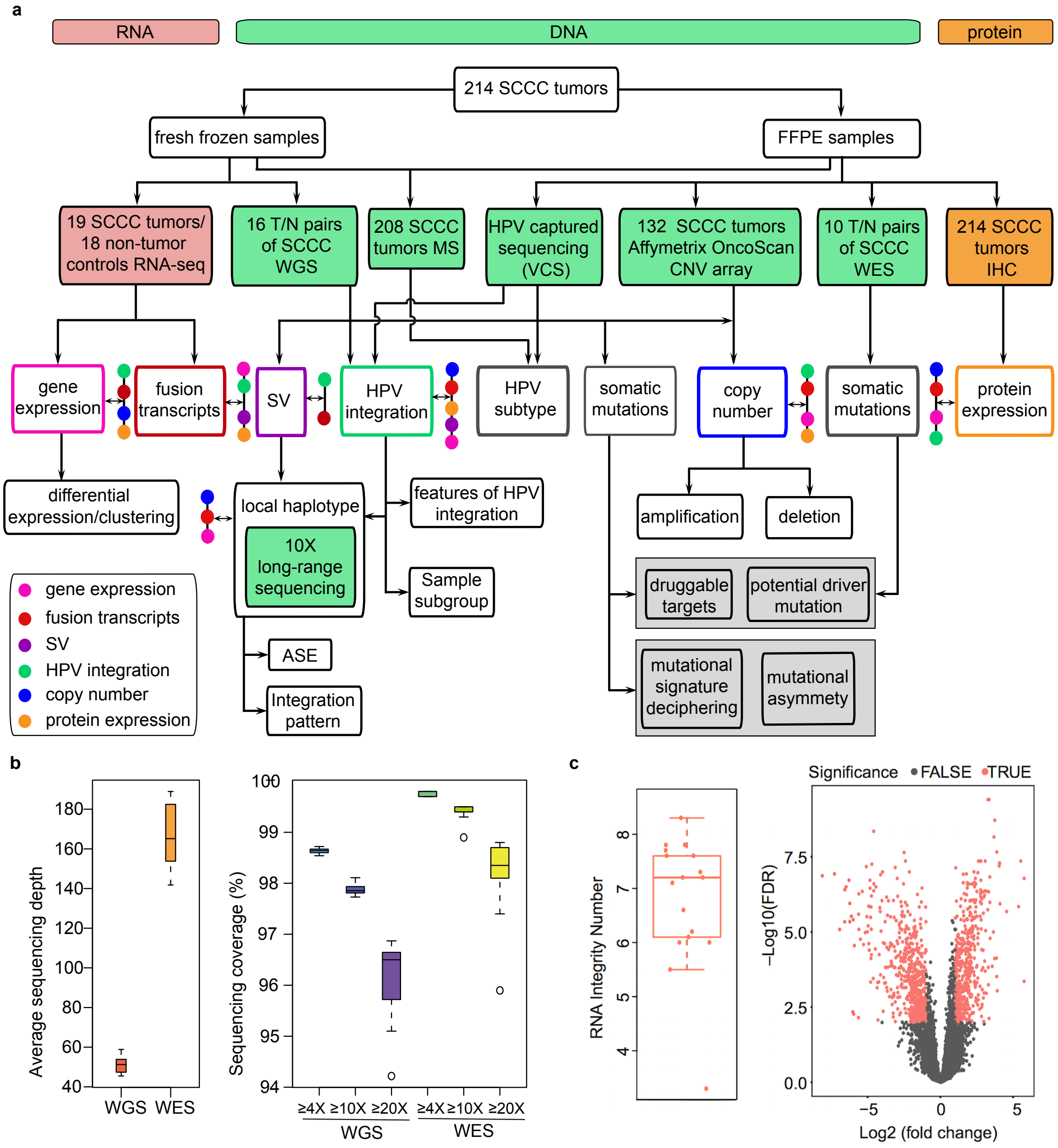

Supplementary Figure 1 | Genomic analysis in SCCC.

a, Schematic summary detailing numbers of samples and genomic analyze methods. Different colors are used for three omics levels (i.e., RNA, DNA, and protein). Colored nodes beside frame of research aspects denote the combination analysis. **b**, Average sequencing depth and coverage for whole genome sequencing (n=16) and whole exome sequencing (n=10) presented as box-plots showing median and the inter quartile range. **c**, The integrity of 19 tumor samples for RNA sequencing and scatter plot of differentially expressed genes. Box plots show centre line as median, box limits as upper and lower quartiles, whiskers as minimum to maximum values, the data point that is located outside the whiskers is defined as outlier.

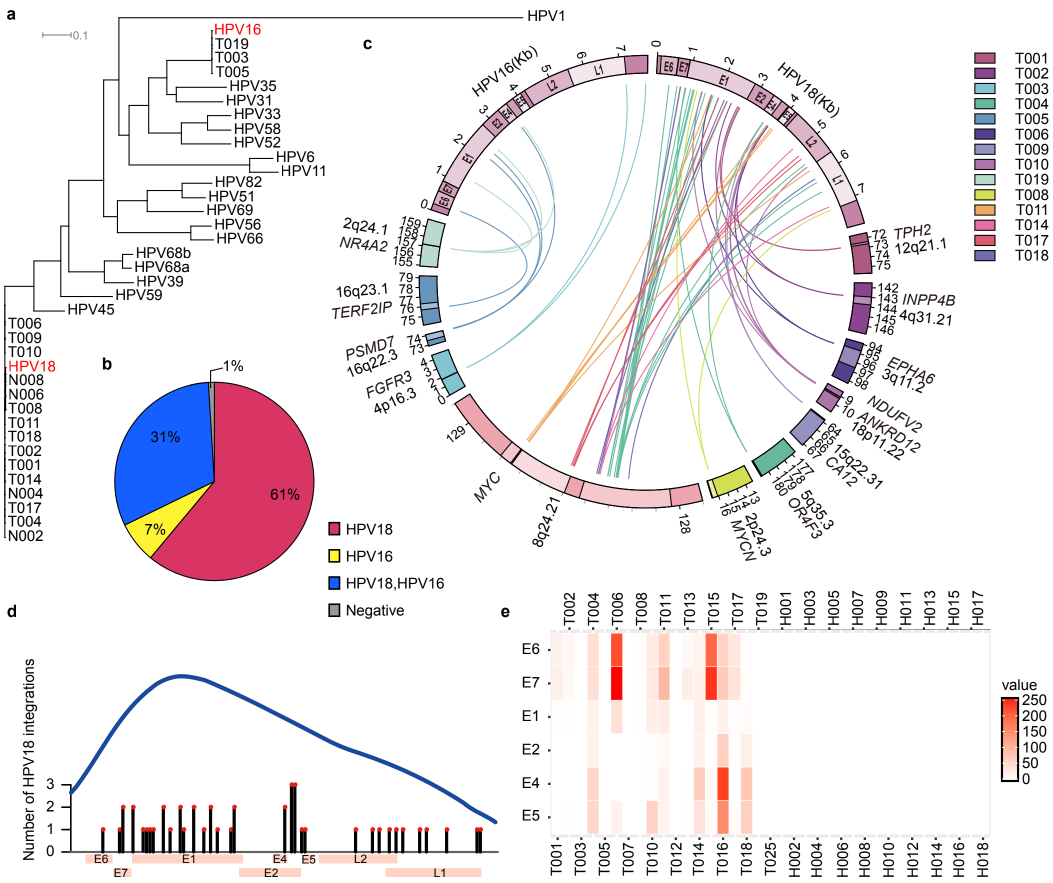

Supplementary Figure 2 | Panorama of HPV analysis in SCCC WGS samples and HPV viral gene expression.

a, Virus variant tree detected by whole genome sequencing. **b**, HPV genotyping determined by PCR-MS based method in 208 SCCC tumor samples. **c**, Circos-plot representation of HPV integration breakpoints detected by whole genome sequencing in 14 SCCC tumors. The two bars on the top denote HPV genome. The other bars denote HPV integration regions into host chromosomes. Each colored line represents a sample as shown in the legend. Human genome numbering is given in millions of bases. Genes nearest to the integration sites are list. **d**, Viral integration plot exhibiting break points along HPV18 reference genome in 11 SCCC tumors. The blue line indicates the distribution density curve of integration breakpoints in the HPV18 positive genome. **e**, HPV viral gene expression obtained from RNA sequencing in 19 tumors and 18 non-tumor cervix samples.

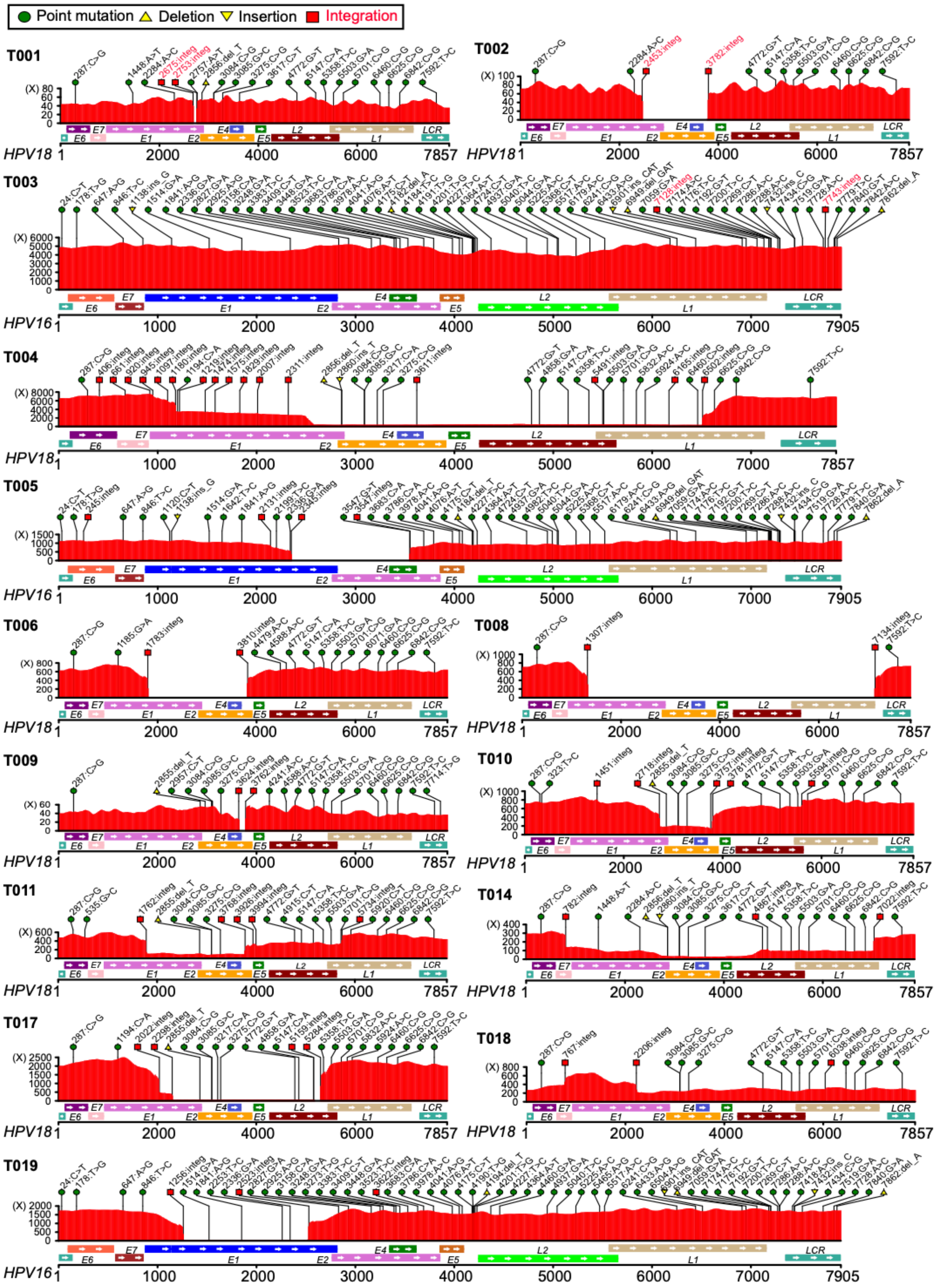

Supplementary Figure 3 | Constructed individual HPV genomes in SCCC WGS samples.

Depth distribution along HPV genomes were displayed with mutations used to construct individual HPV genomes. The integration breakpoints were also plotted.

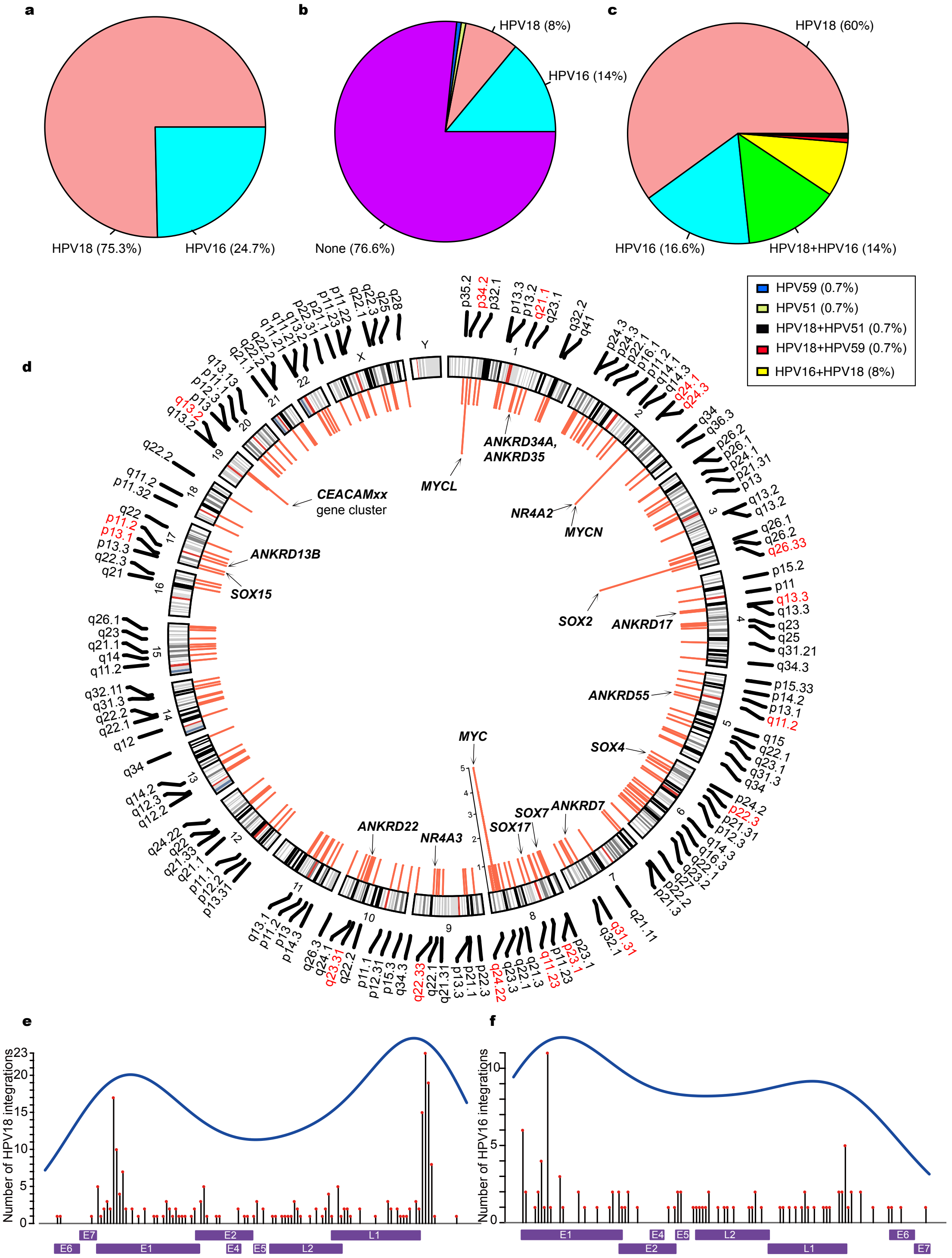

Supplementary Figure 4 | Panorama of HPV analysis in SCCC VCS samples.

a, Distribution of first HPV subtypes. **b**, Distribution of second HPV subtypes. Note that about 76.6% samples (the '*None*' set) lack the second HPV subtypes. **c**, Distribution of first and second HPV subtypes. First HPV subtype is written ahead of second HPV subtype, e.g., 'HPV18+HPV16'. **d**, Circos-plot representation of major HPV integration breakpoints detected

from VCS data in 150 SCCC FFPE samples. Window size is 50kb. Genes from enriched gene families are annotated at their loci with the relevant cytoband written in red. **e**, Viral break points of major HPV integration along HPV18 reference genome (NC_001357.1) in 150 SCCC FFPE samples. The blue line indicates the distribution density curve of breakpoints. **f**, Viral break points of major HPV integration along HPV16 reference genome (NC_001526.4) in 150 SCCC FFPE samples.

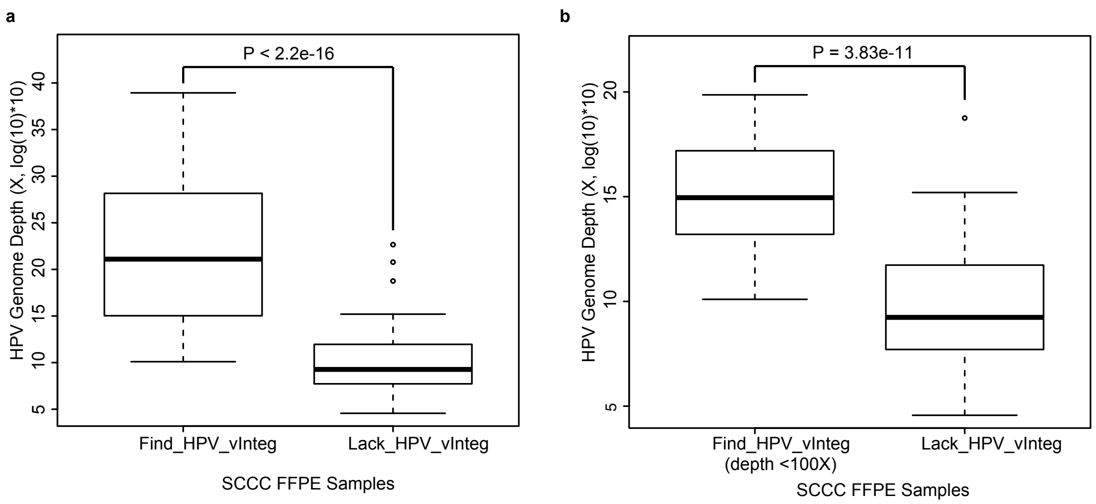

Supplementary Figure 5 | HPV genome depth comparison from VCS analysis of 150 SCCC FFPE samples.

a, Average depth comparison of HPV genomes of samples that have (n=81) or lack (n=69) HPV integrations (Mann-Whitney U Test, two-sided, P< 2.2e-16). Note that values of the average depth are transformed. **b**, HPV genomes of which average depth larger than 100X are filtered out from '*Find_HPV_vInteg*' sample set (n=39, Mann-Whitney U Test, two-sided, P=3.83e-11)**.** Box plots show centre line as median, box limits as upper and lower quartiles, whiskers as minimum to maximum values, the data point that is located outside the whiskers is defined as outlier.

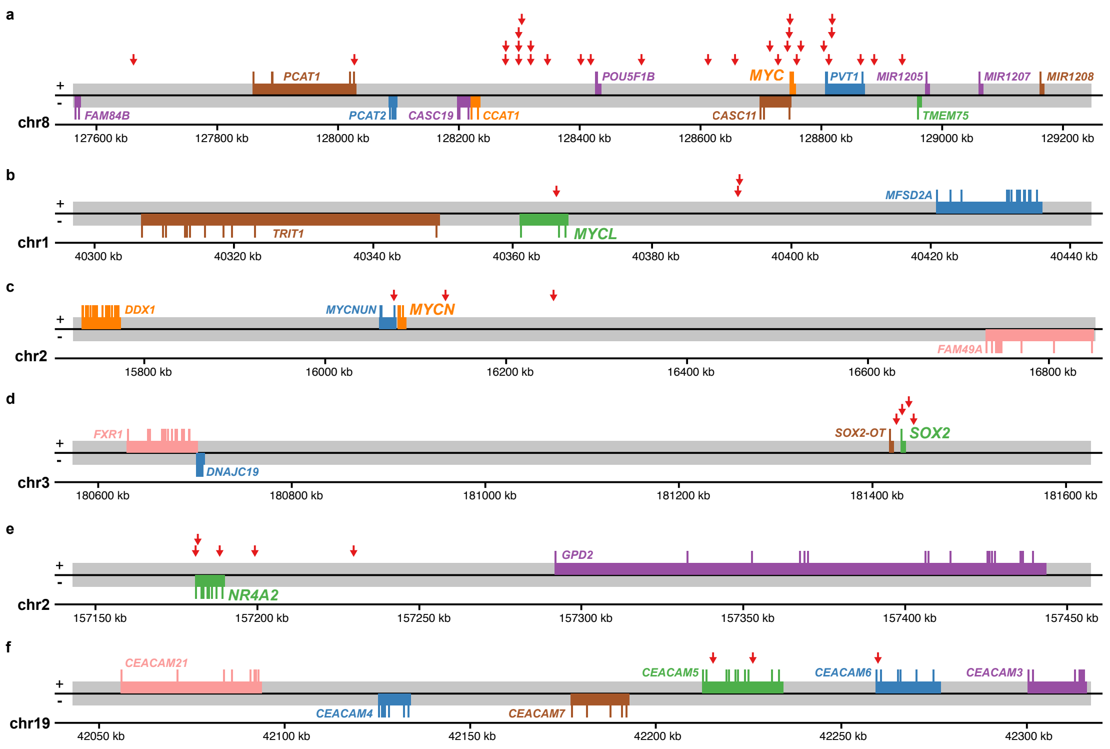

Supplementary Figure 6 | HPV integration hotspots.

The HPV integrations reside at recurrently affected genes from enriched gene families. MYC family: *MYC* (a), *MYCL* (b), *MYCN* (c); SOX family: *SOX2* (d); NR4A family: *NR4A2* (e); CEA family: *CEACAM* cluster (f). Red arrows, locations of HPV-integrated breakpoints in one given sample. If one sample had multiple integrations in genomic region of one panel, only select the one that had maximum amount of junction reads. Chr, chromosome; colored boxes, gene regions; bulges, exons.

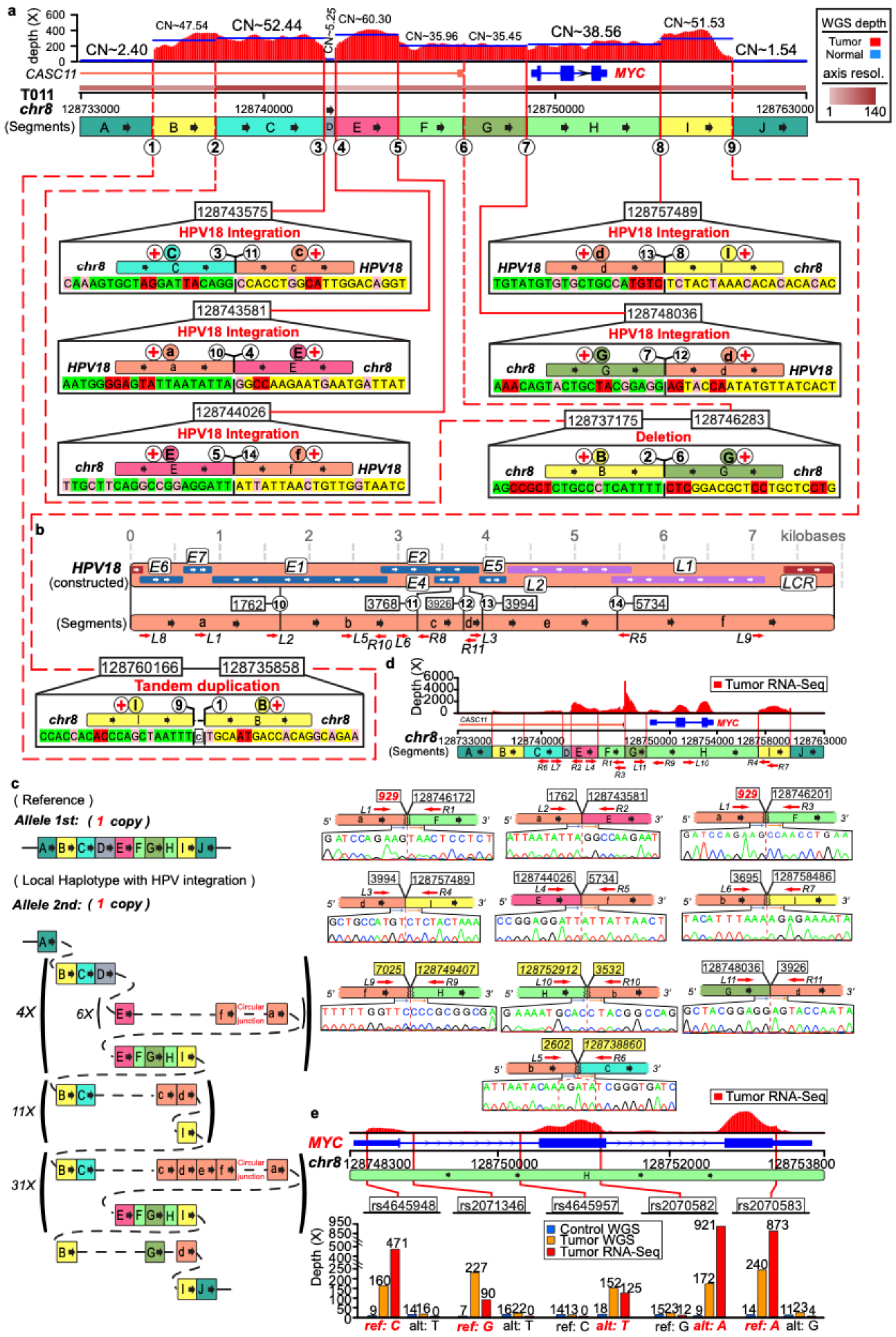

Supplementary Figure 7 | Presentative local haplotype of HPV integration sites of sample T011.

Introduction of each panel is similar to that of Figure 2. In panel a, Human segments noted by red asterisks have copy number shifts from the observed in resolved local haplotypes (Supplementary Data 13). In panel b, Constructed HPV18 genome is segmented by breakpoints with circled numbers corresponding to boxes above. In panel c, all HPV integrated local haplotypes shown are Simplest type (Supplementary Note 10). In panel d, Frames filled in yellow indicate some HPV-human fusion cases from the third category (Supplementary Note 11) failed to validate the possibly missed integrations in the DNA level, but still succeeded in the cDNA level. In panel **e**, ASE positions in red indicate considerable shift from expected in validation experiment, and the reads amount in red specify the corresponding tissue.

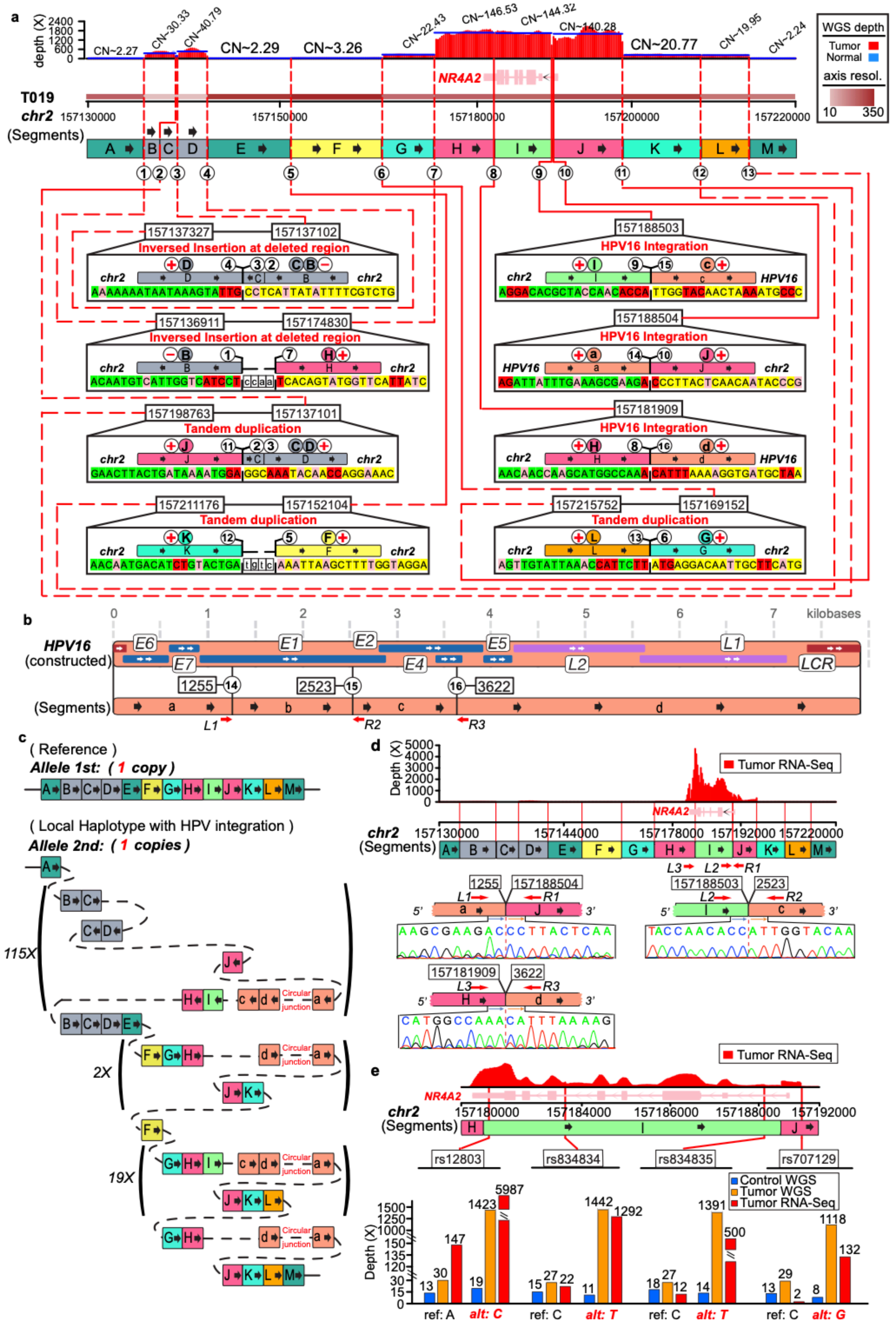

Supplementary Figure 8 | Presentative local haplotype of HPV integration sites of sample T019.

Introduction of each panel is similar to that of Figure 2. In panel a, Human segments noted by red asterisks have copy number shifts from the observed in resolved local haplotypes. In panel b,

Constructed HPV16 genome is segmented by breakpoints with circled numbers corresponding to boxes above. In panel c, all HPV integrated local haplotypes shown are Simplest type. In panel d, Frames filled in yellow indicate some HPV-human fusion cases from the third category failed to validate the possibly missed integrations in the DNA level, but still succeeded in the cDNA level. In panel **e**, ASE positions in red indicate considerable shift from expected in validation experiment, and the reads amount in red specify the corresponding tissue.

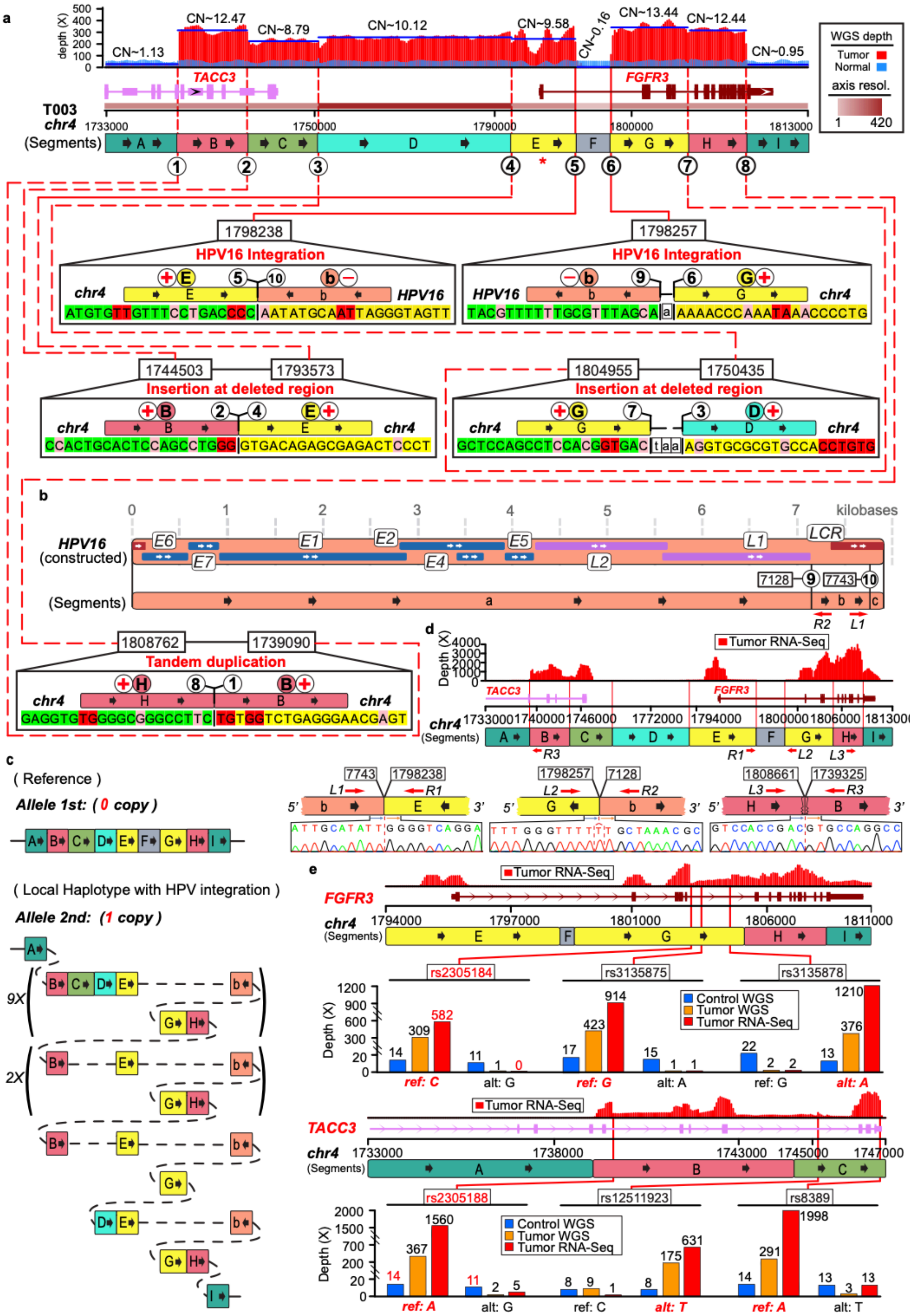

Supplementary Figure 9 | Presentative local haplotype of HPV integration sites of sample T003.

Introduction of each panel is similar to that of Figure 2. In panel a, Human segments noted by red asterisks have copy number shifts from the observed in resolved local haplotypes. In panel b, Constructed HPV16 genome is segmented by breakpoints with circled numbers corresponding to boxes above. In panel c, all HPV integrated local haplotypes shown are Simplest type. In panel d, Frames filled in yellow indicate some HPV-human fusion cases from the third category failed to validate the possibly missed integrations in the DNA level, but still succeeded in the cDNA level. In panel **e**, ASE positions in red indicate considerable shift from expected in validation experiment, and the reads amount in red specify the corresponding tissue.

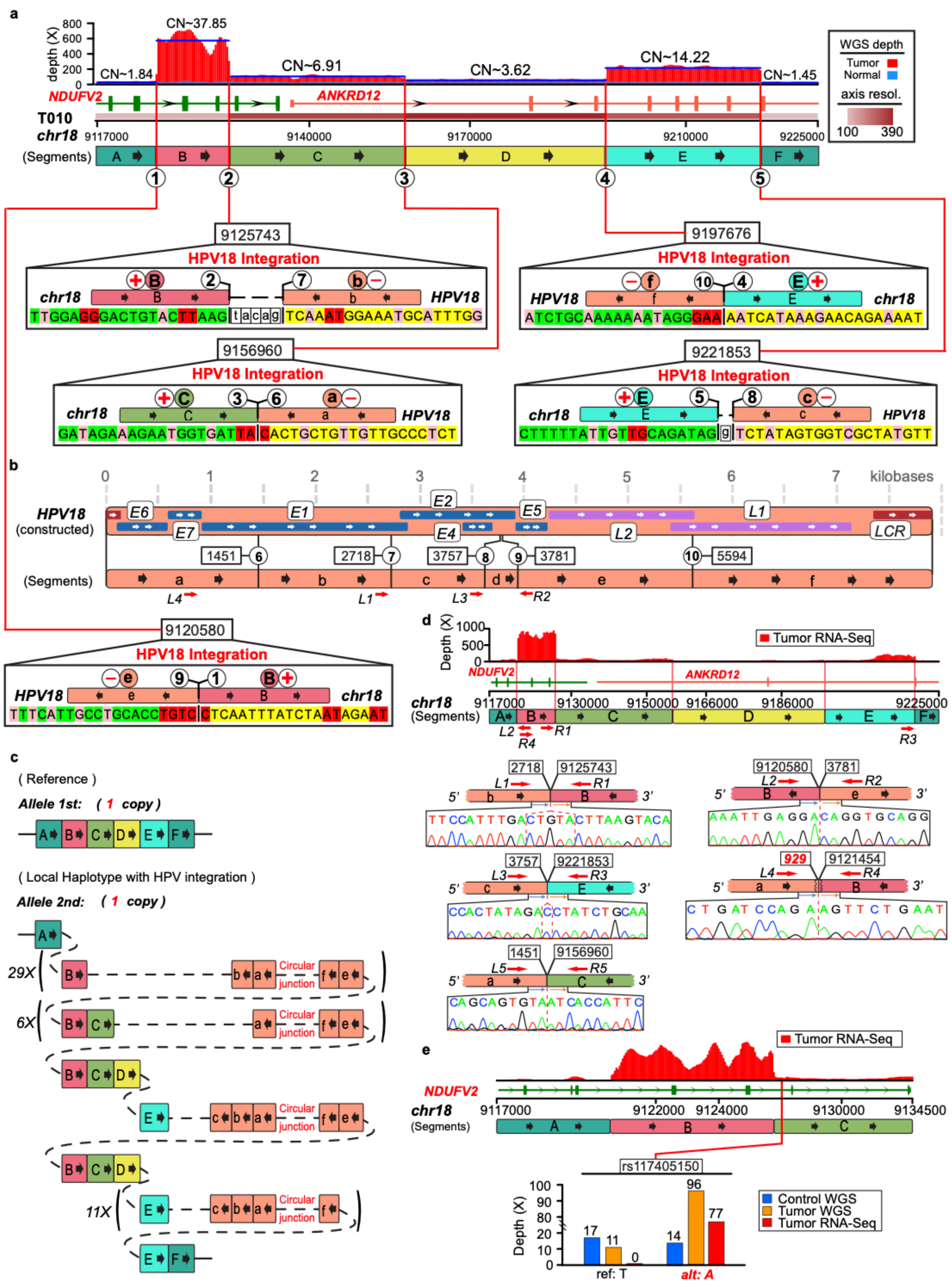

Supplementary Figure 10 | Presentative local haplotype of HPV integration sites of sample T010.

Introduction of each panel is similar to that of Figure 2. In panel a, Human segments noted by red asterisks have copy number shifts from the observed in resolved local haplotypes. In panel b, Constructed HPV18 genome is segmented by breakpoints with circled numbers corresponding to boxes above. In panel c, all HPV integrated local haplotypes shown are Simplest type. In panel d, Frames filled in yellow indicate some HPV-human fusion cases from the third category failed to validate the possibly missed integrations in the DNA level, but still succeeded in the cDNA level.

In panel **e**, ASE positions in red indicate considerable shift from expected in validation experiment, and the reads amount in red specify the corresponding tissue.

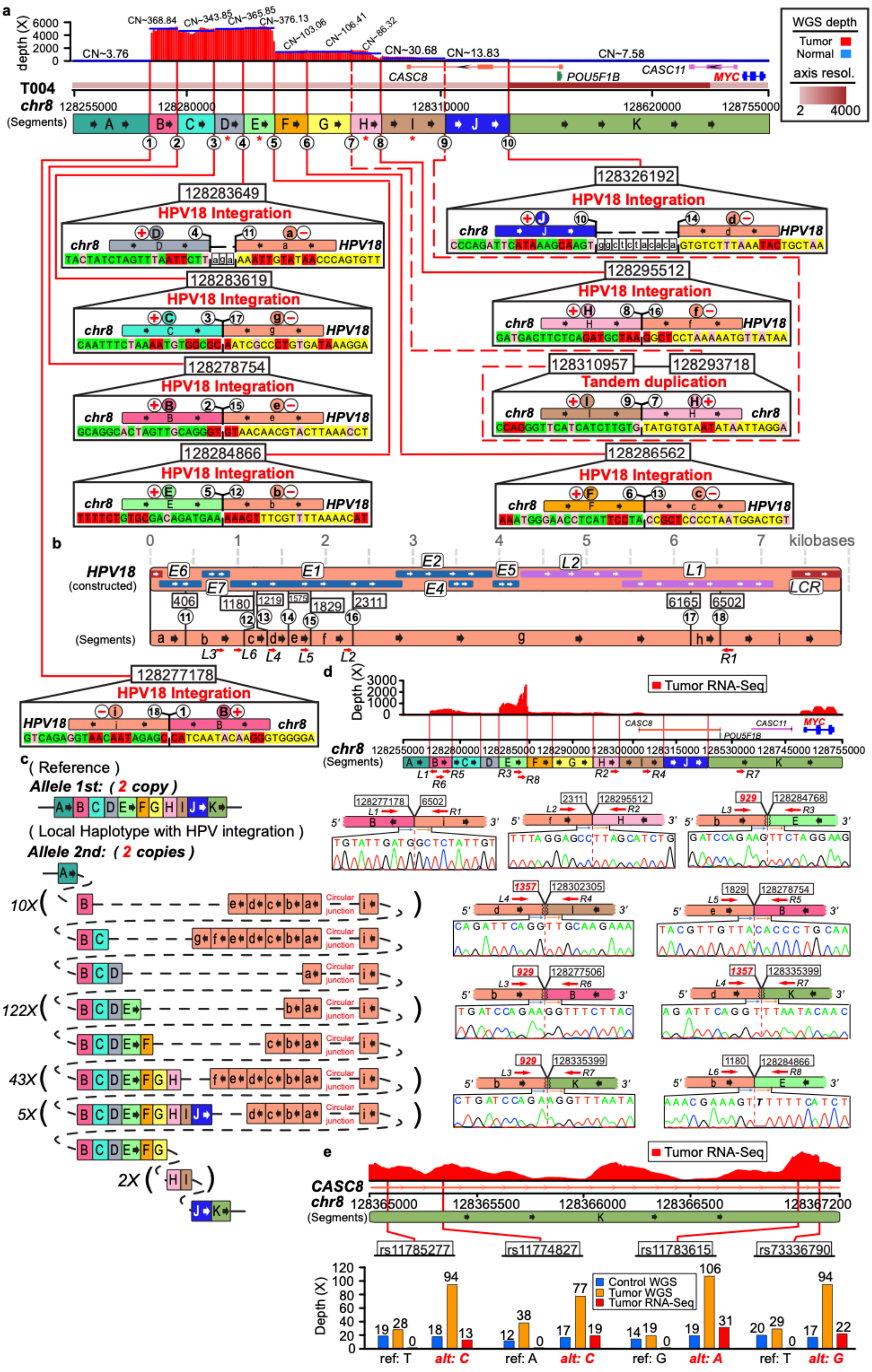

Supplementary Figure 11 | Presentative local haplotype of HPV integration sites of sample T004.

Introduction of each panel is similar to that of Figure 2. In panel a, Human segments noted by red asterisks have copy number shifts from the observed in resolved local haplotypes. In panel b, Constructed HPV18 genome is segmented by breakpoints with circled numbers corresponding to boxes above. In panel c, all HPV integrated local haplotypes shown are Simplest type. In panel d, Frames filled in yellow indicate some HPV-human fusion cases from the third category failed to validate the possibly missed integrations in the DNA level, but still succeeded in the cDNA level. In panel **e**, ASE positions in red indicate considerable shift from expected in validation experiment, and the reads amount in red specify the corresponding tissue.

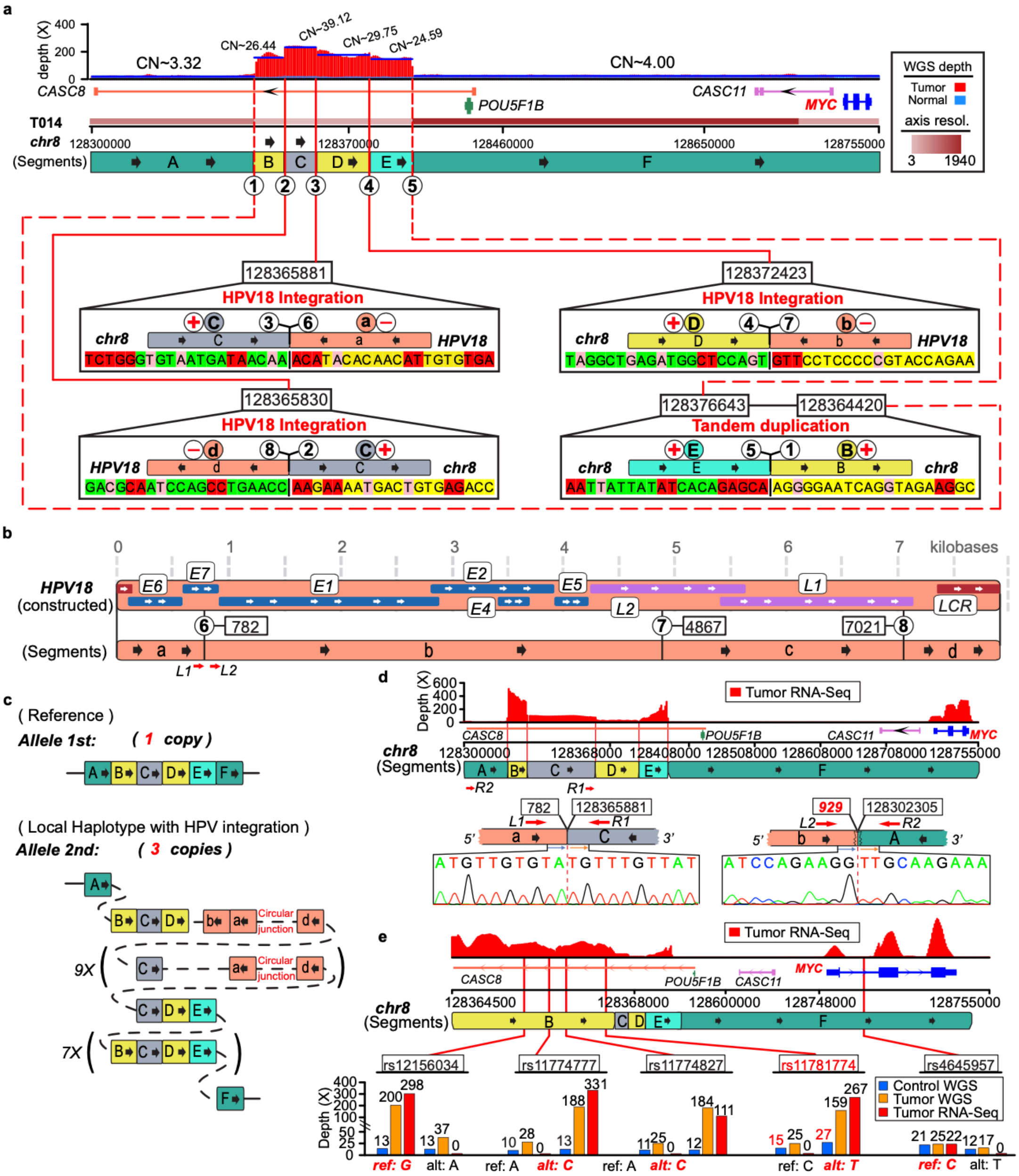

Supplementary Figure 12 | Presentative local haplotype of HPV integration sites of sample T014.

Introduction of each panel is similar to that of Figure 2. In panel a, Human segments noted by red asterisks have copy number shifts from the observed in resolved local haplotypes. In panel b, Constructed HPV18 genome is segmented by breakpoints with circled numbers corresponding to

boxes above. In panel c, all HPV integrated local haplotypes shown are Simplest type. In panel d, Frames filled in yellow indicate some HPV-human fusion cases from the third category failed to validate the possibly missed integrations in the DNA level, but still succeeded in the cDNA level. In panel **e**, ASE positions in red indicate considerable shift from expected in validation experiment, and the reads amount in red specify the corresponding tissue.

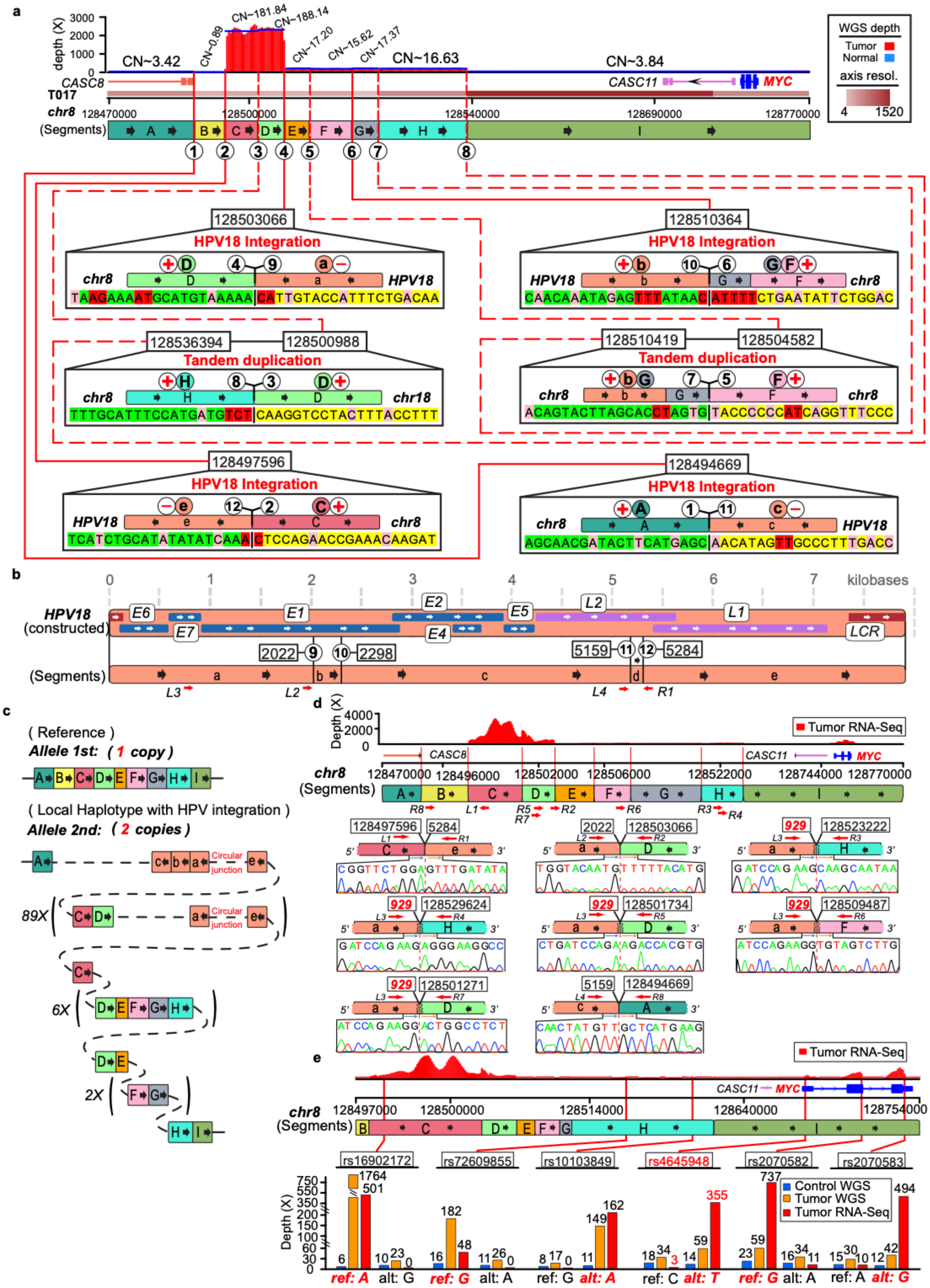

Supplementary Figure 13 | Presentative local haplotype of HPV integration sites of sample T017.

Introduction of each panel is similar to that of Figure 2. In panel a, Human segments noted by red asterisks have copy number shifts from the observed in resolved local haplotypes. In panel b, Constructed HPV18 genome is segmented by breakpoints with circled numbers corresponding to boxes above. In panel c, all HPV integrated local haplotypes shown are Simplest type. In panel d, Frames filled in yellow indicate some HPV-human fusion cases from the third category failed to validate the possibly missed integrations in the DNA level, but still succeeded in the cDNA level. In panel **e**, ASE positions in red indicate considerable shift from expected in validation experiment, and the reads amount in red specify the corresponding tissue.

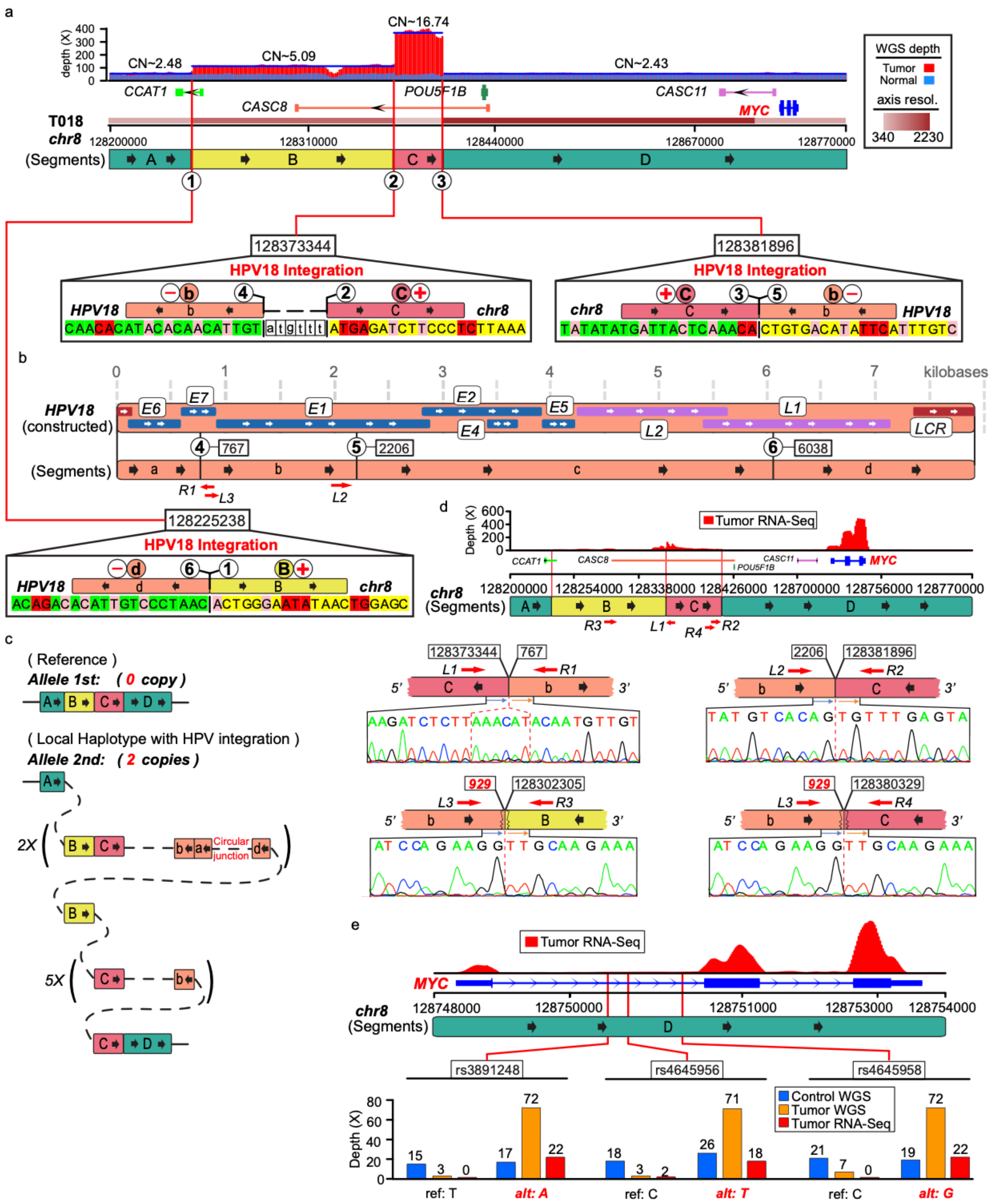

Supplementary Figure 14 | Presentative local haplotype of HPV integration sites of sample T018.

Introduction of each panel is similar to that of Figure 2. In panel a, Human segments noted by red asterisks have copy number shifts from the observed in resolved local haplotypes. In panel b, Constructed HPV18 genome is segmented by breakpoints with circled numbers corresponding to boxes above. In panel c, all HPV integrated local haplotypes shown are Simplest type. In panel d, Frames filled in yellow indicate some HPV-human fusion cases from the third category failed to validate the possibly missed integrations in the DNA level, but still succeeded in the cDNA level. In panel **e**, ASE positions in red indicate considerable shift from expected in validation experiment, and the reads amount in red specify the corresponding tissue.

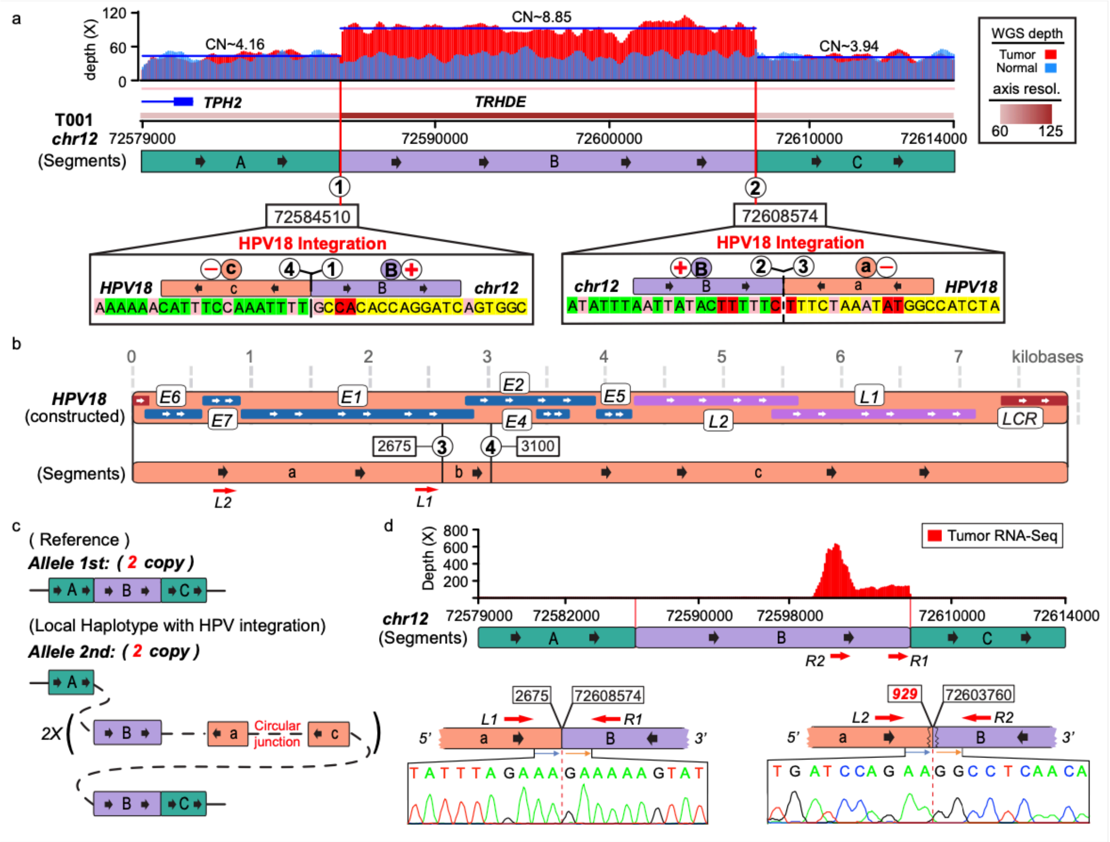

Supplementary Figure 15 | Presentative local haplotype of HPV integration sites of sample T001.

Introduction of each panel is similar to that of Figure 2. In panel a, Human segments noted by red asterisks have copy number shifts from the observed in resolved local haplotypes. In panel b, Constructed HPV18 genome is segmented by breakpoints with circled numbers corresponding to boxes above. In panel c, all HPV integrated local haplotypes shown are Simplest type. In panel d, Frames filled in yellow indicate some HPV-human fusion cases from the third category failed to validate the possibly missed integrations in the DNA level, but still succeeded in the cDNA level.

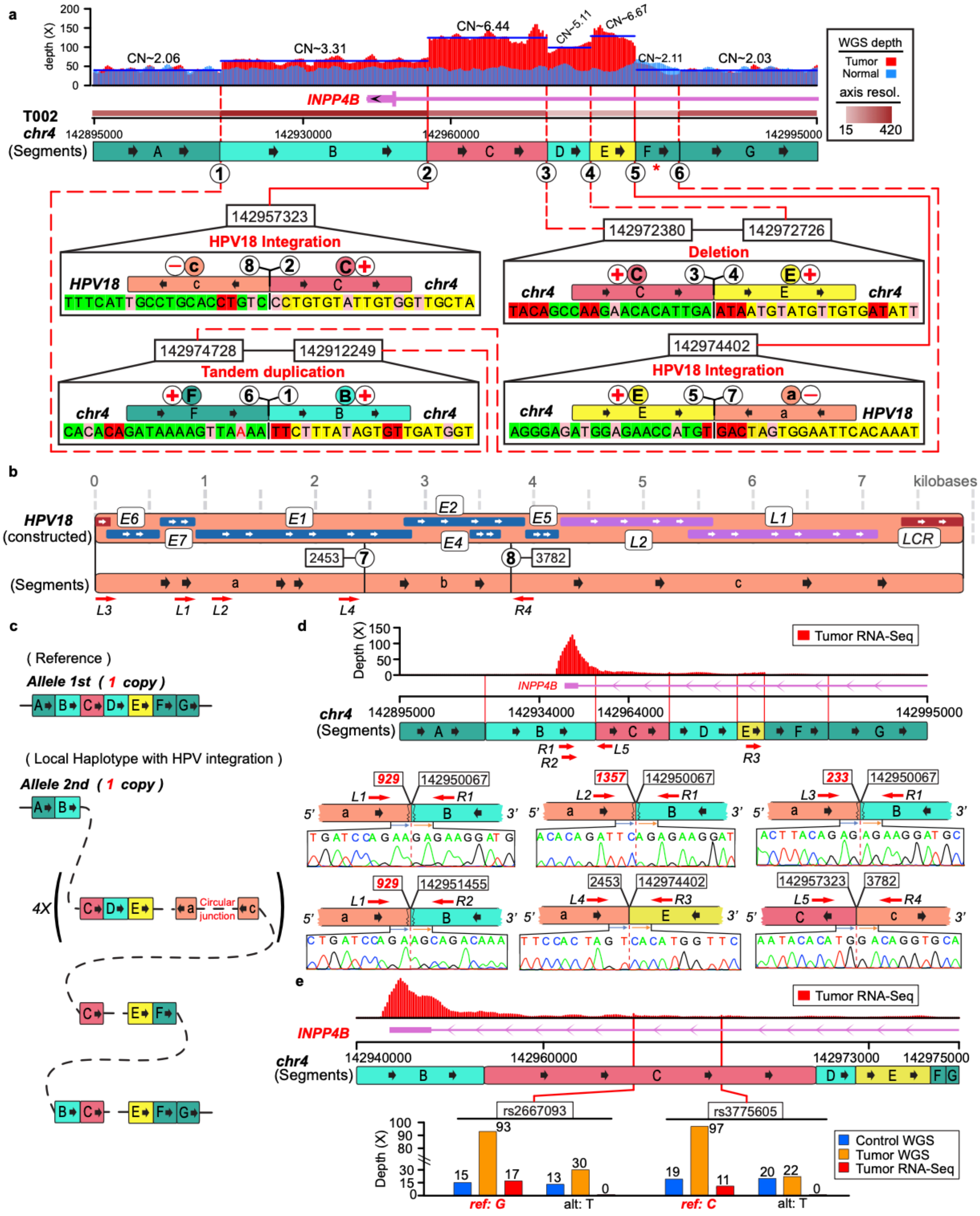

Supplementary Figure 16 | Presentative local haplotype of HPV integration sites of sample T002.

Introduction of each panel is similar to that of Figure 2. In panel a, Human segments noted by red asterisks have copy number shifts from the observed in resolved local haplotypes. In panel b, Constructed HPV18 genome is segmented by breakpoints with circled numbers corresponding to boxes above. In panel c, all HPV integrated local haplotypes shown are Simplest type. In panel d, Frames filled in yellow indicate some HPV-human fusion cases from the third category failed to validate the possibly missed integrations in the DNA level, but still succeeded in the cDNA level. In panel **e**, ASE positions in red indicate considerable shift from expected in validation experiment, and the reads amount in red specify the corresponding tissue.

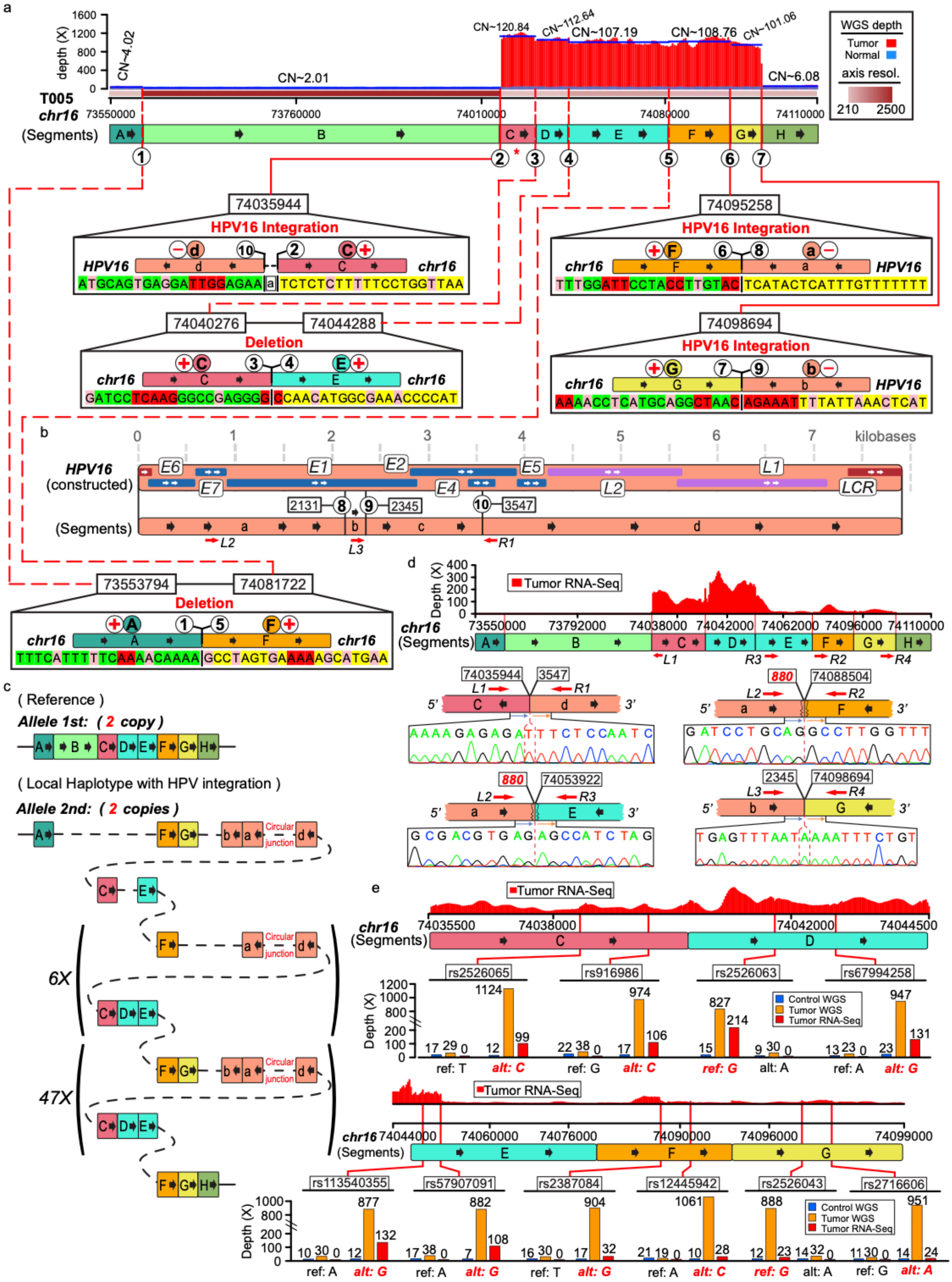

Supplementary Figure 17 | Presentative local haplotype of HPV integration sites of sample T005.

Introduction of each panel is similar to that of Figure 2. In panel a, Human segments noted by red asterisks have copy number shifts from the observed in resolved local haplotypes. In panel b, Constructed HPV16 genome is segmented by breakpoints with circled numbers corresponding to boxes above. In panel c, all HPV integrated local haplotypes shown are Simplest type. In panel d, Frames filled in yellow indicate some HPV-human fusion cases from the third category failed to validate the possibly missed integrations in the DNA level, but still succeeded in the cDNA level.

In panel **e**, ASE positions in red indicate considerable shift from expected in validation experiment, and the reads amount in red specify the corresponding tissue.

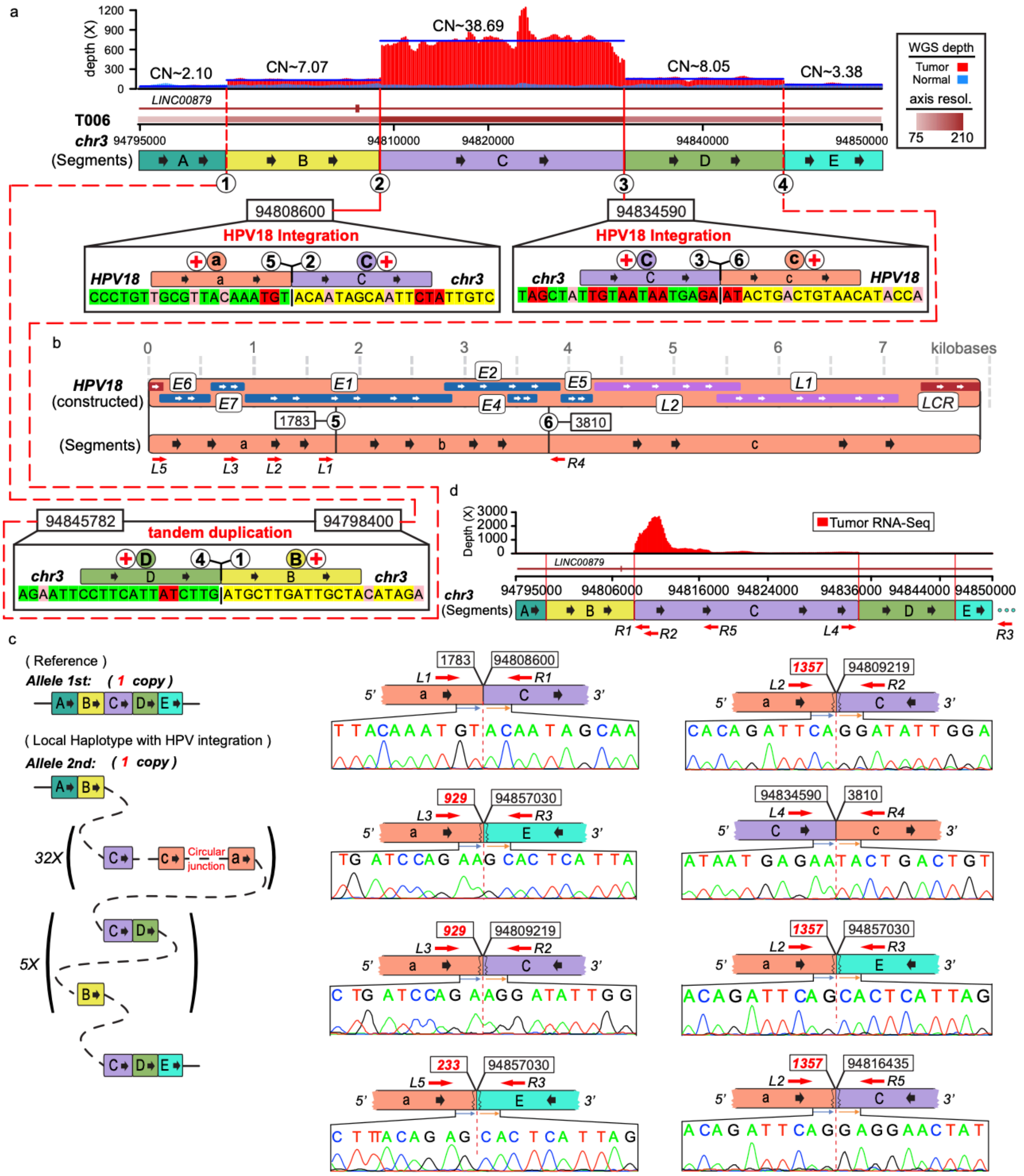

Supplementary Figure 18 | Presentative local haplotype of HPV integration sites of sample T006.

Introduction of each panel is similar to that of Figure 2. In panel a, Human segments noted by red asterisks have copy number shifts from the observed in resolved local haplotypes. In panel b, Constructed HPV18 genome is segmented by breakpoints with circled numbers corresponding to boxes above. In panel c, all HPV integrated local haplotypes shown are Simplest type. In panel d, Frames filled in yellow indicate some HPV-human fusion cases from the third category failed to validate the possibly missed integrations in the DNA level, but still succeeded in the cDNA level.

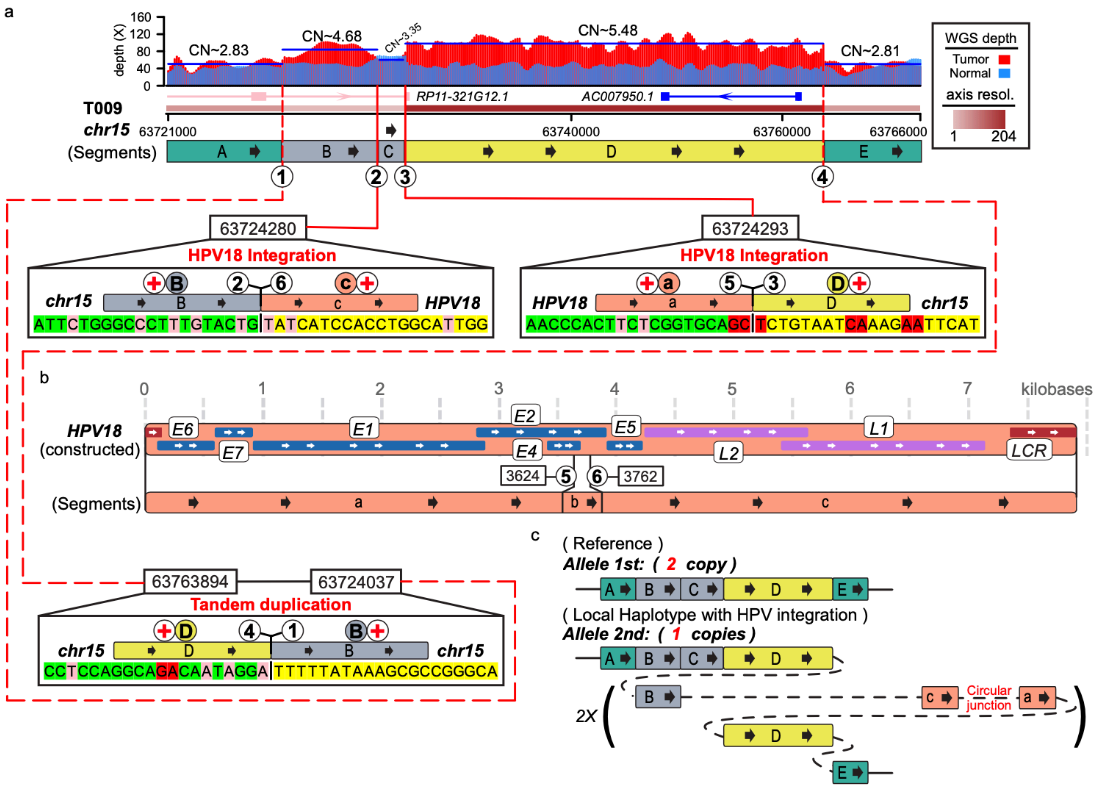

Supplementary Figure 19 | Presentative local haplotype of HPV integration sites of sample T009.

S

Introduction of each panel is similar to that of Figure 2. In panel a, Human segments noted by red asterisks have copy number shifts from the observed in resolved local haplotypes. In panel b, Constructed HPV18 genome is segmented by breakpoints with circled numbers corresponding to boxes above. In panel c, all HPV integrated local haplotypes shown are Simplest type.

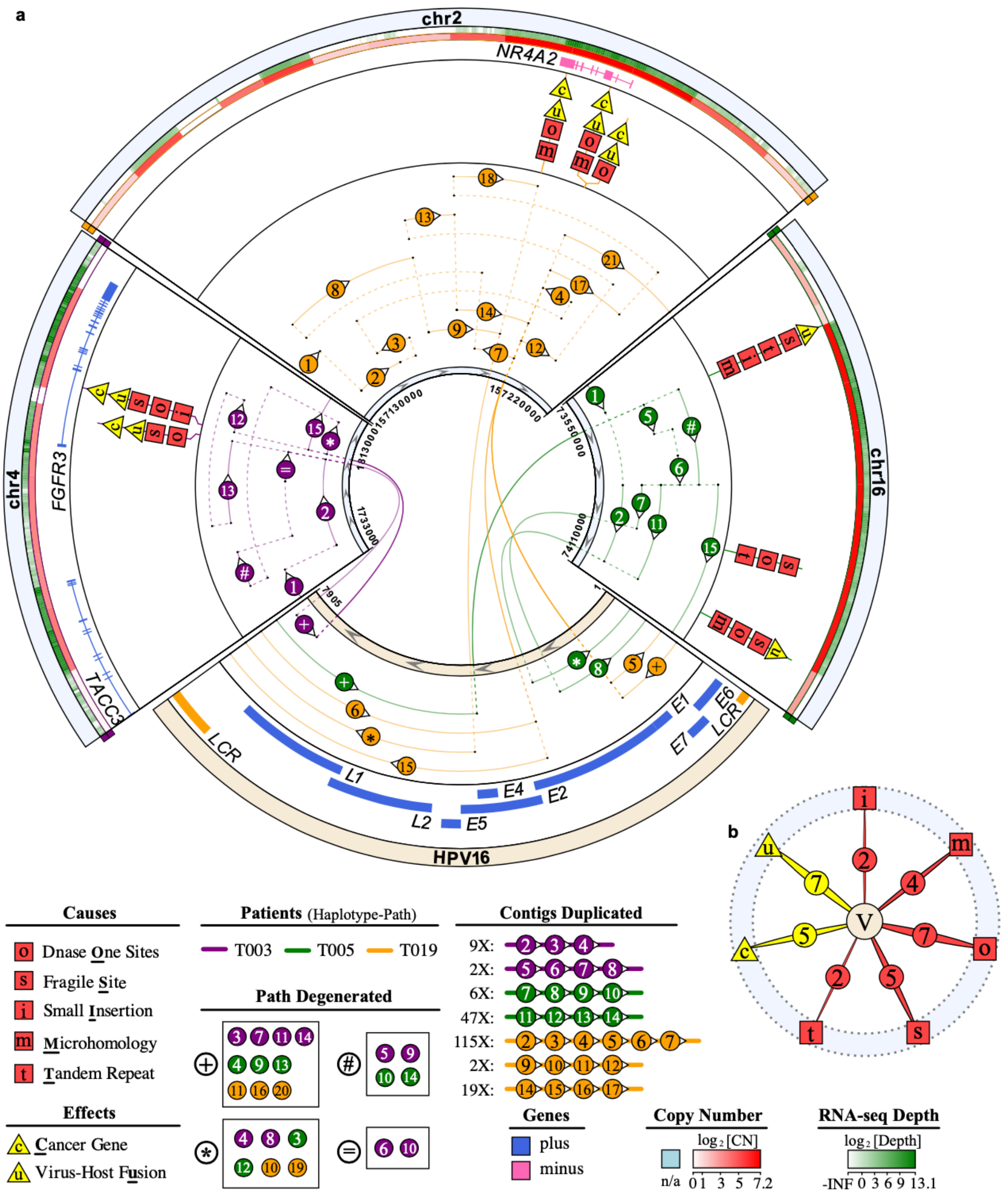

Supplementary Figure 20 | Features of HPV16 integrated local haplotypes.

a, Human genomic segments related to HPV16 integrated local haplotypes are shown as sectors with their relevant haplotype path in sample specific colours. Regions of local haplotypes are noted by circled numbers in sequence, where some are degenerated by symbols for simplification. Repeated times of contigs in local haplotypes are stated in figure legend. Features of all HPV16 integrated sites are depicted as single-letter icons. DNA copy number (CN) and RNA-seq transcription abundance were displayed in gradient colour with relevant sample colour-notes bilaterally (red for CN, green for RNA-seq depth). **b**, Statistics counts of

features of HPV16 integrated sites. V in circle centre means HPV16 genome, and outer band in light blue means human genome.

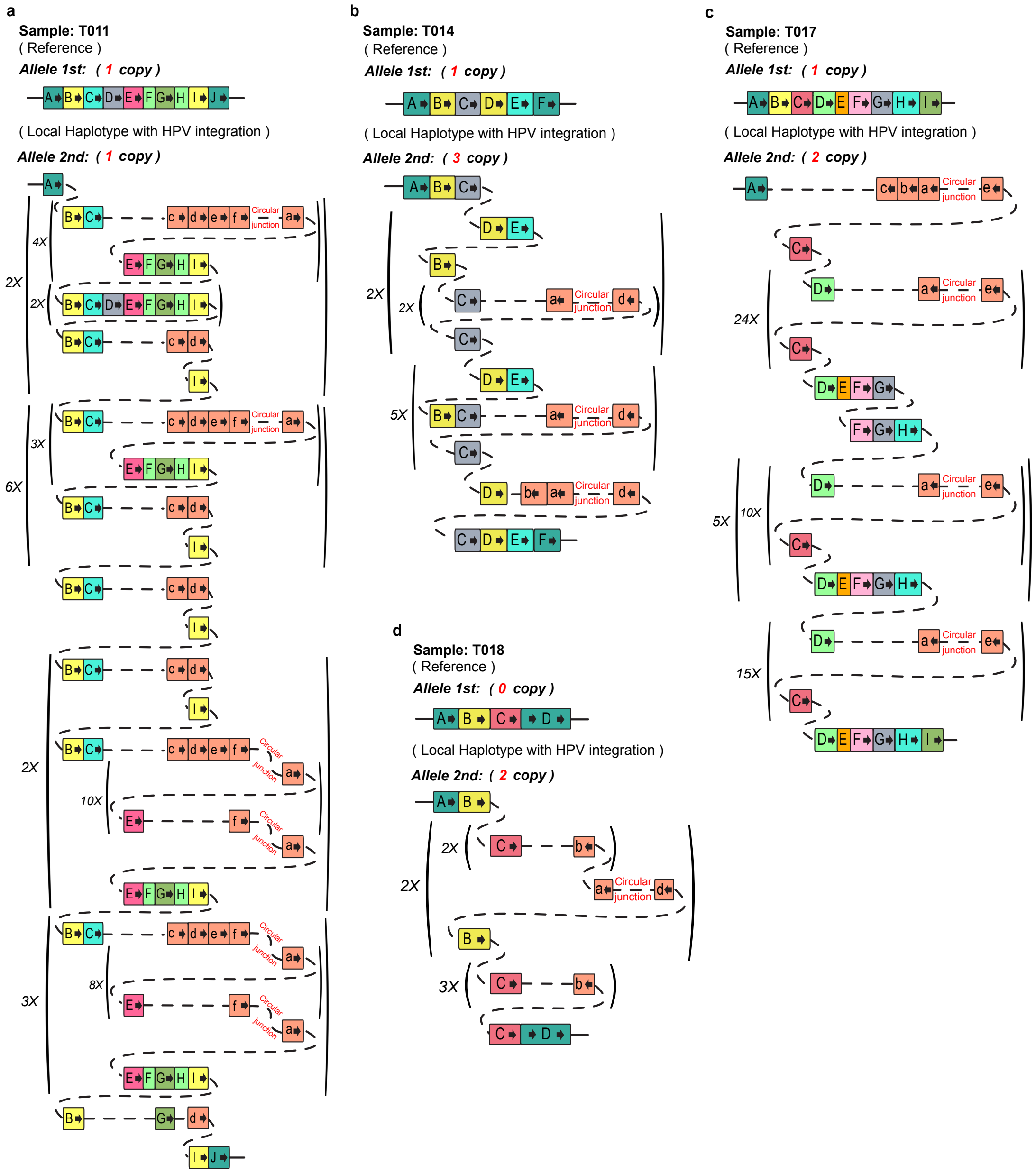

Supplementary Figure 21 | Optimized local haplotypes of HPV integration sites.

a-d, According to the number of shared barcodes of pair-wise anchors, local haplotype of HPV integrations in T011 (**a**) T014 (**b**), T017 (**c**), and T018 (**d**) were optimized from the Simplest type structure (Supplementary Figure 7c) to Random-Best structure. This improved the correlation between the 10x long-range linked-reads sequencing data and the structure of local haplotype (Supplementary Note 10 and 13).

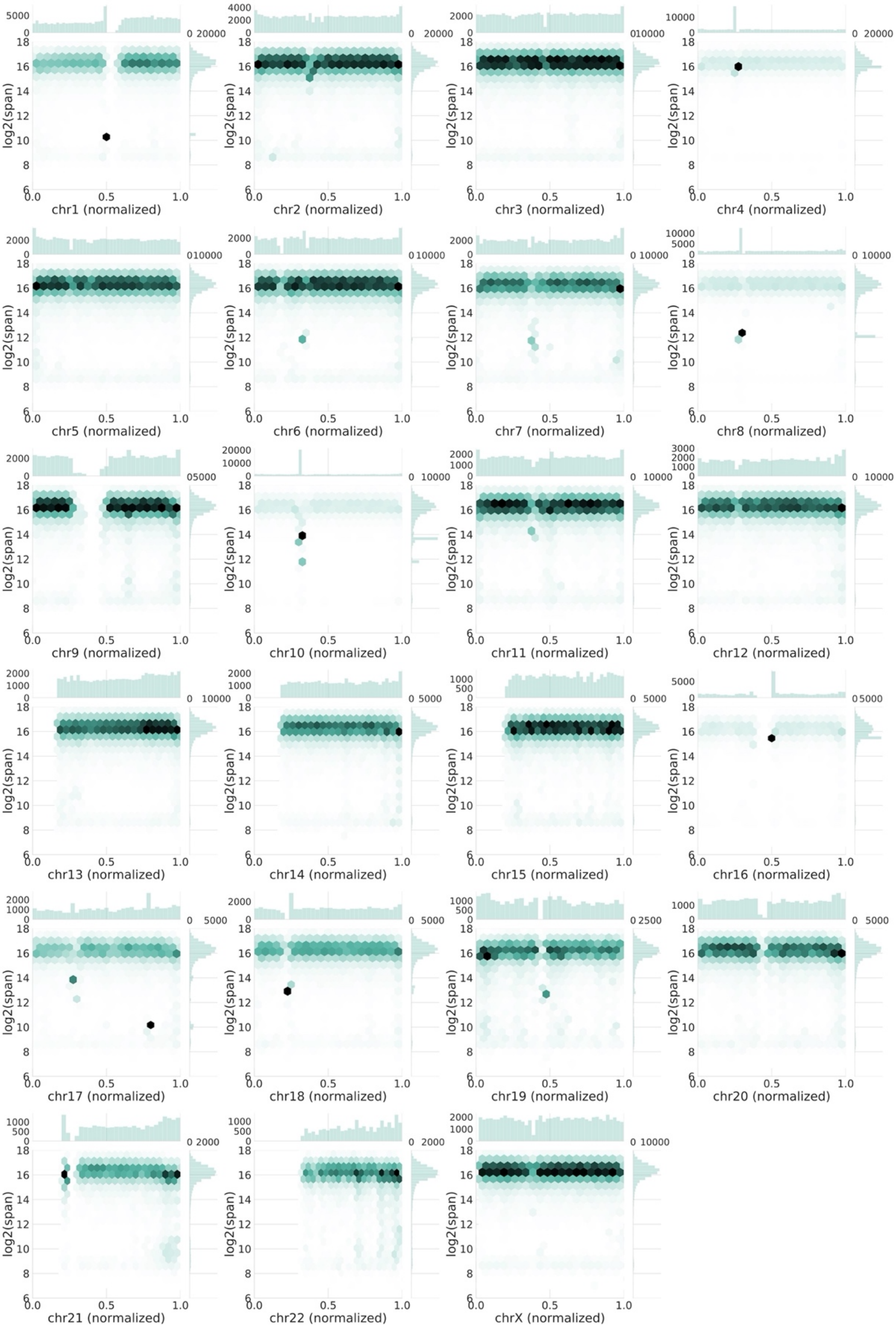

Supplementary Figure 22 | Distribution of 10x linked-reads barcode spanning size in sample T011.

Distribution of spanning size of 10x sequencing barcodes was displayed along the twenty-three

chromosomes. Y axis shows the log under 2 of spanning size of barcodes, and the X axis shows the normalized (0.0-1.0) positions of chromosome. As the SCCC samples are all from women, the Y chromosome was ignored.

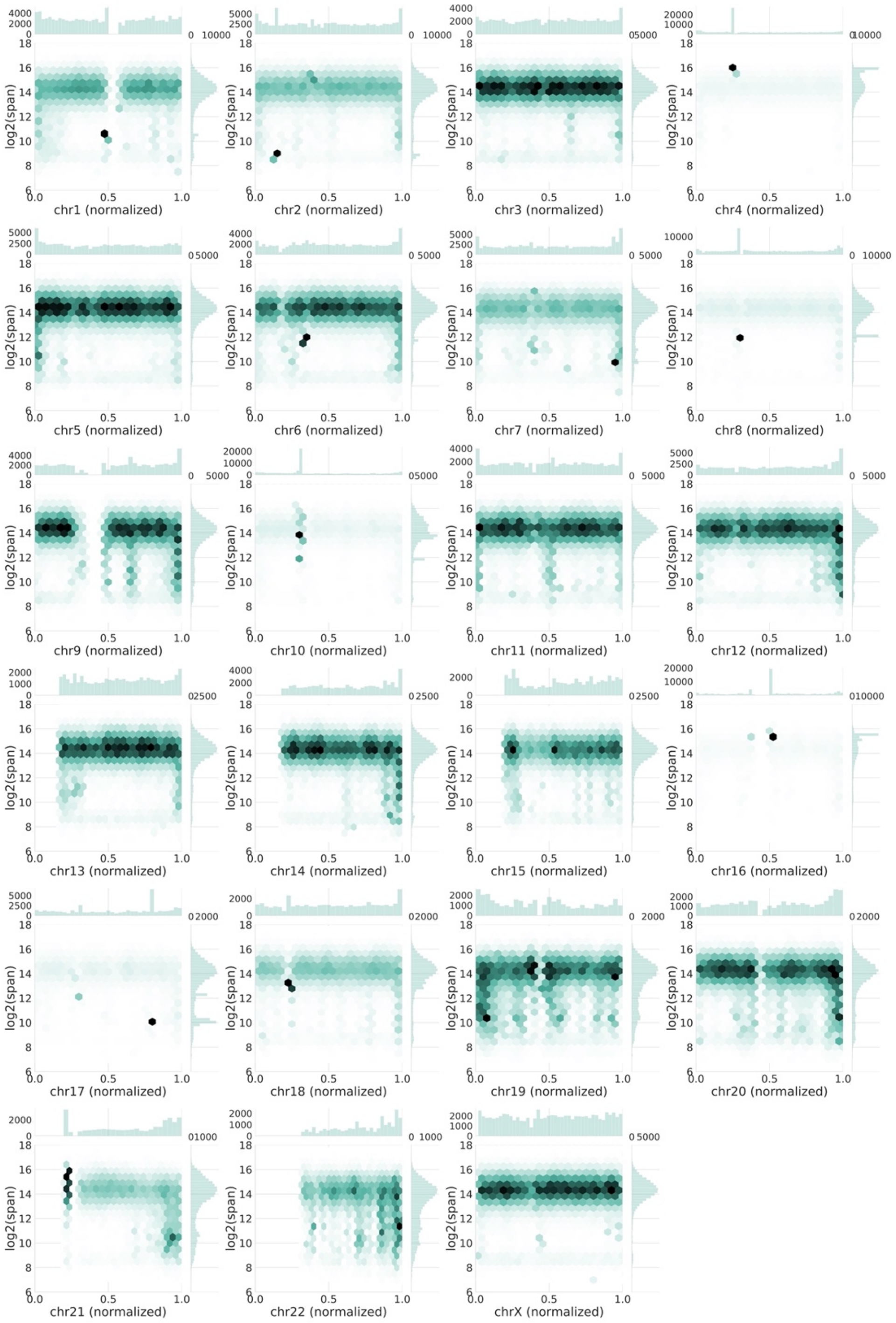

Supplementary Figure 23 | Distribution of 10x linked-reads barcode spanning size in sample T014.

Distribution of spanning size of 10x sequencing barcodes was displayed along the twenty-three

chromosomes. Y axis shows the log under 2 of spanning size of barcodes, and the X axis shows the normalized (0.0-1.0) positions of chromosome. As the SCCC samples are all from women, the Y chromosome was ignored.

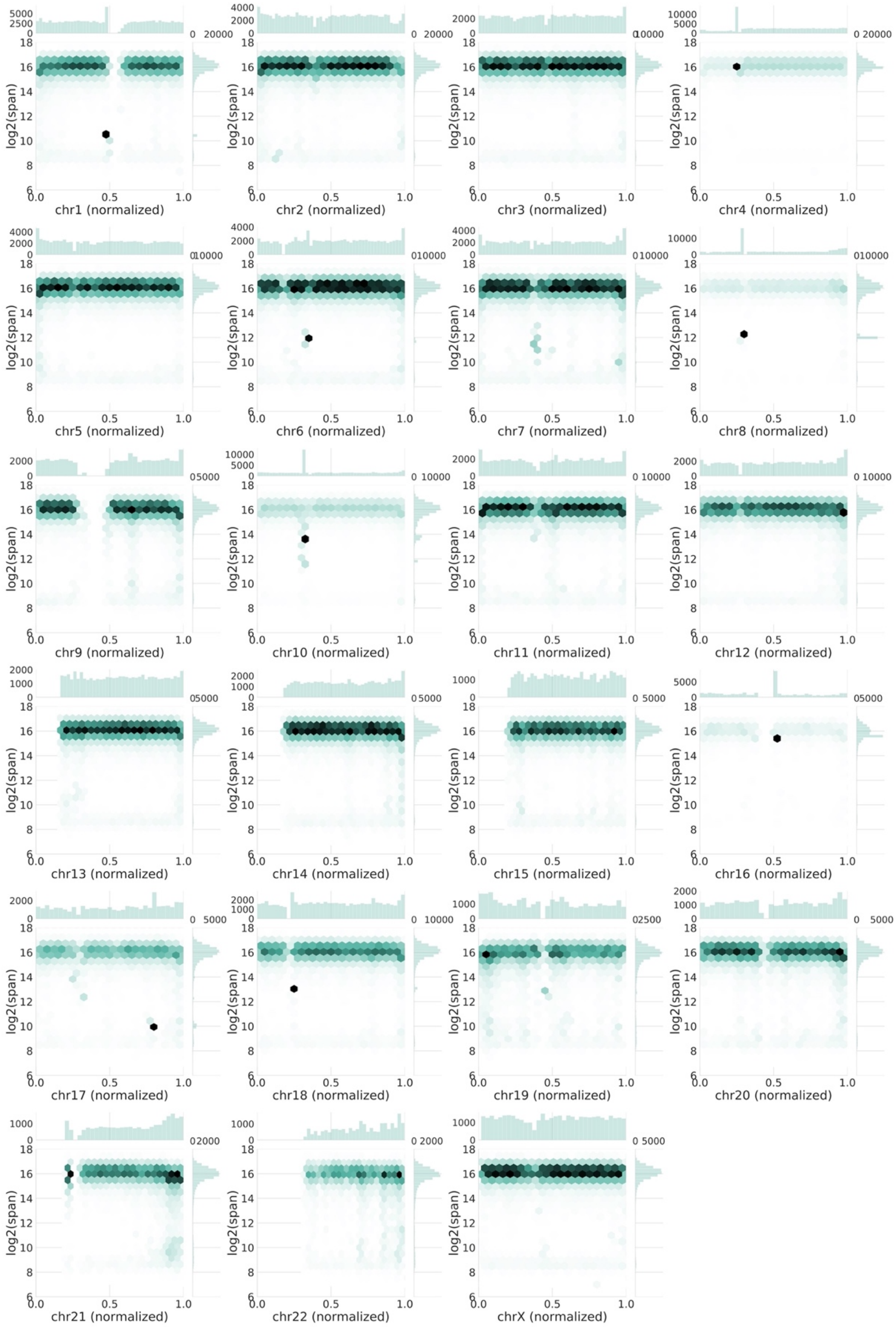

Supplementary Figure 24 | Distribution of 10x linked-reads barcode spanning size in sample T017.

Distribution of spanning size of 10x sequencing barcodes was displayed along the twenty-three chromosomes. Y axis shows the log under 2 of spanning size of barcodes, and the X axis shows the normalized (0.0-1.0) positions of chromosome. As the SCCC samples are all from women, the Y chromosome was ignored.

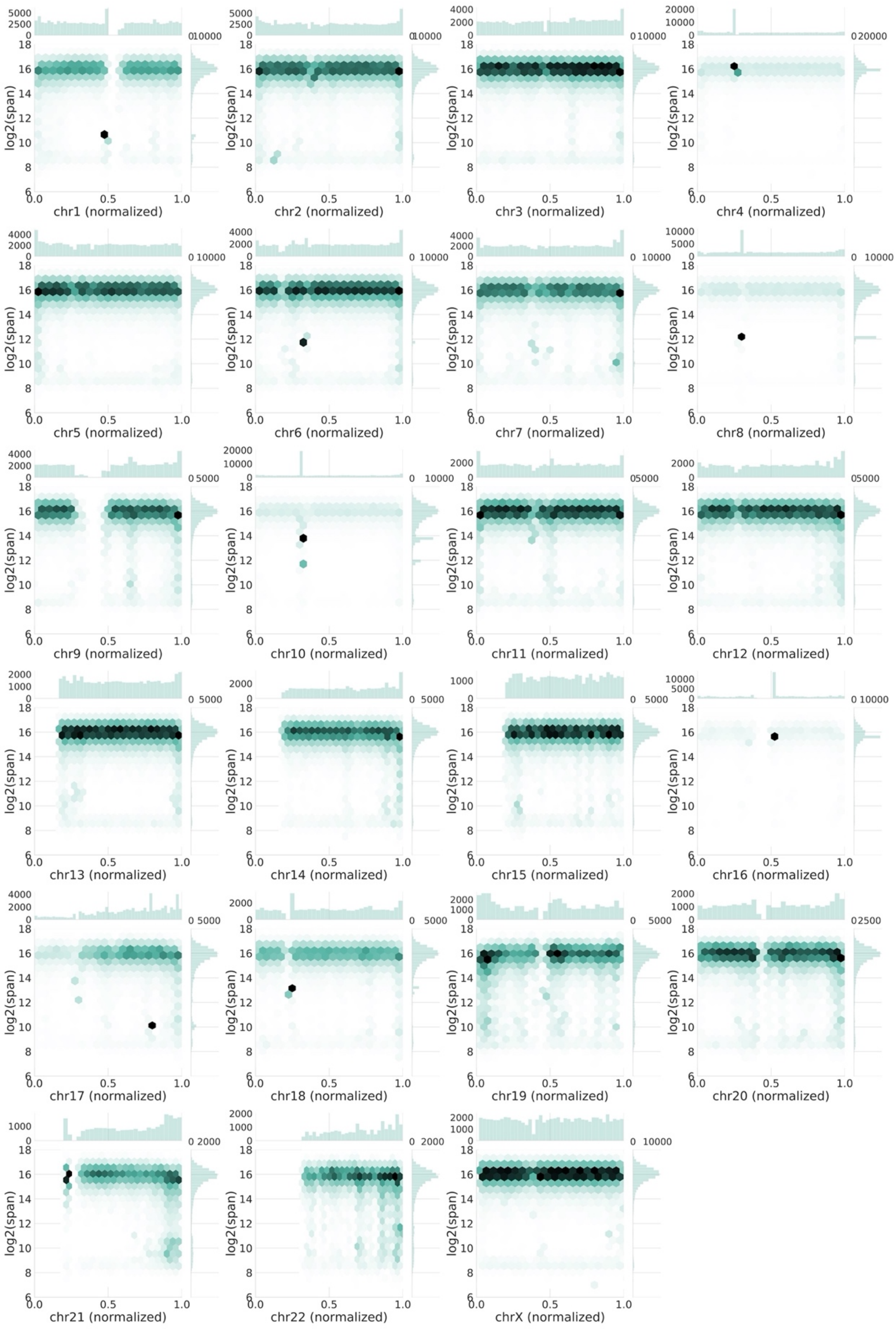

Supplementary Figure 25 | Distribution of 10x linked-reads barcode spanning size in sample T018.

Distribution of spanning size of 10x sequencing barcodes was displayed along the twenty-three

chromosomes. Y axis shows the log under 2 of spanning size of barcodes, and the X axis shows the normalized (0.0-1.0) positions of chromosome. As the SCCC samples are all from women, the Y chromosome was ignored.

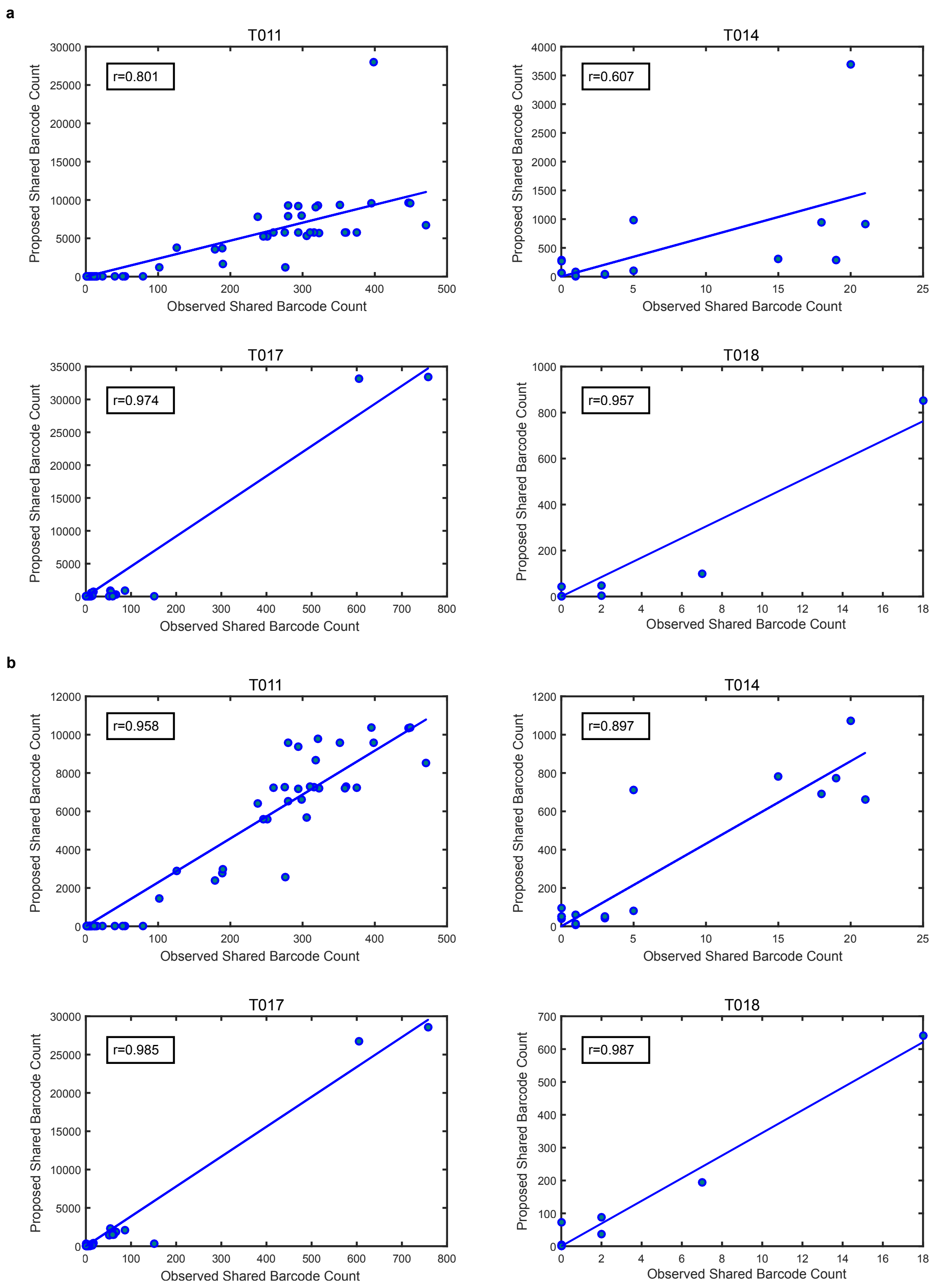

Supplementary Figure 26 | Correlation of 10x linked-reads and local haplotypes of four samples T011, T014, T017, and T018.

Pearson correlation ratio between numbers of shared real and simulated 10x-barcodes of each pair-wise anchors in Simplest (**a**) and Random-Best (**b**) Local Haplotypes of four SCCC samples (T011, T014, T017, and T018, see Supplementary Notes 10 and 13).

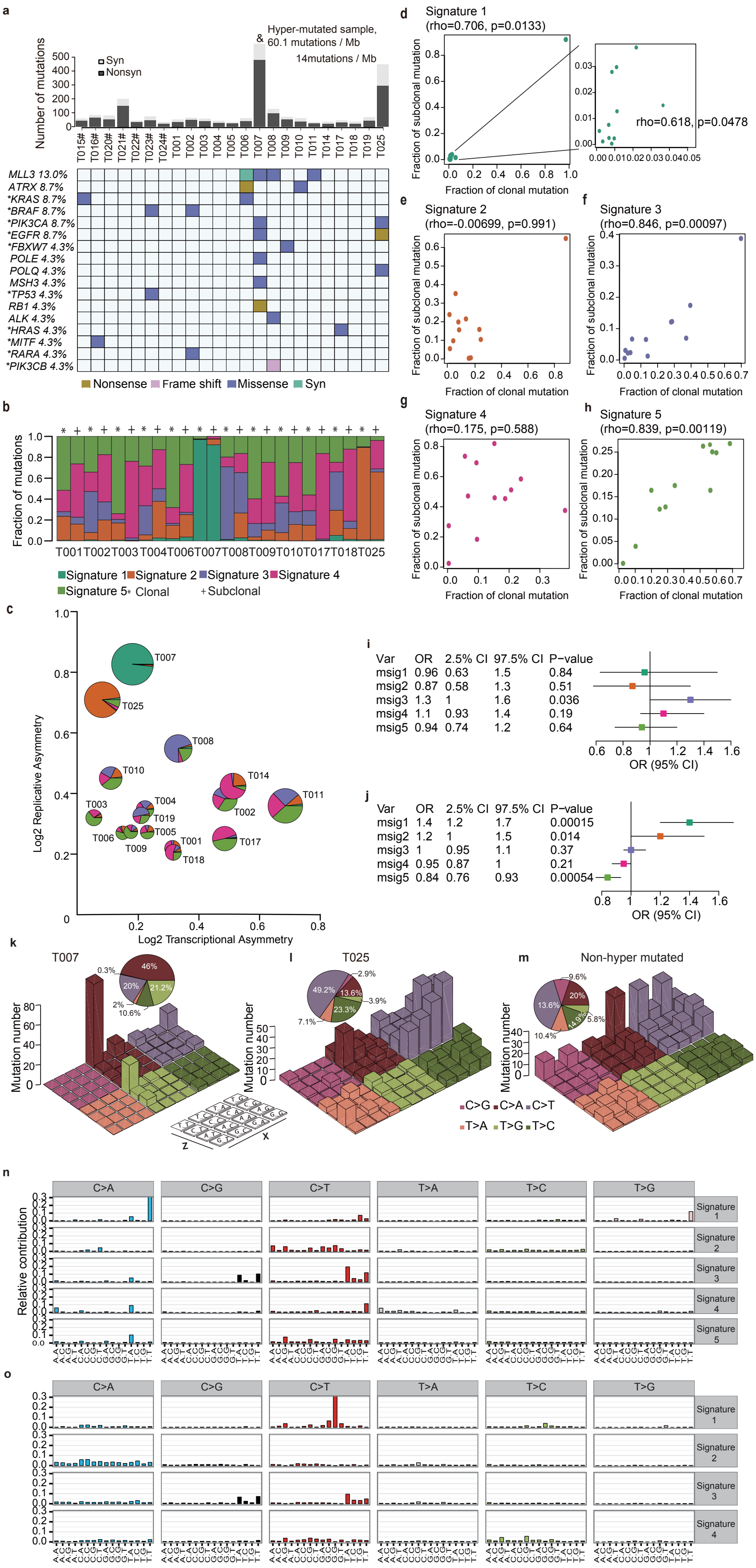

a
Number of mutations
Syn
Nonsyn
Hyper-mutated sample,
60.1 mutations / Mb
14mutations / Mb
T015# T016# T020# T021# T022# T023# T024# T001 T002 T003 T004 T005 T006 T007 T008 T009 T010 T011 T014 T017 T018 T019 T025
MLL3 13.0%
ATRX 8.7%
*KRAS 8.7%
*BRAF 8.7%
*PIK3CA 8.7%
*EGFR 8.7%
*FBXW7 4.3%
POLE 4.3%
POLQ 4.3%
MSH3 4.3%
*TP53 4.3%
RB1 4.3%
ALK 4.3%
*HRAS 4.3%
*MITF 4.3%
*RARA 4.3%
*PIK3CB 4.3%
Nonsense
Frame shift
Missense
Syn
b
Fraction of mutations
T001 T002 T003 T004 T006 T007 T008 T009 T010 T017 T018 T025
Signature 1
Signature 2
Signature 3
Signature 4
Signature 5
* Clonal
+ Subclonal
c
Log2 Replicative Asymmetry
Log2 Transcriptional Asymmetry
T007 T025 T008 T010 T014 T011 T003 T004 T019 T002 T005 T006 T009 T001 T017 T018
d
Signature 1
(rho=0.706, p=0.0133)
rho=0.618, p=0.0478
Fraction of subclonal mutation
Fraction of clonal mutation
e
Signature 2
(rho=-0.00699, p=0.991)
f
Signature 3
(rho=0.846, p=0.00097)
g
Signature 4
(rho=0.175, p=0.588)
h
Signature 5
(rho=0.839, p=0.00119)
i
Var OR 2.5% CI 97.5% CI P-value
msig1 0.96 0.63 1.5 0.84
msig2 0.87 0.58 1.3 0.51
msig3 1.3 1 1.6 0.036
msig4 1.1 0.93 1.4 0.19
msig5 0.94 0.74 1.2 0.64
OR (95% CI)
j
Var OR 2.5% CI 97.5% CI P-value
msig1 1.4 1.2 1.7 0.00015
msig2 1.2 1 1.5 0.014
msig3 1 0.95 1.1 0.37
msig4 0.95 0.87 1 0.21
msig5 0.84 0.76 0.93 0.00054
OR (95% CI)
k
T007
Mutation number
l
T025
m
Non-hyper mutated
C>G
C>A
C>T
T>A
T>G
T>C
n
Relative contribution
C>A C>G C>T T>A T>C T>G
Signature 1
Signature 2
Signature 3
Signature 4
Signature 5
o
C>A C>G C>T T>A T>C T>G
Signature 1
Signature 2
Signature 3
Signature 4

Supplementary Figure 27 | Mutation and mutation signatures from WGS and WES analysis in SCCC samples.

a, Distribution of somatic mutations and predicted driver mutations observed across exomic regions of 23 SCCCs. **b**, Fraction of clonal and subclonal somatic mutations attributable to different mutational signatures in SCCCs with high neoplastic cellularity. **c**, Mutational asymmetry analysis in SCCC. Pie chart representation with respects to five signatures is displayed. The size of pie chart is proportional to transcriptional and/or replicative asymmetry. **d-h**, Scatter plots illustrating correlations between fractions of clonal and subclonal mutations for Signatures 1, 2, 3, 4 and 5, respectively. **i** and **j,** Correlation analysis of transcriptional and replicative asymmetries with mutational signatures with the cox proportional hazards model across 16 samples, respectively. The center square data markers indicated estimated hazard ratios, and the error bars represent 95% CIs. **k, l** and **m**, Lego plots of mutation patterns in hyper- (T007 and T025) and regular-mutated SCCCs. Single-nucleotide substitutions are divided into six categories with 16 surrounding flanking bases. Inset pie chart shows the proportion of 6 categories of mutation patterns. **n** and **o,** Mutational signatures extracted from SCCC and small cell lung cancer.

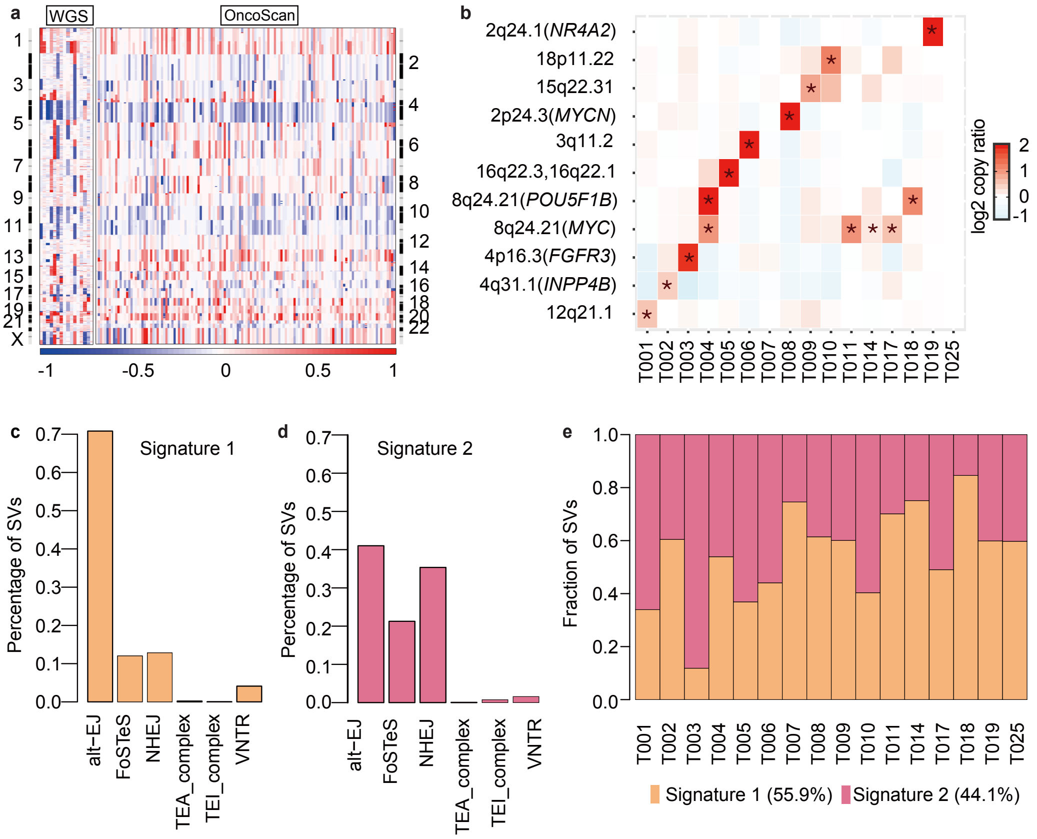

Supplementary Figure 28 | CNA landscape and SV signatures from WGS analysis in SCCC samples.

a, Copy number alteration landscapes obtained from whole-genome sequencing (left-panel) and OncoScan profiling (right-panel). **b**, Heatmap of copy number alterations around viral integration sites across different SCCCs. Asterisk represents viral integration event was identified in a specific genomic region and sample. **c** and **d,** Two structural variant signatures extracted from SCCC. **e**, Contribution of structural variant signatures 1 and 2 in 16 SCCC samples.

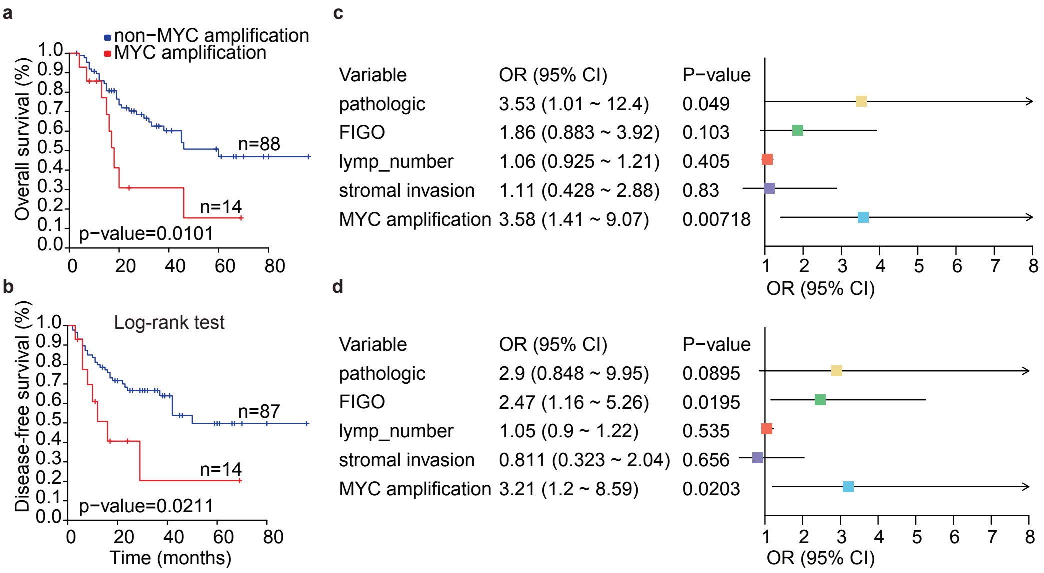

Supplementary Figure 29 | Survival analyses stratified by *MYC* amplification in SCCC samples.

a and **b,** Kaplan-Meier analysis of overall and disease free survival rates stratified by oncogene *MYC* amplification for 132 SCCC samples. Log-rank test is used to access significance of survival curves (*P*=0.010 for overall survival rate and *P*=0.021 for disease-free survival rate, respectively). **c** and **d,** Multivariate Cox analysis clinical variables and *MYC* amplification for overall and disease free survival rates with the cox proportional hazards model for 132 SCCC samples, respectively. The center square data markers indicated estimated hazard ratios, and the error bars represent 95% CIs.

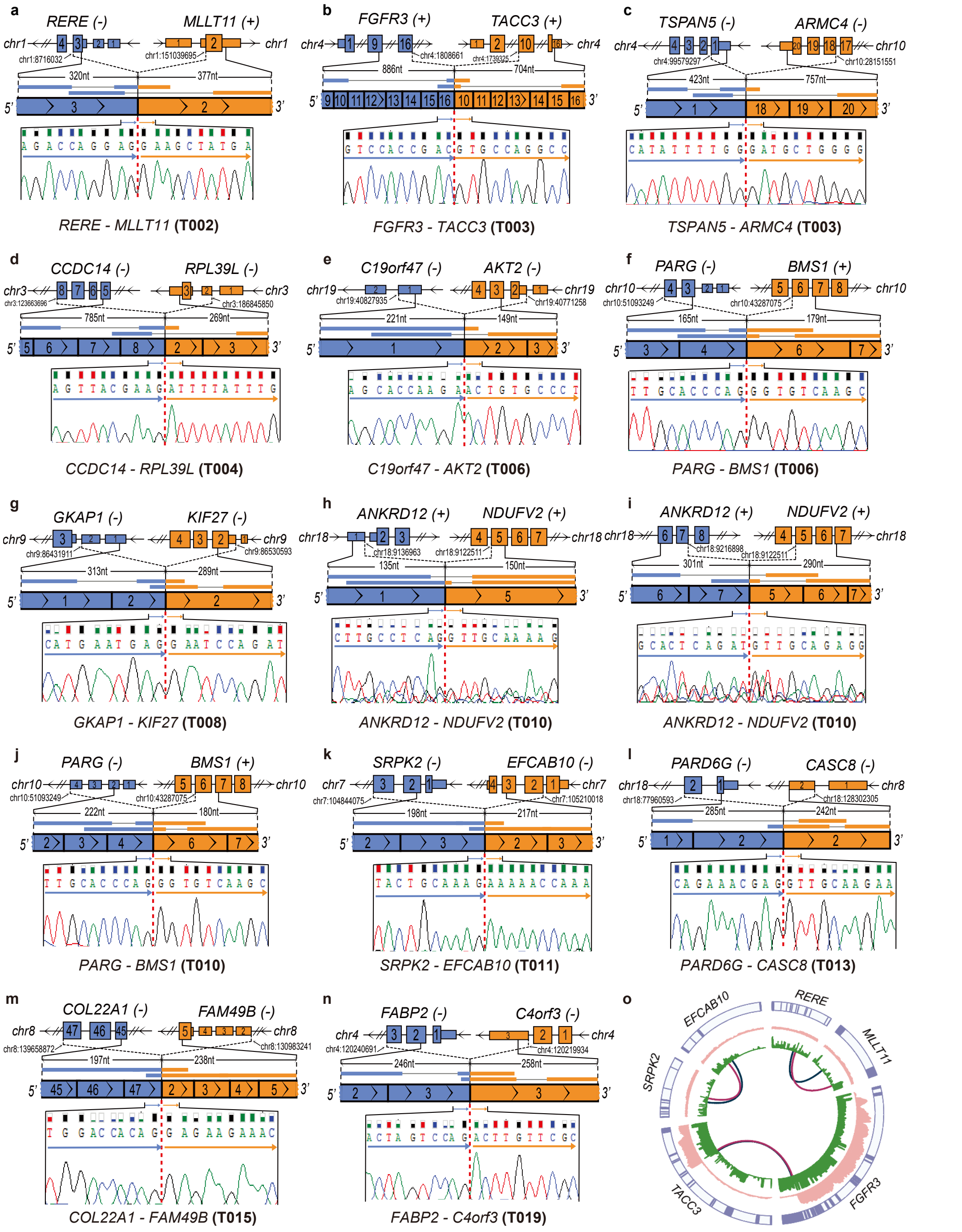

Supplementary Figure 30 | Fusion events cartoons with validated sequences in SCCC samples.

Fusion events that indicate 14 potential chromosomal rearrangements, including potential inversion and intra-chromosomal translocations in 10 SCCC tumor samples. **a-n**, Blue

segments are upstream genes, and downstream genes are in orange. Gene symbols are followed with their DNA strands. Exons around the junction sites are drawn with a double slash indicating exons that are not shown. The start positions of upstream genes and end positions of downstream genes are noted with a colon separating chromosomal location and reference genome coordinate. The span-reads and junction-reads from RNA-seq are shown over and under the junction sequences, respectively. Sanger sequencing of junction sequences are displayed under the junction sites. **o,** Three somatic structural variants identified from WGS data were shown.

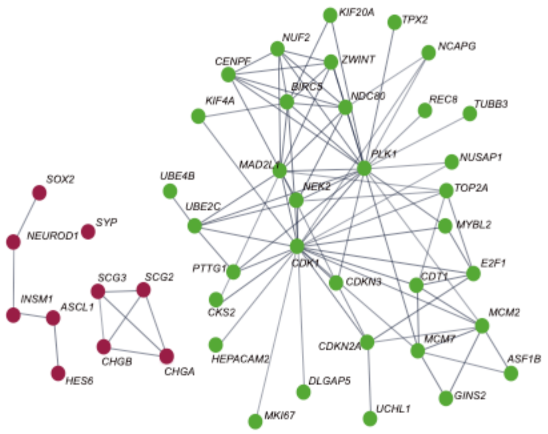

Supplementary Figure 31 | STRING analyses of DEGs in SCCC samples.

The interaction network of the top 100 significant DEGs was predicated by STRING (from string-db.org). Two distinct modules (cell cycle and neuroendocrine genes) were identified.

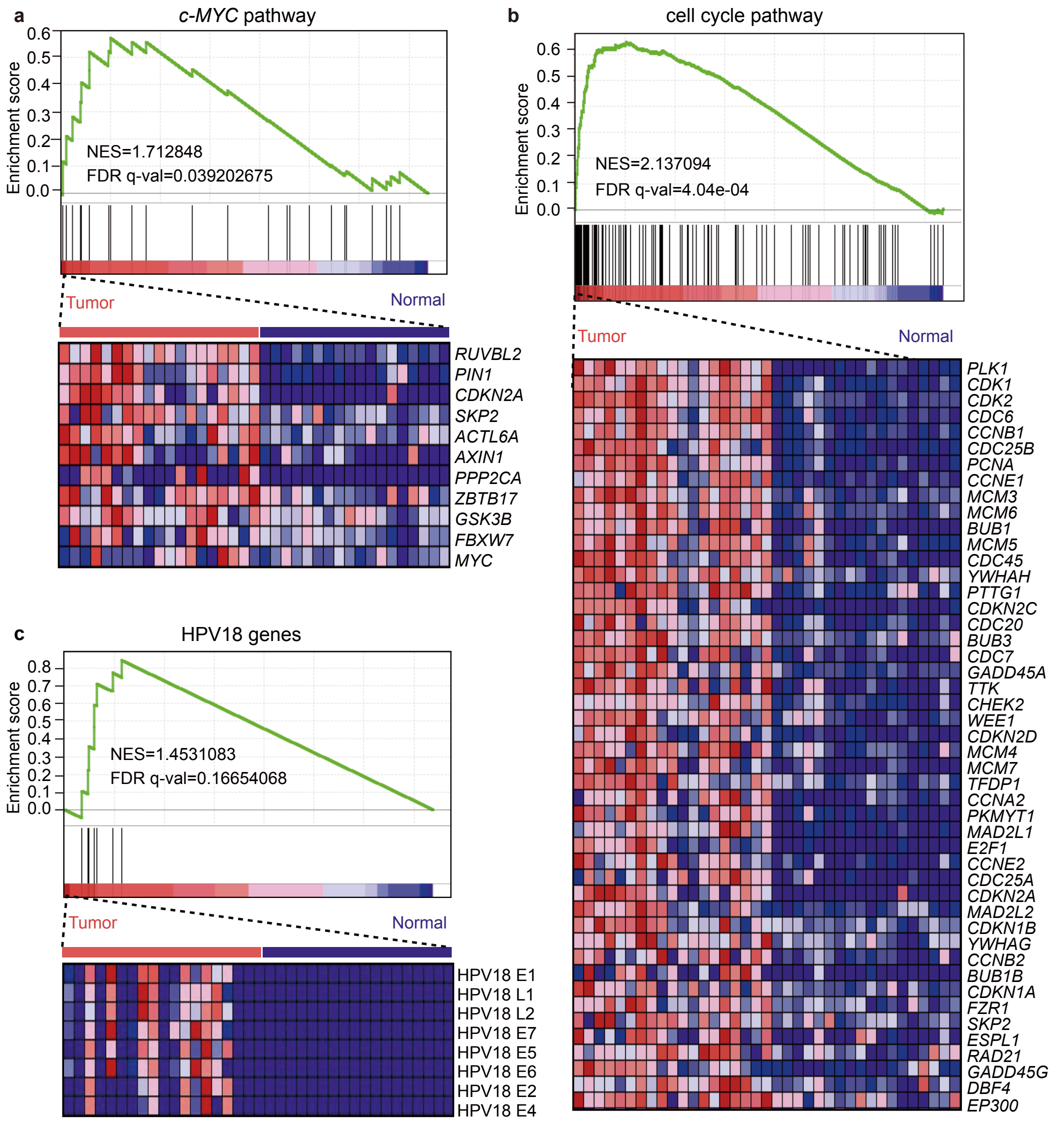

Supplementary Figure 32 | GSEA analyses of DEGs in SCCC samples.

a, **b** and **c,** GSEA analyses revealed enriched pathways of cell cycle, *c-MYC* and HPV 18 viral genes.

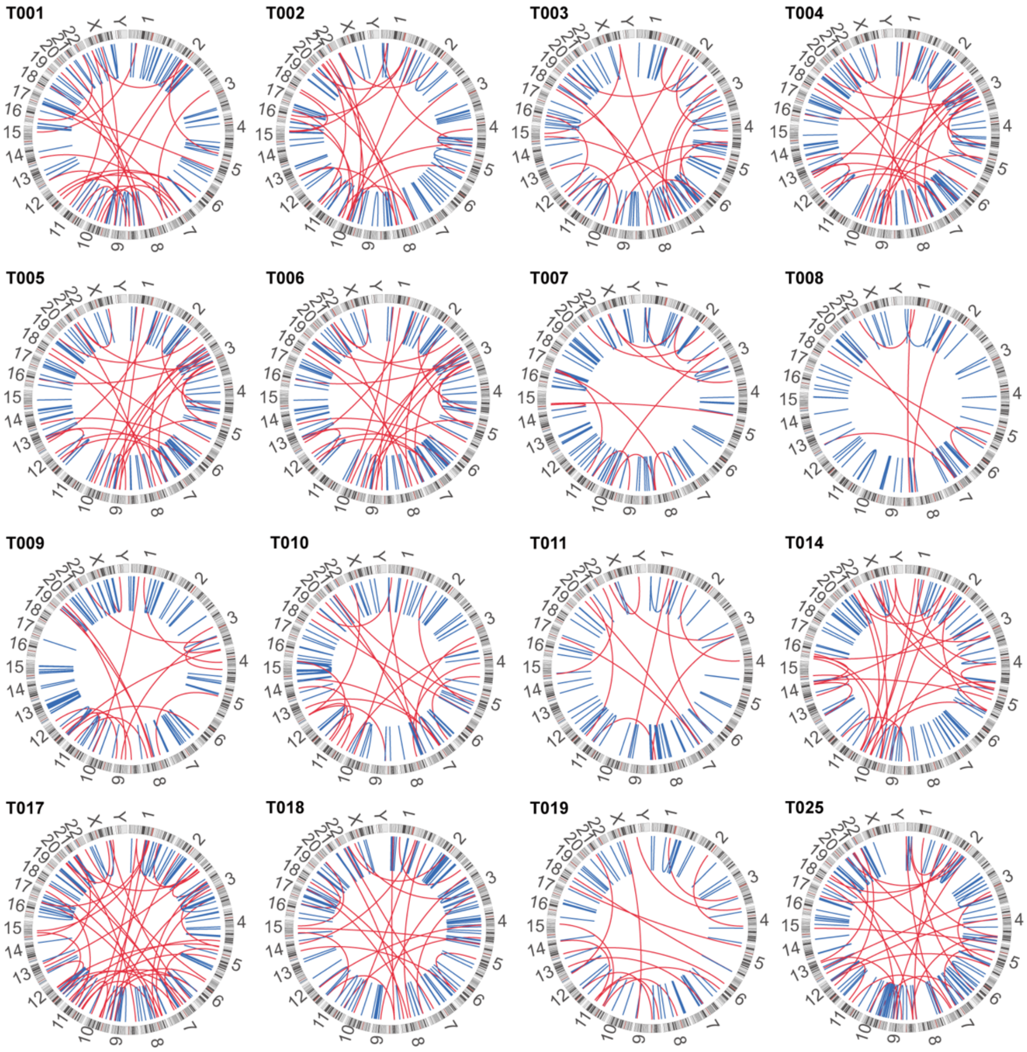

Supplementary Figure 33 | Structural variants from WGS analysis in SCCC samples.

Circos plots representation of structural variants obtained from whole genome sequencing in 16 SCCC tumors. Intra- and inter-chromosome rearrangements are shown in blue and red lines, respectively.

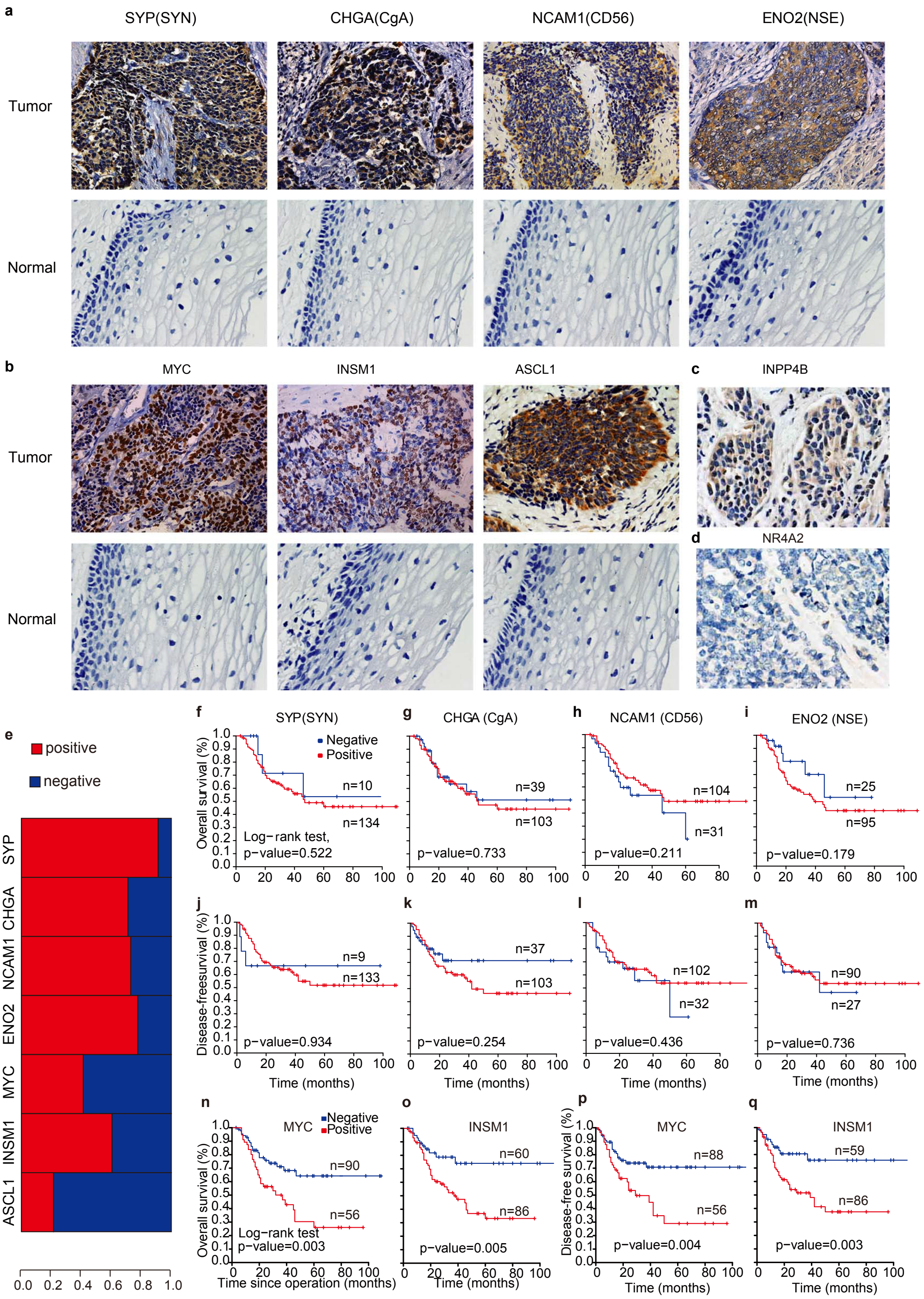

Supplementary Figure 34 | Immunohistochemical staining in SCCC FFPE samples.

a-d, Representative immunohistochemical staining images of canonical neuroendocrine markers (*SYP*, *CHGA*, *NCAM1* and *ENO2*), *MYC*, *INSM*, *ASCL1, INPP4B and NR4A2* (400×). **e**, The negative and positive percentages of immunohistochemical staining for

neuroendocrine markers, *MYC*, *INSM1* and *ASCL1.* **f-m**, Kaplan-Meier analysis of overall survival rates stratified by neuroendocrine markers (*SYP*, *CHGA*, *NCAM1* and *ENO2*) for 132 SCCC samples, respectively. Log-rank test is used to access significance of survival curves (*P*=0.522, *P*= 0.733, *P*=0.211 and *P*=0.179 for overall survival rate, and *P*=0.934, *P*=0.254, *P*=0.436, *P*=0.736 for disease-free survival rate, respectively). **n-q**, Kaplan-Meier analysis of overall and disease free survival rates for two subgroups stratified by *MYC* (*P*=0.003 and *P*=0.004) or *INSM1* (*P*=0.005 and *P*=0.003) protein expression, respectively.

Supplementary Table 1. Clinical statistics in SCCC patients.

1. "NACT" stands for Neoadjuvant chemotherapy.

	Total samples		NGS samples	
Variables	No.	%	No.	%
Age (yrs)				
< 40	72	34%	4	16%
≥ 40	142	66%	21	84%
Unknown	0	0%	0	0%
FIGO stage				
IA–IB	135	63%	19	76%
IIA–IV	68	32%	6	24%
Unknown	11	5%	0	0%
Tumor homology				
Pure	180	84%	23	92%
Mixed	34	16%	2	8%
Unknown	0	0%	0	0%
Tumor size				
< 4cm	80	37%	11	44%
≥ 4cm	62	29%	11	44%
Unknown	72	34%	3	12%
Lymph node involvement				
Yes	83	39%	6	24%
No	116	54%	17	68%
Unknown	15	7%	2	8%
Depth of cervical stromal invasion				
<1/2	43	20%	4	16%
≥1/2	120	56%	18	72%
Unknown	51	24%	3	12%
Vaginal stump invasion				
Yes	8	4%	0	0%
No	185	86%	23	92%
Unknown	21	10%	2	8%
Lymphovascular space invasion				
Yes	90	42%	2	8%
No	41	19%	9	36%
Unknown	83	39%	14	56%
NACT				
Yes	67	31%	7	28%
No	136	64%	18	72%
Unknown	11	5%	0	0%
HPV typing				
HPV16	14	7%	1	4%
HPV18	126	59%	17	68%
HPV18 and HPV16	66	31%	7	28%
Negative	2	1%	0	0%

Unknown	6	3%	0	0%
SYN expression				
Positive	191	89%	22	88%
Negative	19	9%	2	8%
Unknown	4	2%	1	4%
CgA expression				
Positive	146	68%	18	72%
Negative	59	28%	6	24%
Unknown	9	4%	1	4%
CD56 expression				
Positive	135	63%	22	88%
Negative	49	23%	2	8%
Unknown	30	14%	1	4%
NSE expression				
Positive	128	60%	15	60%
Negative	37	17%	7	28%
Unknown	49	23%	3	12%

Supplementary Table 2. VCS data summary.

Sample ID	Read Length(bp)	Clean Reads	Clean Bases (bp)	Q20 percent	GC percent
T012	142;142	5336876	753879342	92.79%	42.53%
T013	142;142	3126828	430854111	96.85%	41.94%
T015	142;142	1320900	183060622	96.10%	41.04%
T016	142;142	7431368	1037994593	94.61%	42.07%
T020	142;142	3260004	459691095	91.41%	42.56%
T022	142;142	6637268	913976797	94.27%	38.40%
T023	142;142	6722066	930310245	93.01%	41.40%
T024	142;142	7082596	943757712	96.77%	41.87%
T028	142;142	7344514	987793314	95.36%	39.84%
T031	142;142	3103424	421767652	92.91%	43.83%
T032	142;142	4733924	632817817	96.99%	41.53%
T034	142;142	3852554	451868561	97.50%	41.96%
T035	142;142	8015098	1102621008	96.64%	41.07%
T036	142;142	5639580	736852267	96.30%	41.69%
T038	142;142	8874704	1049901568	98.25%	42.10%
T039	142;142	1504224	202247975	94.84%	43.35%
T040	142;142	4205468	554392286	96.18%	40.53%
T043	142;142	4245584	572958351	96.33%	41.70%
T045	142;142	936498	125263453	96.38%	41.57%
T046	142;142	24689832	3428406429	96.13%	41.86%
T047	142;142	14711046	1693192562	96.75%	39.33%
T048	142;142	1044194	111644499	96.72%	51.45%
T049	142;142	1690402	193242523	96.34%	39.34%
T051	142;142	1042136	132486162	95.07%	43.59%
T052	142;142	1813850	223377863	95.70%	36.16%
T054	142;142	1599058	219717578	96.82%	39.69%
T055	142;142	940192	100941657	98.02%	41.95%
T056	142;142	3635878	493310945	96.16%	40.83%
T057	142;142	6960156	922907700	97.16%	38.92%
T058	142;142	4900792	680084501	91.99%	44.84%
T059	142;142	692170	95174500	95.13%	44.77%
T060	142;142	15589100	2036147984	96.78%	39.06%
T061	142;142	21455552	2954043201	96.32%	41.53%
T062	142;142	39672858	5143112208	96.67%	41.24%
T063	142;142	698674	96569765	94.91%	40.02%
T064	142;142	2178408	305191578	95.27%	40.86%
T065	142;142	10112448	1380918979	95.39%	42.27%
T066	142;142	301398	41131169	94.37%	41.09%
T067	142;142	3833434	535846421	92.98%	41.45%
T068	142;142	1276002	178432189	94.96%	40.64%
T070	142;142	34982628	4821323263	96.77%	39.19%
T071	142;142	833934	115072268	93.33%	44.70%
T072	142;142	1072752	149464719	94.00%	42.04%
T073	142;142	1052758	145196797	93.17%	43.87%
T074	142;142	1761154	246078937	96.08%	40.93%
T076	142;142	11184534	1499039545	96.70%	40.66%
T077	142;142	801898	110400712	93.88%	40.63%
T078	142;142	8367668	1127721759	95.84%	39.93%
T081	142;142	24723414	3428505436	95.28%	39.23%
T082	142;142	2414760	312677401	94.01%	44.04%
T083	142;142	8554876	1199493706	96.16%	40.70%

T084	142;142	1134828	147569909	96.74%	40.16%
T086	142;142	904604	117297296	97.11%	41.10%
T087	142;142	2463774	332375746	97.19%	39.02%
T088	142;142	6492742	876650247	96.05%	41.52%
T089	142;142	6480	900554	95.75%	41.48%
T090	142;142	965860	134046366	92.67%	41.28%
T092	142;142	25401654	3440055085	96.79%	41.19%
T093	142;142	6415558	858551302	94.82%	39.60%
T097	142;142	10163546	1363954843	96.94%	41.50%
T098	142;142	2057788	284165362	95.94%	41.85%
T099	142;142	8158828	1097443191	96.49%	39.94%
T100	142;142	7370354	979450318	96.26%	41.82%
T101	142;142	1944738	257806005	96.30%	38.80%
T102	142;142	2731116	380004581	95.72%	40.79%
T103	142;142	36217324	4964955614	95.72%	40.68%
T104	142;142	44046892	5807093689	96.35%	41.93%
T105	142;142	8530856	1184629434	94.61%	39.49%
T106	142;142	3061548	390798556	97.63%	39.76%
T107	142;142	8799974	1207616961	95.94%	42.64%
T108	142;142	15284298	1917434764	97.40%	40.36%
T109	142;142	1805214	245393046	95.62%	39.11%
T110	142;142	12516402	1672847541	96.86%	38.77%
T113	142;142	2873622	392197593	93.69%	39.23%
T115	142;142	1498162	201549855	96.19%	41.53%
T116	142;142	1169128	151899005	95.42%	43.59%
T117	142;142	26936178	3661623430	97.09%	39.98%
T118	142;142	9183110	1239626315	93.61%	44.67%
T120	142;142	19214772	2488306028	97.43%	41.32%
T121	142;142	1120260	142230455	97.04%	39.72%
T124	142;142	2304082	287238720	97.67%	40.92%
T125	142;142	346088	44571873	97.21%	41.89%
T126	142;142	9879460	1303355925	96.88%	39.34%
T127	142;142	5444260	733956614	93.56%	44.88%
T128	142;142	3680198	492799171	96.27%	39.25%
T129	142;142	325130	44259510	95.81%	41.91%
T130	142;142	5352834	723510050	95.84%	41.06%
T131	142;142	4176318	515457096	97.76%	40.15%
T132	142;142	3800256	515654209	95.36%	40.31%
T134	142;142	1143542	149360758	96.91%	40.72%
T136	142;142	1531990	212840169	96.17%	40.18%
T138	142;142	9047464	1236984768	96.42%	42.04%
T140	142;142	7169544	996610973	95.66%	39.48%
T143	142;142	2460178	334441669	96.86%	41.36%
T145	142;142	3007536	421046547	95.78%	41.76%
T147	142;142	1988936	272276921	95.61%	41.67%
T148	142;142	19614472	2724039590	95.20%	40.70%
T149	142;142	44810648	6116277867	95.86%	39.33%
T151	142;142	1025508	141738318	96.25%	42.12%
T152	142;142	36651632	5092598566	95.99%	40.39%
T153	142;142	6520966	904550283	95.79%	41.24%
T154	142;142	1385838	189514088	91.40%	44.86%
T155	142;142	11532726	1539416153	96.16%	39.96%
T157	142;142	36954490	5086491849	96.53%	41.16%
T158	142;142	18009226	2340214148	96.17%	39.40%
T159	142;142	9238060	1252537915	93.52%	40.62%

T160	142;142	2134208	299325500	96.20%	38.14%
T161	142;142	7836842	1050808243	97.24%	39.30%
T162	142;142	2506438	350737400	88.85%	44.72%
T163	142;142	1879416	247723303	95.57%	37.44%
T164	142;142	11251036	1575379408	94.99%	40.97%
T165	142;142	12983386	1786502838	95.64%	39.87%
T166	142;142	30982168	4282991147	96.18%	40.62%
T167	142;142	1877684	256833152	94.75%	42.30%
T168	142;142	3483164	465544611	94.16%	38.62%
T169	142;142	3707686	510148009	95.02%	42.76%
T170	142;142	1720052	239687826	91.76%	39.49%
T171	142;142	15639092	2174828522	95.88%	40.04%
T172	142;142	14711870	2011969932	95.19%	41.77%
T173	142;142	5097056	642293641	96.20%	36.13%
T174	142;142	5135216	722826694	91.21%	43.25%
T175	142;142	11276216	1592080282	94.29%	41.02%
T176	142;142	1127998	154809810	95.85%	41.94%
T177	142;142	23319468	3267790405	95.21%	40.96%
T179	142;142	6179794	857512395	94.30%	42.77%
T180	142;142	16816336	2351039437	93.00%	42.29%
T181	142;142	16904774	2337370944	95.60%	40.66%
T182	142;142	4064720	568019294	89.94%	43.81%
T184	142;142	38032082	5299031028	95.79%	39.76%
T185	142;142	1152126	160101699	87.27%	40.97%
T186	142;142	914858	127005257	94.30%	41.00%
T187	142;142	16769356	2339612324	94.63%	37.92%
T188	142;142	6285358	875505117	95.49%	39.00%
T190	142;142	6968834	971166939	95.55%	38.39%
T191	142;142	11806704	1650596241	95.52%	38.37%
T192	142;142	11521046	1517991655	95.20%	40.53%
T193	142;142	4398818	611480769	92.64%	45.70%
T194	142;142	6912298	974806048	93.81%	41.21%
T195	142;142	965848	123673020	91.72%	45.13%
T198	142;142	16785988	2339344974	94.88%	41.26%
T199	142;142	2760454	353040736	97.06%	41.05%
T202	142;142	4604850	639741657	94.31%	41.92%
T203	142;142	10065334	1413202679	93.78%	41.87%
T205	142;142	8264262	1116896865	96.23%	38.35%
T207	142;142	13265688	1830026234	96.34%	40.65%
T210	142;142	8298330	1092975875	96.74%	37.92%
T211	142;142	16805016	2315228297	96.06%	38.60%
T212	142;142	25384932	3316049076	96.63%	39.88%
T213	142;142	18908268	2636354637	95.57%	41.14%
T214	142;142	8691874	1206136360	95.37%	41.44%

Supplementary Table 3. RNA-seq data summary.

Sample Name	Read length(bp)	Clean Data Size(bp)	Read1 GC(%)	Read2 GC(%)	Read1 Q20(%)	Read2 Q20(%)	Read1 Q30(%)	Read2 Q30(%)	Alignment rate(%)
T001	125	6485567500	51.96	52.08	96.22	95.07	92.17	90.41	95.19
T002	125	6257892000	51.63	51.71	96.43	95.55	92.51	91.21	95.49
T003	125	7390759500	51.72	51.84	96.16	95.07	92.04	90.42	95.32
T004	125	6329432750	51.81	51.91	96.52	95.26	92.70	90.71	95.35
T005	125	7200161500	52.14	52.24	96.59	95.70	92.85	91.55	95.33
T006	125	7371405500	51.38	51.46	96.74	95.92	93.14	91.95	96.10
T007	125	6410075250	51.78	51.91	96.47	95.50	92.65	91.20	95.56
T008	125	6962726000	52.33	52.45	96.75	95.17	93.15	90.64	92.65
T010	125	7180987750	45.21	45.08	94.62	92.79	89.37	86.73	91.75
T011	125	6816119750	47.92	47.96	96.08	94.31	92.04	89.40	94.86
T012	125	6519871500	47.12	47.16	95.77	94.01	91.51	88.93	94.78
T013	125	6606137000	48.61	48.67	95.71	93.95	91.34	88.73	93.85
T014	125	6800247000	48.53	48.58	95.89	94.59	91.63	89.80	94.84
T015	125	7053292000	47.45	47.50	95.98	94.35	91.82	89.45	94.04
T016	125	6920646000	47.43	47.49	95.63	93.90	91.28	88.74	94.73
T017	125	6707563500	48.54	48.58	95.64	94.29	91.18	89.33	94.28
T018	125	7070678500	47.38	47.48	95.12	94.11	90.39	89.09	91.00
T019	125	7432042000	48.76	48.90	96.30	94.21	92.32	88.78	95.80
T025	125	7229282750	51.74	51.82	96.29	95.39	92.27	90.94	96.01
C001	125	7379956500	47.99	48.02	93.87	91.43	87.58	83.88	95.19
C002	125	7506357000	48.97	49.01	94.06	91.52	87.86	83.95	96.19
C003	125	8605399500	47.63	47.71	95.21	93.55	90.35	87.74	95.64
C004	125	8993115500	48.26	48.29	95.14	93.42	90.18	87.53	96.01
C005	125	8360773500	48.44	48.49	95.76	93.99	91.29	88.50	96.60
C006	125	8172986000	48.38	48.45	95.85	94.23	91.47	88.91	96.28
C007	125	7821674500	47.21	47.33	95.93	94.91	91.85	90.33	95.22
C008	125	7168929000	48.27	48.35	96.33	94.70	92.49	89.92	96.26
C009	125	9134940750	48.37	48.42	95.47	91.93	90.88	85.23	92.84
C010	125	7817689500	49.42	49.49	95.29	91.80	90.47	84.95	94.30

C011	125	8163888750	47.54	47.59	95.25	92.41	90.49	86.07	93.22
C012	125	8799636500	50.28	50.40	96.16	94.92	92.09	90.14	94.76
C013	125	7624705500	50.95	51.03	96.29	94.95	92.32	90.19	88.87
C014	125	7905688500	51.34	51.43	95.95	94.85	91.71	90.02	82.52
C015	125	8084953500	52.21	52.30	95.98	94.59	91.71	89.55	90.59
C016	125	8810362750	47.25	47.32	96.07	94.45	92.05	89.60	95.26
C017	125	9317570750	46.66	46.73	95.98	94.70	91.90	90.00	94.11
C018	125	9698973500	47.35	47.42	96.17	94.84	92.22	90.22	93.81

Supplementary Table 4. 10X long-range sequencing data summary.

Sample ID	Clean Reads	Clean Bases (bp)
T011	1169726334	162007097259
T014	1177626192	163101227592
T017	1168360534	161817933959
T018	1160355308	160709210158

Sample ID	Mapped Reads	Mapped Bases (bp)	Mapping Rate	Uniq Mapped Reads	Uniq Mapped Bases(bp)	Uniq Mapped rate
T011	1094023672	151480328918	93.53%	1014847273	140484295406	92.76%
T014	1162042565	160909020404	98.68%	1028484704	142337919925	88.51%
T017	1156742438	160168442308	99.01%	1074593116	148754839995	92.90%
T018	1150088031	159249572930	99.12%	1055562413	146114451715	91.78%

Sample ID	Average Depth (X)	Coverage			
		>=1X	>=4X	>=10X	>=20X
T011	37.53	99.15%	98.72%	97.86%	93.31%
T014	34.91	99.14%	98.61%	97.46%	84.74%
T017	39.66	99.17%	98.77%	97.70%	91.57%
T018	38.11	99.14%	98.70%	97.81%	92.81%

Sample ID	Available Barcodes		NA Barcodes
	Count	PE-reads	PE-reads
T011	3507623	553962346	30900821
T014	3570658	551934053	36879043
T017	3520274	552870608	31309659
T018	3542389	548246588	31931066

Supplementary Table 5. HPV18 gene expression in RNA-seq analysis.

Gene symbol	T001	T002	T003	T004	T005	T006	T007	T008
E6	18.816	10.639	0.000	43.734	0.000	220.903	0.000	4.235
E7	21.356	8.512	0.000	42.552	0.000	259.481	0.217	4.340
E1	3.440	1.136	0.000	17.700	0.018	41.801	0.062	0.699
E2	0.073	0.090	0.000	18.593	0.000	0.230	0.000	0.000
E4	0.139	0.000	0.000	55.815	0.000	0.000	0.000	0.000
E5	0.000	1.361	0.000	56.267	0.000	19.794	0.000	0.000

Gene symbol	T010	T011	T012	T013	T014	T015	T016	T017
E6	27.858	61.305	0.103	8.012	16.426	196.451	58.753	32.113
E7	29.980	94.221	0.143	23.374	19.222	239.883	72.143	38.498
E1	25.244	26.567	0.023	0.404	20.814	1.647	17.750	4.394
E2	2.651	7.541	0.000	0.467	19.619	1.639	65.017	4.290
E4	4.521	19.281	0.000	1.922	66.396	6.289	232.138	2.059
E5	60.911	16.192	0.091	1.270	39.061	5.032	152.731	6.129

Gene symbol	T018	T019	T025	H001	H002	H003	H004	H005
E6	2.892	0.000	0.000	0.000	0.000	0.000	0.000	0.000
E7	4.589	0.000	0.000	0.000	0.000	0.000	0.000	0.000
E1	5.276	0.000	0.000	0.000	0.000	0.000	0.000	0.000
E2	22.042	0.000	0.000	0.000	0.000	0.000	0.000	0.000
E4	74.581	0.000	0.000	0.000	0.000	0.000	0.000	0.000
E5	64.125	0.000	0.000	0.000	0.000	0.000	0.000	0.000

Gene symbol	H006	H007	H008	H009	H010	H011	H012	H013
E6	0.000	0.000	0.000	0.047	0.216	0.000	0.000	0.000
E7	0.000	0.000	0.000	0.085	0.000	0.000	0.000	0.000
E1	0.000	0.000	0.000	0.135	0.058	0.025	0.000	0.000
E2	0.000	0.000	0.000	0.013	0.000	0.000	0.000	0.000
E4	0.000	0.000	0.000	0.000	0.000	0.000	0.000	0.000
E5	0.000	0.000	0.000	0.000	0.000	0.000	0.000	0.000

Gene symbol	H014	H015	H016	H017	H018
E6	0.000	0.000	0.000	0.000	0.000
E7	0.000	0.000	0.000	0.000	0.000
E1	0.000	0.000	0.000	0.000	0.000
E2	0.000	0.000	0.000	0.000	0.000
E4	0.000	0.000	0.000	0.000	0.000
E5	0.000	0.000	0.000	0.000	0.000

Supplementary Table 6. RNA and protein expression of MYC, INSM1 and ASCL1.

		MYC			INSM1		ASCL1	
**Sample ID**	**HPV integration-MYC/MCYN**	**CNV-MYC**	**RNA expression （PFKM）**	**protein expression (IHC)**	**RNA expression （PFKM）**	**protein expression (IHC)**	**RNA expression （PFKM）**	**protein expression (IHC)**
T001	no	-0.077	5.3	–	359.5	+	17.8	+
T002	no	0.073	9.2	+	113.6	+	232.3	+
T003	no	0.012	4.3	–	21.3	+	0.6	+
T004	MYC	2.143	134	+	669.5	+	224	–
T005	no	0.001	9.9	+	39.2	+	172.9	+
T006	no	-0.113	9.1	+	200.9	+	469.9	+
T007	no	-0.03	9.8	+	16.9	–	10.1	–
T008	MYCN	-0.578	10.1	–	114.2	+	0.4	–
T009	no	0.09	NA	–	NA	+	NA	–
T010	no	0.188	0.1	–	22.7	+	0.3	–
T011	MYC	2.153	34.7	+	70.3	+	1.3	–
T012	no	NA	20.7	–	0.1	–	1.6	+
T013	MYC	NA	43.4	+	39.2	+	7	+
T014	MYC	0.423	60.1	+	107	–	291.3	–
T015	no	NA	3.5	–	44.2	+	209.3	–
T016	no	NA	1.2	+	88.2	+	162.4	–
T017	MYC	1.018	69.1	–	151.9	–	2.1	–
T018	MYC	0.071	57.1	+	60.7	+	229.7	–
T019	no	0.01	27.9	–	28.8	+	0	–
T025	no	-0.02	9	–	0.3	+	0.1	+
normal mean			14.2		0		0.3	

Supplementary Table 7. The 5-year OS rates of SCCC patients whose HPV integration sites were located on genes or gene families than those without HPV integration.

GeneSymbol and GeneGroup	sample with HPV integration			sample without HPV integration			Log-rank (Mantel-Cox) test		Gehan-Breslow-Wilcoxon test	
	total	loss to follow-up	effective sample to count	total	loss to follow-up	effective sample to count	Chi square	*P* value	Chi square	*P* value
MYC	30	9	21	56	15	41	0.002039	0.9640	0.04428	0.8333
MYC_family	36	12	24	51	12	39	0.004569	0.9461	0.007194	0.9324
SOX_family	8	1	7	73	23	50	1.878	0.1706	0.7729	0.3793
NR4A_family	6	3	3	76	21	55	6.772	0.0093	5.298	0.0213
ANKRD_family	6	2	4	75	22	53	2.656	0.1031	2.003	0.157
CEA_family	3	2	1	78	22	56	0.3883	0.5332	0.3682	0.544
SangerCancerCensus-others	42	15	27	39	9	30	0.4752	0.4906	0.3912	0.5317
CancerDependencyMap-others	36	9	27	45	15	30	1.434	0.2311	2.365	0.1241
HasAtLeastTwoGroups	40	15	25	41	9	32	0.167	0.6828	0.06832	0.7938

Supplementary Table 8. The 5-year DFS rates of SCCC patients whose HPV integration sites were located on genes or gene families than those without HPV integration.

GeneSymbol and GeneGroup	sample with HPV integration			sample without HPV integration			Log-rank (Mantel-Cox) test		Gehan-Breslow-Wilcoxon test	
	total	loss to follow-up	effective sample to count	total	loss to follow-up	effective sample to count	Chi square	*P* value	Chi square	*P* value
MYC	30	10	20	56	17	39	0.002017	0.9642	0.09791	0.7543
MYC_family	36	13	23	51	14	37	0.01106	0.9162	0.003318	0.9541
SOX_family	8	2	6	73	25	48	3.271	0.0705	2.775	0.0958
NR4A_family	6	4	2	76	24	52	8.869	0.0029	8.731	0.0031
ANKRD_family	6	2	4	75	25	50	2.102	0.1471	1.753	0.1854
CEA_family	3	2	1	78	25	53	0.3384	0.5608	0.3238	0.5694
SangerCancerCensus-others	42	17	25	39	10	29	1.167	0.2801	1.034	0.3091
CancerDependencyMap-others	36	12	24	45	15	30	0.02612	0.8716	0.2916	0.5892
HasAtLeastTwoGroups	40	18	22	41	9	32	1.645	0.1997	1.727	0.1888

Supplementary Table 9. The immunohistochemical staining of gene families with HPV integration.

GeneGroup	Groups	GeneSymbol	number of tissue sections	Total number of tissue sections	mean value	standard deviation	95% confidence interval	*p*-value(Mann-Whitney U test)
MYC_family	Integration	*MYC*	29	34	1.941	3.36597	(0.767,3.116)	0.934
		MYC -N	2					
		MYC -L	3					
	non-integration	*MYC*	50	69	1.667	2.95389	(0.957,2.376)	
		MYC -N	10					
		MYC -L	9					
SOX_family	Integration	*SOX2*	4	6	7.830	3.18853	（4.490,11.180）	0.790
		SOX4	1					
		SOX15	1					
		SOX7	0					
		SOX17	0					
	non-integration	*SOX2*	10	11	8.455	2.42337	（6.827,10.083）	
		SOX4	0					
		SOX15	0					
		SOX7	0					
		SOX17	1					
NR4A_family	Integration	*NR4A2*	4	5	3.200	5.01996	（-3.030,9.430）	0.517
		NR4A3	1					
	non-integration	*NR4A2*	10	10	1.300	1.94651	（-0.090,2.690）	
		NR4A3	0					

Supplementary Table 9. The immunohistochemical staining of gene families with HPV integration (continuded).

GeneGroup	Groups	GeneSymbol	number of tissue sections	Total number of tissue sections	mean value	standard deviation	95% confidence interval	*p*-value(Mann-Whitney U test)
SangerCancerCensus-others	Integration	*FGFR1*	1	4	3.250	3.94757	（-3.030,9.530）	0.249
		NOTCH1	1					
		IDH2	1					
		ERBB4	1					
		GATA1	0					
		STAG2	0					
		JUN	0					
		ROS1	0					
		ERG	0					
		MAPK1	0					
	non-integration	*FGFR1*	0	6	0.830	1.32916	（-0.560,2.230）	
		NOTCH1	0					
		IDH2	0					
		ERBB4	0					
		GATA1	1					
		STAG2	1					
		JUN	1					
		ROS1	1					
		ERG	1					
		MAPK1	1					
