# Supplementary material for: Human papillomavirus integration perspective in small cell cervical carcinoma
Source: Nat Commun. 2022 Oct 10;13:5968. doi: 10.1038/s41467-022-33359-w (PMC9550834; doi:10.1038/s41467-022-33359-w)
Supplement: Supplementary file 3 — Description of Additional Supplementary Files [file 41467_2022_33359_MOESM3_ESM.pdf]

Supplementary Data 1

Sample information was shown here. NA stands for not available. FF tissue stands for fresh frozen tissue. FFPE tissue stands for formalin-fixed paraffin-embedded tissue. IHC stands for immunohistochemistry. Lymph node ratio (LNR) refers to the ratio of the number of positive lymph nodes to the number of resected lymph nodes. NACT stands for Neoadjuvant chemotherapy.

Supplementary Data 2

WGS data summary.

Supplementary Data 3

WES data summary.

Supplementary Data 4

HPV subtypes from VCS analysis in 150 SCCC FFPE samples

Supplementary Data 5

Virus genome construction in fourteen WGS samples.

1. Sheet_a is about HPV variants identified in all samples.
2. GP6 and EXT primers are from HPV MS detection technology of BGI.
3. Sheet_b is about the mutations used to construct individual HPV genomes.
4. Sheet_c is about Genome walk PCR and assembly of individual HPV genome.
5. Viral loop junction site was in the first PCR and last PCR in each sample.

Supplementary Data 6

HPV integrations from VCS analysis in 150 SCCC FFPE samples.

1. HPV reference: NC_001526.4 for HPV16, NC_001357.1 for HPV18.
2. Case NO. is Sample inner defined.
3. Human gene is annotated by wANNOVAR.
4. Virus breakpoint may have another position enclosed by parentheses (e.g., NO.19 in sample

T016), which is specially extended by one virus genome length for circular reference in bioinformatic analysis.

Supplementary Data 7

Sample subgroups based on HPV integrations from VCS analysis.

1. Cells in blue frame are HPV-integrated genes for sample subtype classification.
2. Cells in red frame are HPV-integrated genes from gene family different from that this sample's subtype relates to.
3. Cells in green frame are HPV-integrated genes from SangerCancerCensus and CancerDependenceMap gene-sets, but exclusive from the five gene families.
4. Is_C means whether given gene belongs to SangerCancerCensus gene-set. Similarly, Is_M for CancerDependenceMap gene-set.

Supplementary Data 8

Details of HPV integrations in fourteen WGS samples.

1. Integration NO. is Sample inner defined.
2. Virus breakpoint may have another position enclosed by parentheses (e.g., NO.6 in sample T004), which is specially extended by one virus genome length for circular reference in bioinformatic analysis.
3. "-" means Not_Found, "<" in Feature section means identical to the left cell.
4. In Fragile Site column, CFS means Common Fragile Sites, RFS means Rare Fragile Sites, and NFR means nonfragile control region.
5. In non B column, IV_R means Inverted Repeat, ST_R means Short Tandem Repeat, GQ_M means G Quadruplex Motif, AP_R means A Phased Repeat, MR_R means Mirror Repeat, DT_R means Direct Repeat, and ZD_M means Z DNA Motif.
6. Only Motifs with consensus sequences longer than or equal to five bases are included in Breakpoint Motif.
7. In Breakpoint Motif column, DPF means DNA polymerase frameshift hotspot, MPR means Murine parvovirus recombination hotspot, VIC means Vaccinia topoisomerase I consensus cleavage site, DHC means Deletion hotspot consensus sequence, DPA means DNA polymerase

arrest site, IHCS means Immunoglobulin heavy chain class switch repeat, TTS means Translin target site, and NRS means Nonamer recombination signal.

8. DnaseSite database was downloaded from ENCODE official website, file name is wgEncodeRegDnaseClusteredV3.bed.gz.

9. HPV reference: NC_001526.2 for HPV16, NC_001357.1 for HPV18.

Supplementary Data 9

Details of Local Haplotypes in fourteen WGS samples.

1. All information are corresponding to Figure 2 and Supplementary Figures 7-19.

2. Segments from human being are in green, and viral segments are in pink.

3. Note that several samples have remarks for explanation in its sheet, e.g., T003.

4. Segments' IDs are single letters, as same as that in Figure 2 and Supplementary Figures 7-19. Upper case for human genome segment, lower case for HPV genome segment.

5. The resolutions of human genome segments used in Figure 2 and Supplementary Figures 7-19 are displayed in each worksheet.

6. Once one segment is reversed complementary connected with other segment(s), its id will be prefixed by "r_".

7. Contigs could repeat several times in one resolved local haplotype of one given sample.

8. Contigs are not phased with each other in resolved local haplotype, see Supplementary Notes 10 and 11.

9. HPV reference: NC_001526.2 for HPV16, NC_001357.1 for HPV18.

10. All HPV-integrated haplotypes shown in this table are Simplest Local Haplotype (Supplementary Notes 10 and 13).

Supplementary Data 10

Shared linked-reads barcodes of pair-wise anchors in local haplotypes.

1. Two types of anchors are listed: JUNC-anchor and POS-anchor (Supplementary Note 13).

2. JUNC-anchors include HPV integrations, viral or host rearrangements.

3. Case NO. is Sample inner defined.

4. In *JUNC-Pwanchor* sheet, columns SegEnd are corresponding to Figure 2 and

Supplementary Figures 7-19. Note that NO.5 case in sample T014 is noted as "-" due to it is out of local haplotype.

5. In *JUNC-Pwanchor* sheet, in columns SegEnd, "_R" mean right end (i.e., 5 primer); "_L" means left end (i.e., 3 primer); and "_M" means middle.

6. In *JUNC-Pwanchor* sheet, JR barc means Barcode count of Junction Reads (i.e., split-reads).

7. For Simu Barcode, please see Supplementary Note 13.

8. HPV reference: NC_001526.4 for HPV16, NC_001357.1 for HPV18.

9. Barcode IDs of each anchor are listed in Supplementary Data 10.

10. SPLH means Simplest Local Haplotype, and RBLH means Random-Best Local Haplotype, please see Supplementary Notes 11 and 13.

Supplementary Data 11

Barcodes of reads supporting anchors in local haplotypes.

1. Two types of anchors are listed: JUNC-anchor and POS-anchor (Supplementary Note 13).

2. JUNC-anchors include HPV integrations, viral or host rearrangements.

3. Case NO. is corresponding to that in Supplementary Data 10.

Supplementary Data 12

Features of HPV integration sites in fourteen WGS samples

1. The sheet single integration shows the features of each single integration sites in HPV-integrated local haplotypes.

2. The sheet pairwise integrations shows the feature combinations of each pairwise integration sites in local haplotypes.

3. Repeated Element includes LTR, LINE, and SINE.

4. Integrated site within two nucleosomes (292nt) from Dnase-I Sites is determined as Y in column Dnase-I Sites.

5. Fragile Site includes CRS and FRS.

Supplementary Data 13

Expressions of TFs on duplicated segments in local haplotypes

1. The sheet TFs in viral enhancer shows FPKM value of TFs in epithelium-specific viral enhancer of viral LCR region reserved in local haplotypes.

2. The sheet TFs in human segments shows FPKM value of TFs in host segments in local haplotypes.

3. The annotated TFs in host segments are downloaded from ENCODE projects. Note that they are combined from experiments of different cell lines.

4. The only TF located in the relevant host segment shows its FPKM or NA (no RNA-seq data), otherwise, just use "-".

5. FPKM values larger than 10 are selectively filled in green.

Supplementary Data 14

Details of HPV-human fusion genes.

1. Inner NO. is Sample inner defined. In several samples, it is discontinuous due to some fusion candidates were filtered out.

2. Columns Virus Region and Human Region are corresponding to Figure 2 and Supplementary Figures 7-19. Note that three samples (T013, T015, and T016) are N/A due to lack of WGS data.

3. In Columns Virus Region and Human Region, "_R" mean right end (e.g., 5 primer); "_L" means left end (3 primer); and "_M" means middle.

4. Only fusions of the third category also need to be validated in DNA level, others are just in cDNA level.

5. Column Detected Integration? shows whether the fusion break-points are exactly same as the HPV integrations on cancer genome, P means need PCR to validate in DNA level.

6. HPV reference: NC_001526.2 for HPV16, NC_001357.1 for HPV18.

Supplementary Data 15

ASEs in local haplotype of HPV integrations in fourteen WGS samples.

1. Ref_F means Forward mapping reads supporting reference allele; "_R" means Reversed mapping; "_S" means Sum of "_F" and "_R" reads.

2. Het means Heterozygous SNP, "LOH" means Loss Of Heterozygosity.

3. Genetic Location, dbSNP ID and Gene Located are from wANNOVAR annotation.

4. Reads counts are obtained from bam files by SamTools mpileup and Bcftools view with defaults options.

5. Clone count means the number of clones successfully performed in experiment.

6. Numbers marked in red means the proportion of ref_S/alt_S calculated from experiments is different from expected.

Supplementary Data 16

Shared 10x barcodes of ASEs and breakages in local haplotypes.

1. Breakages include HPV integrations, viral or host rearrangements.

2. Ref_F means Forward mapping reads supporting reference allele; "_R" means Reversed mapping; "_S" means Sum of "_F" and "_R" reads.

3. dbSNP ID is from wANNOVAR annotation.

4. Case NO.s in columns Shared Barcode Count are corresponding to that in Supplementary Data 10.

5. Note that Shared Barcode Count used the column JR barc of breakages in Supplementary Data 10.

6. Connection Imbalance is calculated by difference between SUM of Ref_allele's shared barcode count and SUM of Alt_allele's, and then devided by SUM of all shared barcode count of this ASE position.

7. In column Imbalance Same Allele?, N/A means Connection Imbalance data is not available (devided by zero), Y means allele that has most barcode shared with local haplotype breakages also has more reads count in RNA-Seq data.

Supplementary Data 17

Barcodes of reads aligned to ASEs in local haplotypes. In column Mapped Orientation, F means forward, R means reversed and complementary.

Supplementary Data 18

Details of SNV and InDel from WGS analysis. The somatic p-value represents significance of

allele frequency difference by one-tailed Fisher's Exact Test (alternative = "greater").

Supplementary Data 19

Details of SNV and InDel from WES analysis. The somatic p-value represents significance of allele frequency difference by one-tailed Fisher's Exact Test (alternative = "greater").

Supplementary Data 20

Details of CNV segment from WGS analysis, Seg.mean is log2 value of CNA ratio.

Supplementary Data 21

Details of CNV segment from OncoScan array, Seg.mean is log2 value of CNA ratio.

Supplementary Data 22

Details of CNV-GISTIC from WGS analysis

Supplementary Data 23

Details of CNV-GISTIC from OncoScan FFPE CNV assay

Supplementary Data 24

DEG in RNA-seq analysis, Fold_change was calculated by software ballgown. The two-sided t.test was performed for each gene to calculate the p-value of differential expression across the normal and tumor groups. 'qval' represents the FDR adjusted p-values.

Supplementary Data 25

GSEA report in RNA-seq analysis, the Enrichment score (ES) has been normalized across the analyzed gene sets to calculate the normalized enrichment score (NES). The nominal p value (NOM p-val) represents the statistical significance of the gene set enrichment score; the false discovery rate adjusted enrichment score is FDR q-val; the familywise-error rate (FWER p-val) estimated the probability of false positive events.

Supplementary Data 26

Details of structural variations from WGS analysis in sixteen SCCC samples.

1. Contents of this table are from fusions.pl script of Meerkat, including 5' end, 3' end, Supporting reads, and Features columns.
2. SV NO. is Sample inner defined.
3. SV relation is for the meerkat-SV-types with pairwise breakpoints, such as inss/o*, del_ins/o*, del_invers and so on.
4. Note that NO.98 in sample T017 is found by PCR validation of virus integration (NO.3 in T017), as its upstream part (chr8:128510364-128510419) is too short (56nt) to be reported by Meerkat.

Supplementary Data 27

Details of human endogenous fusion genes in RNA-seq analysis.

1. Information is based on Ensembl version 75.
2. Fusion NO. is sample inner defined.
3. Introduction of Fusion Type and Transcript Frame Shift is on SOAPfuse SourceForge Wiki Page.
