# Supplementary material for: Human papillomavirus integration perspective in small cell cervical carcinoma
Source: Nat Commun. 2022 Oct 10;13:5968. doi: 10.1038/s41467-022-33359-w (PMC9550834; doi:10.1038/s41467-022-33359-w)
Supplement: Supplementary file 31 — Reporting Summary [file 41467_2022_33359_MOESM31_ESM.pdf]

nature portfolio

Corresponding author(s): Ding Ma

Last updated by author(s): Apr 17, 2022

Reporting Summary

Nature Portfolio wishes to improve the reproducibility of the work that we publish. This form provides structure for consistency and transparency in reporting. For further information on Nature Portfolio policies, see our Editorial Policies and the Editorial Policy Checklist.

Statistics

For all statistical analyses, confirm that the following items are present in the figure legend, table legend, main text, or Methods section.

n/a	Confirmed	
☐	☒	The exact sample size (*n*) for each experimental group/condition, given as a discrete number and unit of measurement
☐	☒	A statement on whether measurements were taken from distinct samples or whether the same sample was measured repeatedly
☐	☒	The statistical test(s) used AND whether they are one- or two-sided<br>*Only common tests should be described solely by name; describe more complex techniques in the Methods section.*
☒	☐	A description of all covariates tested
☒	☐	A description of any assumptions or corrections, such as tests of normality and adjustment for multiple comparisons
☐	☒	A full description of the statistical parameters including central tendency (e.g. means) or other basic estimates (e.g. regression coefficient) AND variation (e.g. standard deviation) or associated estimates of uncertainty (e.g. confidence intervals)
☐	☒	For null hypothesis testing, the test statistic (e.g. F, t, r) with confidence intervals, effect sizes, degrees of freedom and P value noted<br>*Give P values as exact values whenever suitable.*
☒	☐	For Bayesian analysis, information on the choice of priors and Markov chain Monte Carlo settings
☒	☐	For hierarchical and complex designs, identification of the appropriate level for tests and full reporting of outcomes
☐	☒	Estimates of effect sizes (e.g. Cohen's *d*, Pearson's *r*), indicating how they were calculated

Our web collection on statistics for biologists contains articles on many of the points above.

Software and code

Policy information about availability of computer code

Data collection	No software was used.
Data analysis	The high quality reads were aligned to the NCBI human reference genome (hg19) using bwa (v0.7.12, https://github.com/lh3/bwa).We employed MuTect2(v3.5, https://software.broadinstitute.org/cancer/cga/mutect) to detect single nucleotide substitutions and short insertions and deletions. All high confident mutations were annotated with ANNOVAR (V2.4, http://annovar.openbioinformatics.org). We used patchwork(v2.4 , https://patchwork.r-forge.r-project.org/) to perform CNA segmentation, followed by GISTIC2 (v2.0.22, ftp://ftp.broadinstitute.org/pub/GISTIC2.0/GISTIC_2_0_22.tar.gz) to identify significantly altered focal amplification and deletion. We used highly efficient splicing alignment tool HiSAT2 (v2.0.4, ftp://ftp.ccb.jhu.edu/pub/infphilo/hisat2/downloads/hisat2-2.0.4-Linux_x86_64.zip) to carry out RNA-seq data alignment and StringTie (v1.2.3, http://ccb.jhu.edu/software/stringtie/dl/stringtie-1.2.3.Linux_x86_64.tar.gz) to perform transcript assembly and quantification. The ballgown (v1.0.1, https://github.com/alyssafrazee/ballgow) R package was used to perform differential gene expression analysis. HPV variants and integrations were detected by FuseSV (v0.29, https://github.com/deepomicslab/FuseSV). HPV genome rearrangements were detected by seeksv (v1.2.3, https://github.com/qiukunlong/seeksv). Barcoded sequencing data was processed by LongRanger(v2.1.2, https://github.com/10XGenomics/longranger).

For manuscripts utilizing custom algorithms or software that are central to the research but not yet described in published literature, software must be made available to editors and reviewers. We strongly encourage code deposition in a community repository (e.g. GitHub). See the Nature Portfolio guidelines for submitting code & software for further information.

Data

Policy information about availability of data

All manuscripts must include a data availability statement. This statement should provide the following information, where applicable:

- Accession codes, unique identifiers, or web links for publicly available datasets
- A description of any restrictions on data availability
- For clinical datasets or third party data, please ensure that the statement adheres to our policy

The whole genome sequencing data generated in this study have been deposited in GSA (Genome Sequence Archive in BIG Data Center, Beijing Institute of Genomics, Chinese Academy of Sciences, http://gsa.big.ac.cn) under the accession number CRA000349. The RNA-sequencing data, HPV captured sequencing (VCS) data, OncoScan data and whole exome sequencing data generated in this study have been deposited in GSA under the accession number CRA001253. All the other data supporting the findings of this study are available within the article and its supplementary information files.

Field-specific reporting

Please select the one below that is the best fit for your research. If you are not sure, read the appropriate sections before making your selection.

[x] Life sciences [ ] Behavioural & social sciences [ ] Ecological, evolutionary & environmental sciences

For a reference copy of the document with all sections, see nature.com/documents/nr-reporting-summary-flat.pdf

Life sciences study design

All studies must disclose on these points even when the disclosure is negative.

Sample size	Because small cell cervical carcinoma (SCCC) is rare, all available samples were included into the study. We collected fresh frozen or formalin fixed paraffin embedded (FFPE)samples of 214 SCCC patients, which were provided by fifteen collaborating Chinese hospitals in China from 2007 to 2015.
Data exclusions	We use a variety of methods to ensure the reliability of the data analyzed. All data had passed quality inspection before analysis (Supplementary Note 1,2,4).
Replication	For Immunohistochemistry analysis, similar staining results must be observed in over 3 visual fields, and statistic results were provided where available.
Randomization	Randomization was not required, as interventions were not involved in this study.
Blinding	The staffs performing sample preparation , sequencing and outcome assessment were unaware of the sample identity.

Reporting for specific materials, systems and methods

We require information from authors about some types of materials, experimental systems and methods used in many studies. Here, indicate whether each material, system or method listed is relevant to your study. If you are not sure if a list item applies to your research, read the appropriate section before selecting a response.

Materials & experimental systems

n/a	Involved in the study	
[ ]	[x]	Antibodies
[x]	[ ]	Eukaryotic cell lines
[x]	[ ]	Palaeontology and archaeology
[x]	[ ]	Animals and other organisms
[ ]	[x]	Human research participants
[x]	[ ]	Clinical data
[x]	[ ]	Dual use research of concern

Methods

n/a	Involved in the study	
[x]	[ ]	ChIP-seq
[x]	[ ]	Flow cytometry
[x]	[ ]	MRI-based neuroimaging

Antibodies

Antibodies used	rabbit anti-MYC (cat#ZA-0555; dilution:1:100; ZSGB-BIO; CHINA; http://www.zsbio.com/product/ZA-0555), mouse anti-ASCL1 (cat#556604; dilution:1:100, BD, USA; https://www.bdbiosciences.com/zh-cn/products/reagents/immunoassay-reagents/purified-mouse-anti-mash1.556604), mouse anti-INSM1 (cat#sc-271408; dilution:1:100; Santa Cruz; USA; https://www.scbt.com/zh/p/insm1-antibody-a-8), rabbit anti-CHGA (cat#ab283265; dilution:1:100; Abcam; USA; https://www.abcam.cn/chromogranin-a-antibody-rm1025-ab283265.html), rabbit anti-NCAM1 (cat# ab220360; dilution:1:100; Abcam; USA; https://www.abcam.cn/ncam1-antibody-epr21827-ab220360.html), rabbit anti-SYP (cat#17785-1-AP; dilution:1:50; Proteintech; USA; https://www.ptgcn.com/products/SYP-

March 2021

	Antibody-17785-1-AP.htm), rabbit anti-ENO2 (cat#10149-1-AP; dilution:1:50; Proteintech; USA; https://www.ptgcn.com/products/ENO2-Antibody-10149-1-AP.htm), rabbit anti-INPP4B (cat# ab81269; dilution:1:50; abcam; USA; https://www.abcam.cn/inpp4b-antibody-epr3108y-ab81269.html) ,rabbit anti-MYCN (cat# 10159-2-AP; dilution:1:100; Proteintech; USA; https://www.ptgcn.com/products/MYCN-Antibody-10159-2-AP.htm#product-information), mouse anti-FGFR1 (cat# ab829;dilution:1:100; Abcam; USA; https://www.abcam.cn/fgfr1-alpha-antibody-m2f12-ab829.html), rabbit anti-Notch1 (cat# ab52627;dilution:1:100;Abcam; USA; https://www.abcam.cn/notch1-antibody-ep1238y-ab52627.html), rabbit anti-IDH2(cat# ab131263;dilution:1:100;Abcam; USA; https://www.abcam.cn/idh2-antibody-epr7577-ab131263.html), mouse anti-ERBB 4 (cat# ab219208; dilution:1:100; Abcam; USA; https://www.abcam.cn/erbb4--her4-antibody-epr22665-104-ab219208.html), rabbit anti- GATA1 (cat# ab28839; dilution:1:100; Abcam; USA; https://www.abcam.cn/gata1-antibody-ab28839.html), rabbit anti-NR4A3 (cat# ab188752; dilution: 1: 500, Abcam; USA; https://www.abcam.cn/nor1tec-antibody-c-terminal-ab188752.html), rabbit anti-Jun (cat# ab40766; dilution:1:200, Abcam; USA; https://www.abcam.cn/c-jun-antibody-ep693y-ab40766.html), rabbit anti-ROS1 (cat# ab189925; dilution:1:300; Abcam; USA; https://www.abcam.cn/ros1-antibody-epmghr2-ab189925.html), rabbit anti-ERG (cat# ab92513; dilution: 1: 500; Abcam; USA; https://www.abcam.cn/erg-antibody-epr3864-ab92513.html), rabbit anti-SOX17 (cat# ab224637; dilution:1:100; Abcam; USA; https://www.abcam.cn/sox17-antibody-epr20684-ab224637.html), mouse anti-MAPK1 (cat# sc-271269; dilution:1:50; Santa Cruz; USA; https://www.scbt.com/zh/p/erk-1-antibody-g-8), rabbit anti-SOX4 (cat# bs-11208R; dilution:1:50; BIOSS; CHINA; https://www.biossusa.com/products/bs-11208r), rat anti-MYCL (cat# bs-24627R; dilution:1:400; BIOSS; CHINA; https://www.biossusa.com/products/bs-24627r), rabbit anti-STAG2 (cat# 19837-1-AP; dilution:1:50; Proteintech; USA; https://www.ptgcn.com/products/STAG2-Antibody-19837-1-AP.htm), rabbit anti-SOX2 (cat# 11064-1-AP; dilution:1:50; Proteintech; USA; https://www.ptgcn.com/products/SOX2-Antibody-11064-1-AP.htm), rabbit anti-SOX15 (cat# 16725-1-AP; dilution:1:50; Proteintech; USA; https://www.ptgcn.com/products/SOX15-Antibody-16725-1-AP.htm), rabbit anti-Nurr1/NR4A2 (cat# 10975-2-AP; dilution:1:100; Proteintech; USA; https://www.ptgcn.com/products/NR4A2-Antibody-10975-2-AP.htm).
Validation	All primary antibodies are tested and characterized as specific in human tissues, by the manufacturers, and are widely cited. Antibody-specific validations are available as indicated on the manufacturers' web page. Links are listed in the "Antibodies Used" section above.

Human research participants

Policy information about studies involving human research participants

Population characteristics	The number of SCCC patients are 214. Covariate-relevant population characteristics of the SCCC participants include age, FIGO stage, tumor homology,tumor size,lymph node involvement, depth of cervical stromal invasion,vaginal stump invasion,lymphovascular space invasion,neoadjuvant chemotherapy (NACT), HPV typing and neuroendocrine marker expression (SYN,CgA,CD56 and NSE)
Recruitment	Patients diagnosed with SCCC were recruited from fifteen collaborating Chinese hospitals in China from 2007 to 2015 (Supplementary Note 1).
Ethics oversight	The protocol was approved by the Ethics Committee of Tongji Hospital, Tongji Medical College, Huazhong University of Science and Technology, P. R. China. All patients provided written informed consent (Supplementary Note 1).

Note that full information on the approval of the study protocol must also be provided in the manuscript.
